# Reduced genetic variability in a captive-bred population of the endangered Hume’s pheasant (*Syrmaticus humiae*, Hume 1881) revealed by microsatellite genotyping and D-loop sequencing

**DOI:** 10.1371/journal.pone.0256573

**Published:** 2021-08-27

**Authors:** Jitmat Thintip, Worapong Singchat, Syed Farhan Ahmad, Nattakan Ariyaraphong, Narongrit Muangmai, Wiyada Chamchumroon, Klinsak Pitiwong, Warong Suksavate, Sutee Duangjai, Prateep Duengkae, Kornsorn Srikulnath

**Affiliations:** 1 Faculty of Forestry, Department of Forest Biology, Special Research Unit for Wildlife Genomics (SRUWG), Kasetsart University, Bangkok, Thailand; 2 Faculty of Science, Laboratory of Animal Cytogenetics and Comparative Genomics (ACCG), Department of Genetics, Kasetsart University, Bangkok, Thailand; 3 Faculty of Science, Animal Genomics and Bioresource Research Center (AGB), Kasetsart University, Bangkok, Thailand; 4 Faculty of Fisheries, Department of Fishery Biology, Kasetsart University, Bangkok, Thailand; 5 Department of National Park, Wildlife and Plant Conservation, Ministry of Natural Resources and Environment, Bangkok, Thailand; 6 Center of Excellence on Agricultural Biotechnology (AG-BIO/PERDO-CHE), Bangkok, Thailand; 7 Amphibian Research Center, Hiroshima University, Higashihiroshima, Japan; Sant Baba Bhag Singh University, INDIA

## Abstract

Captive breeding programs are crucial to ensure the survival of endangered species and ultimately to reintroduce individuals into the wild. However, captive-bred populations can also deteriorate due to inbreeding depression and reduction of genetic variability. We genotyped a captive population of 82 individuals of the endangered Hume’s pheasant (*Syrmaticus humiae*, Hume 1881) at the Doi Tung Wildlife Breeding Center to assess the genetic consequences associated with captive breeding. Analysis of microsatellite loci and mitochondrial D-loop sequences reveal significantly reduced genetic differentiation and a shallow population structure. Despite the low genetic variability, no bottleneck was observed but 12 microsatellite loci were informative in reflecting probable inbreeding. These findings provide a valuable source of knowledge to maximize genetic variability and enhance the success of future conservation plans for captive and wild populations of Hume’s pheasant.

## Introduction

The most important concerns in the conservation biology of wildlife are a decrease in genetic variability, an increased likelihood of extinction due to changes in land use, over-exploitation of living resources, and urbanization [1,2]. The strongest negative impact on biodiversity is habitat fragmentation, which leads to deterioration of ecosystems and a reduction in populations, which may lead to the extinction of species [3]. Isolated wildlife populations show increased inbreeding, which results in a decline of fitness [4]. Efficient and competent captive-breeding strategies are required to protect species from population decline and extinction [5].

Hume’s pheasant (*Syrmaticus humiae*, Hume 1881) is distributed in specific habitats throughout the hills of North-eastern India, Northern and Western Myanmar, South-western China and Northern Thailand between 1,200 and 2,285 meters above sea level [6,7]. The remaining Thai wild population is roughly estimated at between 200 and 500 individuals [8]. Given heavy exploitation and habitat loss over the last three decades, populations of Hume’s pheasant have decreased dramatically across much of its known range. Illegal hunting is also ongoing [9]. Hume’s pheasant is listed in Appendix 1 of the Convention on International Trade in Endangered Species of Wild Fauna and Flora, and is classified as globally Near Threatened by the International Union for Conservation of Nature (IUCN) [10]. To increase the population size of Hume’s pheasant, a captive breeding program is considered to be a viable option. The Thai government announced a list of protected wildlife species under the Wildlife Preservation and Protection Act of 2019. A captive breeding program was established outside their natural habitat at the Doi Tung Wildlife Breeding Center (20°18’47.016" N, 99°49’01.812" E) in 1960. However, the existing captive breeding program was conducted without genetic monitoring, with inbreeding and low genetic variation in the small founder population, and currently a small captive population. Deleterious effects of inbreeding frequently arise in small captive populations, which leads to a decline in fitness and inbreeding depression [11]. Maintenance of genetic diversity and demographic security are thus important goals for long-term conservation and population management. High genetic variability with a large effective population size will encourage the success of future management options to ensure the expansion of captive populations. In this scenario, the importance of genetic monitoring of the captive Hume’s pheasant population was addressed in the present study by screening genetic variation using analyses of 12 microsatellite loci and partial mitochondrial (mt) D-loop sequences. Our aims were (i) to establish genetic information for the current captive population, and (ii) to formulate a breeding management plan as a genetically cognizant restocking program. Strategies of conservation management are designed to minimize the likelihood of population extinction. The present study comprised a genetic assessment of a captive population in order to optimize future expansion.

## Materials and methods

### Specimen collection

Feather samples were collected from 82 Hume’s pheasant individuals (44 males and 38 females) at the Doi Tung Wildlife Breeding Center (20°18’47.016" N, 99°49’01.812" E) in Thailand between October and November 2020. The original founder sources are unknown and wildlife staff did not track label information of mother/offspring. Detailed information on the sampled individuals is presented in S1 Table. The sex of each individual was identified by morphological observation (S2 Table). Genomic DNA was extracted from the basal tips of feather rachises (approximately 1–2 mm long) using the G-spin™ Total DNA Extraction Kit (Favorgen Biotech Corp., Ping-Tung, Taiwan), and used as a template for microsatellite genotyping and mt D-loop sequencing. This research was conducted under the authority of the Department of National Parks, Wildlife and Plant Conservation (DNP) and the Ministry of Natural Resources and Environment, Thailand. Animal care and all experimental procedures were approved by the Animal Experiment Committee, DNP (Approval No. TS.0909.704/2932, 2/7/2015 following the annual physical examination protocol) and Kasetsart University (Bangkok, Thailand; Approval No. ACKU63-SCI-022), and were conducted in accordance with the Regulations on Animal Experiments at Kasetsart University.

### Microsatellite genotyping

Twelve microsatellite primer sets developed originally from Hume’s pheasant were sourced from [12] (S3 Table). The 5′-end of the forward primer of each set of primers was labeled with a fluorescent dye (6-FAM or HEX; Macrogen Inc., Seoul, Korea). PCR amplification was performed using 15 μl of 1× ThermoPol buffer containing 1.5 mM MgCl_2_, 0.2 mM dNTPs, 5.0 μM primers, 0.5 U *Taq* polymerase (Apsalagen Co., Ltd., Bangkok, Thailand), and 25 ng genomic DNA. The PCR protocol was as follows: initial denaturation at 95°C for 3 min, followed by 35 cycles of 95°C for 45 s, 50–58°C for 45 s (S3 Table), and 72°C for 3 min, with a final extension at 72°C for 10 min. The PCR products were detected by electrophoresis in 1% agarose gel. To decrease the influence of false alleles caused by the failure of PCR amplification, experiments were performed at least in triplicate for each sample. A negative control specimen was prepared for each experiment. The absence of PCR products was also checked by 1% agarose gel electrophoresis after PCR. Analysis of fluorescent DNA fragment length was subsequently performed using an ABI 3730XL Automatic Sequencer (Applied Biosystems, Foster City, CA, USA) and the DNA sequencing service of Macrogen Inc. Allelic size was determined using Peak Scanner version 1.0 (Applied Biosystems). Genotypic data generated in this study were deposited in the Dryad Digital Repository Dataset, https://doi.org/10.5061/dryad. qv9s4mwdq.

### Microsatellite data analysis

We followed the same approaches as those used in previous studies of Asian woolly-necked stork (*Ciconia episcopus*, Boddaert 1783), Chinese goral (*Naemorhedus griseus*, Milne-Edwards 1871), and water monitor lizards (*Varanus salvator macromaculatus*, Laurenti 1768) [13–16]. Allelic frequency, number of alleles (*A*), effective number of alleles (*N*_a_), observed heterozygosity (*H*_o_), expected heterozygosity (*H*_e_), and linkage equilibrium were calculated using Arlequin version 3.5.2.2 [17]. Given that the population was small, deviations from the Hardy-Weinberg equilibrium were evaluated at each locus by the Markov chain Monte Carlo (MCMC) approximation of Fisher’s exact test using the “genepop” function implemented in the package “stats” with R version 3.5.1 [18–20]. Welch’s *t*-test, which does not assume equal variance between samples, was used to test for significant differences between *H*_o_ and *H*_e_ using the “t.test” function in the package “stats” using R version 3.6.3 [20,21]. Allelic richness (AR) was calculated using FSTAT version 2.9.3 [22], and the mean number of effective alleles was derived using GenAlEx version 6.5 [23]. Micro-Checker version 2.2.3 was used to identify null allelic markers [24]. Polymorphic information content (PIC) was estimated using the Excel Microsatellite Toolkit [25] and calculated for each locus. Shannon’s information index (*I*) and a fixation index (*F*) were calculated for each locus of the population using GenAlEx version 6.5 [23]. Effective population size (*N*_e_) was estimated as the number of breeding individuals that contributed to the population using the linkage disequilibrium method in NeEstimator version 2.01 [26].

To consider the possibility of sibling or parent-offspring pairs in the captive population, we determined whether the captive-population individuals were more closely related than random unrelated individuals. Relatedness (*r*) values were calculated for all pairs (comprising female-female, male-male, and male-female pairs), and mean pairwise *r* values based on allelic frequencies in the population were calculated at captivity using GenAlEx version 6.5 [23]. Individual and overall inbreeding coefficients (*F*_IS_) with 95% confidence intervals (CIs) were calculated using the LynchRt estimator [27] as implemented in COANCESTRY version 1.0.1.9 [28]. Examination of values of *r* and *F*_IS_ was conducted under the assumption that the mean did not differ significantly from those of random assortments of unrelated individuals. Parentage analysis and the probability that two individuals shared the same genotype were calculated using COLONY version 2.0.6.6 [29] and GIMLET version 1.3.3 [30], respectively. Mendelian inheritance was examined for every locus. Individuals who shared alleles from their putative parents at all loci were considered actual offspring of the pair. Cases in which pairing failed to match any of the two alleles of the putative parents at two or more loci were considered to be extra-pair paternity or new wild individuals.

The condition of heterozygosity abundance and changes in allelic frequency appropriations were examined in hereditarily bottlenecked populations using Bottleneck version 1.2.02 [31]. The Wilcoxon signed-rank test, with a two-phase mutation model (TPM) and stepwise mutation model (SMM), was used to derive probabilities for excessive heterozygosity as a result of the small sample sizes for loci and small individual sample size. The TPM was implemented with 95% single-step mutations and 5% multistep mutations, with variance among multiple steps set at 12 [32] This test detects relatively short-term bottleneck events. To test for relatively long-term bottleneck events, the *M* ratio test [33] was performed using Arlequin version 3.5.2.2 [17]. The *M* ratio is the mean number of alleles in a population divided by the allelic size range and indicates reductions in both recent and historical population sizes. Principal Coordinates Analysis (PCoA) was performed to assess the overall relationship across individuals in the captive population using GenAlEx version 6.5. The model-based clustering method implemented in STRUCTURE version 2.3.3 was used to determine population structure [34]. Run-length was set to 100,000 MCMC replicates after a burn-in period of 100,000 generations, using correlated allelic frequencies under a straight admixture model. The number of clusters (*K*) varied from 1 to 25, with 25 replicates for each value of *K*. The most probable number of clusters was determined by plotting the log likelihood of the information (ln Pr (*X*|*K*)) [34] over the scope of tested *K* values before choosing the optimal *K* value at which ln Pr (*X*|*K*) settled. The Δ*K* strategy was applied using Structure Harvester [35].

### Mitochondrial D-loop sequencing

The mt D-loop sequence was selected as a suitable region for estimation of the genetic variability of Hume’s pheasant [36,37]. The mt D-loop fragments were amplified using the primers PHDL (5′-AGGACTACGGCTTGAAAAGC-3′) and PHDH (5′-CATCTTGGCATCTCAGTGCC-3′) [38]. PCR amplification was performed using 20 μl of 1× ThermoPol buffer containing 1.5 mM MgCl_2_, 0.2 mM dNTPs, 5.0 μM primers, 0.5 U *Taq* polymerase (Apsalagen Co., Ltd.) and 25 ng genomic DNA. The PCR conditions were as follows: initial denaturation at 95°C for 3 min, followed by 35 cycles of 95°C for 45 s, 56°C for 45 s, and 72°C for 3 min, and final extension at 72°C for 10 min. The PCR products were purified using the FavorPrep GEL/PCR Purification Mini Kit (Favorgen Biotech Corp.). Nucleotide sequences of the DNA fragments were determined by the DNA sequencing service of First Base Laboratories Sdn Bhd (Seri Kembangan Selangor, Malaysia). The BLASTn tool (http://blast.ncbi.nlm.nih.gov/Blast.cgi) was used to search nucleotide sequences in the National Center for Biotechnology Information database to confirm the identity of the DNA fragments amplified in this study. Generated sequences were deposited in the DNA Data Bank of Japan (S4 Table).

### Mitochondrial D-loop data analysis

Multiple sequence alignment of the 100 partial mt D-loop sequences was generated using the default parameters of Molecular Evolutionary Genetics Analysis X (Center for Evolutionary Functional Genomics, The Biodesign Institute; [39]). All unalignable and gap-containing sites were removed carefully and trimmed manually from the data sets. Estimates of haplotype (*h*) and nucleotide (π) diversity [40] number of haplotypes, and mean number of nucleotide differences were calculated based on the mt D-loop sequences as implemented in DnaSP version 5 [41]. A statistical parsimony network of the consensus sequences was constructed using the Templeton, Crandall, and Sing algorithm implemented in PopART version 1.7 to examine haplotype grouping and population dynamics [42]. Demographic history was also determined using the statistical test of neutrality. Tajima’s D* [43], Fu and Li’s D* and F* tests [44], and Fu’s *F*_s_ [45] were calculated using Arlequin version 3.5.2.2 [17]. Ramos-Onsins and Rozas’s *R*_2_, which has greater statistical power for small sample sizes, was calculated using DnaSP version 6 [41,46]. The significance of the differences among these values was determined using 10,000 coalescent simulations in accordance with the recommended software parameters.

The mismatch distribution approach, in which an observed frequency distribution of pairwise nucleotide differences is obtained among individuals with expected distributions from an expanding population (small raggedness index) or a stationary population (large raggedness index), was performed to test for the genetic signatures of historical population expansion within the captive populations [47,48]. These models were used to estimate the parameters of population expansion using a generalized least-squares approach and to compute their CIs by bootstrapping (10,000 replicates) as implemented in DnaSP version 5 [41]. Phylogenetic analysis was performed using Bayesian inference with MrBayes version 3.2.6 [14,15,49]. The outgroup retrieved additional data from the GenBank database and queried for the following mt D-loop sequences of rock pigeon (*Columba livia*, Gmelin 1789, GenBank accession number: FJ792695.1). The best-fit model of DNA substitution was determined for each genetic region using Kakusan4 [50]. The MCMC process was used to run four chains simultaneously for one million generations. After stabilizing the log-likelihood value, a sampling procedure was performed every 100 generations to obtain 10,000 trees, and a majority-rule consensus tree with mean branch lengths was generated. All sample points were discarded as burn-in before attaining convergence, and the Bayesian posterior probability in the sampled tree population was calculated as a percentage.

## Results

### Genetic variability of the Hume’s pheasant captive population based on microsatellite data

Eleven captive individuals were genotyped. Ninety-one alleles were observed among all loci, with mean number of alleles per locus of 7.500 ± 0.802 (Tables 1 and S5). Null alleles were observed at all microsatellite loci, and all markers listed were similarly treated. All allelic frequencies showed significant departures from the Hardy-Weinberg equilibrium of the captive population, with multiple lines of evidence for linkage disequilibrium (S6 Table). Consequently, the population exhibited *F* values of 0.847 ± 0.053. The PIC ranged from 0.048 to 0.749, and *I* ranged from 0.140 to 1.709 (S5 Table). The *H*_o_ values ranged from 0.00 to 0.122 (mean ± standard deviation [SD]: 0.039 ± 0.011) and the *H*_e_ values ranged from 0.048 to 0.780 (mean ± SD: 0.435 ± 0.071) (Tables 1, S5 and S7). Welch’s *t*-test showed that *H*_o_ was significantly different from *H*_e_ in the population (*H*_o_ = 0.039 ± 0.011, *H*_e_ = 0.435 ± 0.071, *t* = -5.514, df = 11, *p* < 0.01). The AR value of the population was 7.500 ± 0.802. The standard genetic diversity indices are summarized in Tables 1 and S5.

**Table 1:** Genetic diversity among 82 Hume’s pheasant (*Syrmaticus humiae*, Hume 1881) individuals based on 12 microsatellite loci.

Pop	Locus	*N*	*N*_a_	AR	*N*_e_	*I*	*H*_o_	*H*_e_	*M* ratio	PIC	*F*
DTP	Mean	82	7.500	7.500	2.190	0.959	0.039	0.435	0.048	0.411	0.847
	S.D.	0	0.802	0.802	0.324	0.159	0.011	0.071	0.019	0.237	0.053

*Significant: *p* < 0.05.

Sample size (*N*); number of alleles (*N*_a_); allelic richness (AR); number of effective alleles (*N*_e_); Shannon’s information index (*I*); observed heterozygosity (*H*_o_); expected heterozygosity (*H*_e_); *M* ratio test (*M* ratio); polymorphic information content (PIC); fixation index (*F*); *p*-value for comparison with the Hardy-Weinberg equilibrium (*p* < 0.05).

A pairwise test was performed to determine the level of relatedness between individuals in the captive population. The mean pairwise *r* value of the Hume’s pheasant pairs among the 82 sampled individuals was -0.006 ± 0.089. A total of 2,523 pairs showed -0.25 < *r* < 0.25 and 798 pairs showed 0.25 < *r* (S8 Table), which indicates that a proportion of the individuals in the population was closely related (*r* > 0.25). The mean *F*_IS_ was 0.153 ± 0.179, with individual values of *F*_IS_ ranging from 0.007 to 1.149 (S9 Table). The *N*_e_ for individuals that contributed genetically to the current population was 39.500 (95% CI: 28.500–57.100) (Table 2). Simultaneously, parentage analysis of individuals in the captive population revealed that approximately one-seventh of all the Hume’s pheasant, or at least five of them, originated from four breeding pairs (12.821%) (S10 Table). All paternities were assigned unequivocally. The combined likelihood of rejection for the microsatellites utilized was evaluated at 0.950. The probability of two Hume’s pheasant sharing an indistinguishable genotype was assessed at 3.920 × 10^−1^ (S11 Table).

**Table 2:** Inbreeding coefficients, relatedness, effective population size, and ratio of effective population size and census population (*N*_e_*/N*) of Hume’s pheasant (*Syrmaticus humiae*, Hume 1881) captive population at the Doi Tung Wildlife Breeding Center.

Locality	*N*	*F*_IS_	Relatedness (*r*)	Estimated *N*_e_	95% CIs for *N*_e_	*N*_e_*/*N
Doi Tung Wildlife Breeding Center	82	0.889 ± 0.037	-0.010 ± 0.055	39.5	28.5–57.1	0.482

Estimates were calculated using NeEstimator version 2.1, COANCESTRY version 1.0.1.9, GenAlEx version 6.5. Detailed information for all S. *humiae* individuals is presented in Supplementary S2 Table. Sample size (*N*); inbreeding coefficient (*F*_IS_); effective population size (*N*_e_).

**Fig 1:**
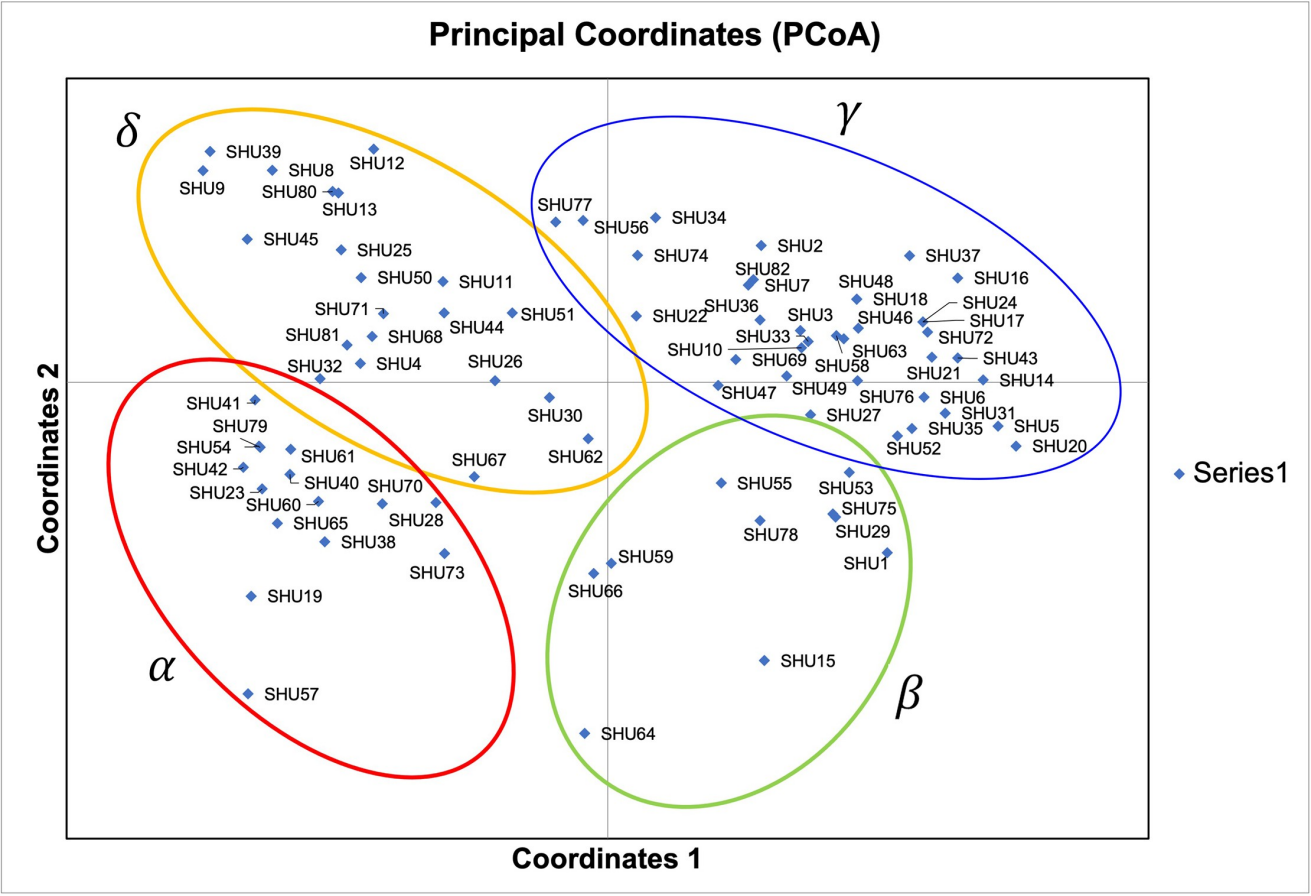
Principal coordinates analysis of Hume’s pheasant (*Syrmaticus humiae*, Hume 1881) captive population at the Doi Tung Wildlife Breeding Center. Detailed information for all Hume’s pheasant individuals is presented in S2 Table.

**Fig 2:**
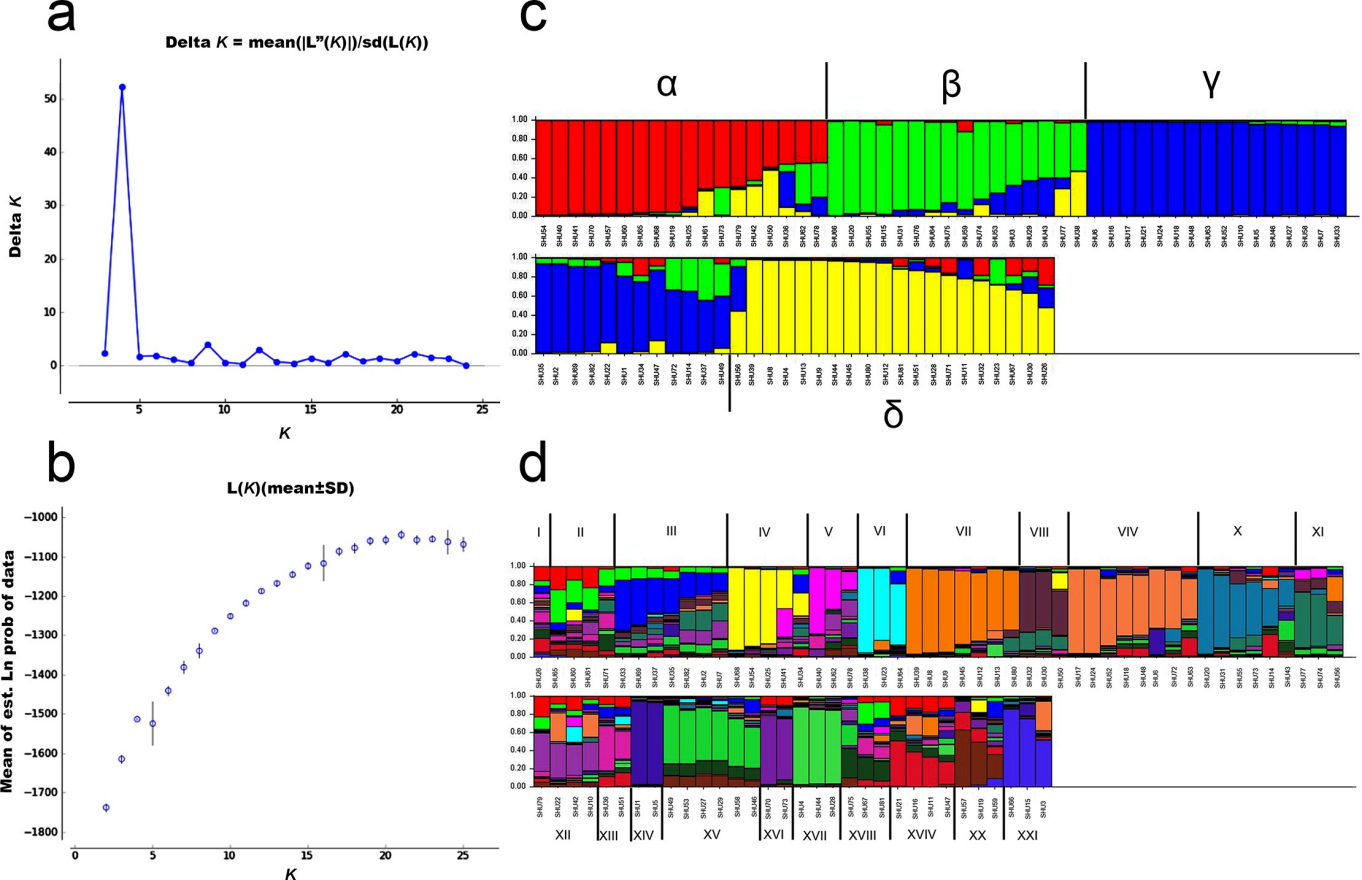
Population structure of the captive population of 82 individuals of Hume’s pheasant (*Syrmaticus humiae*, Hume 1881). (a). Evanno’s Δ*K* graph (b). Mean ln P (*K*) graph, and Structure bar plots depicting model-based clustering results for inferred *K* = 4 (c) and *K* = 21 (d). Inferred genetic clusters are displayed as different colors. Each vertical bar on the *x*-axis represents an individual, and the *y*-axis presents the proportion of membership (posterior probability) in each genetic cluster. Recovered Hume’s pheasants are superimposed on the plot, with black vertical lines indicating the boundaries. Detailed information for all Hume’s pheasant individuals is presented in S2 Table.

The Wilcoxon signed-rank tests for recent population bottlenecks gave an SMM and a TPM of 1.000 in the captive population (normal L-shaped mode shift). The *M* ratio of the population mean 0.048 ± 0.019 (Tables 1 and S5). The *M* ratio values were lower than the 0.680 threshold identified by [33], which indicates the presence of a historical reduction in population size. PCoA revealed that the first, second, and third principal components accounted for 12.840%, 22.380%, and 30.390% of the total variation, respectively, and provided support for four tentatively differentiated Hume’s pheasant groups (α, β, γ, and δ) (Fig 1). Bayesian structural analysis revealed the highest posterior probability, with one peak (*K* = 4) on the basis of Evanno’s Δ*K*, with all Hume’s pheasant individuals grouped into four clusters (α, β, γ, and δ) (Fig 2). By contrast, Bayesian structural analysis based on the mean ln P (*K*) revealed one peak (*K* = 21), which suggests 21 clusters (I–XXI) (Fig 2).

### Genetic variability of the captive population based on mitochondrial haplotype analysis

**Fig 3:**
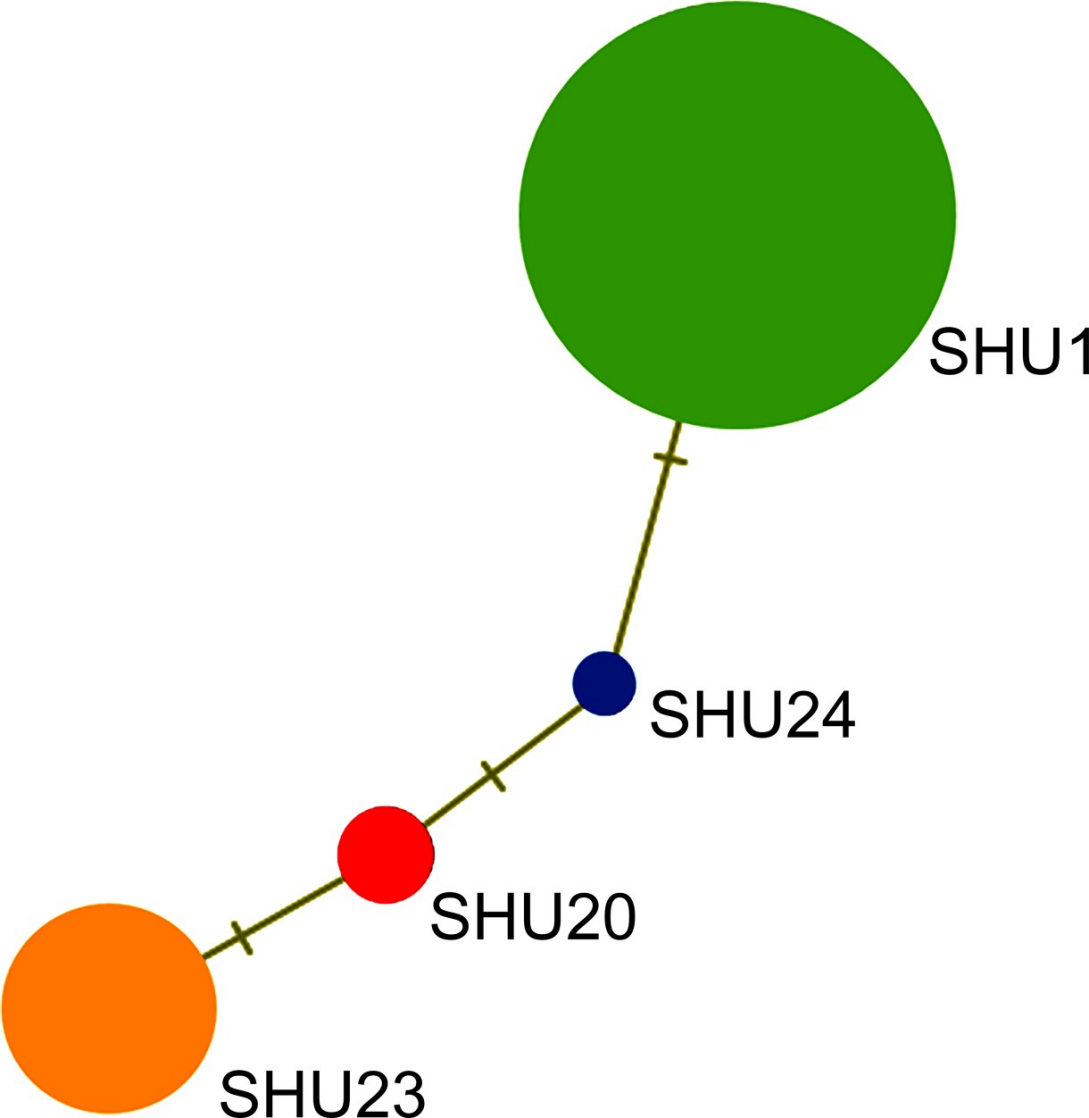
Haplotype network based on sequence data for the mitochondrial D-loop region of Hume’s pheasant (*Syrmaticus humiae*, Hume 1881) from the captive population at the Doi Tung Wildlife Breeding Center.

The amplicon length and alignment length of the mt D-loop sequences were approximately 801 base pairs with four haplotypes resolved. Overall haplotype and nucleotide diversities of the mt D-loop sequences were 0.365 ± 0.004 and 0.006 ± 0.004, respectively (S12 Table). A complex haplotype network was constructed from the large number of detected polymorphic sites and haplotypes (Fig 3). Haplotype networks from the mt D-loop sequence populations showed four clear haplogroups (Fig 3). The most common haplotype of the mt D-loop from Hume’s pheasant differed from that of SHD1 by one mutational step, and one haplotype was shared among individuals.

Five different tests of neutrality were used to estimate the historical population expansion. The mean Tajima’s D* value was 1.109, *p* = 0.852, and the mean Fu and Li’s F* value was 1.090, *p* = 1.000. The mean Fu and Li’s D* value was 0.844, *p* = 1.000. The Ramos-Onsins and Rozas’s *R*_2_ value was 0.161, *p* = 1.000 (S13 Table). We then compared the observed frequency distribution of pairwise nucleotide differences among individuals within the population. Charts of the mismatch distributions for the captive population were unimodal and the raggedness index was 0.480 (*p* < 0.05).

## Discussion

Based on current rates of deforestation, as many as 30% of Southeast Asia’s bird species are predicted to become globally extinct this century [51]. Hunting and habitat loss have been identified as major threats to almost all bird species, including Hume’s pheasant. Forest is mainly lost through tree felling and clearance for jhum cultivation. Closed-canopy forest cover declined from 43% to 21% between 1972 and 1995 [52]. Habitat fragmentation also causes population fragmentation, leading to decreased genetic variability [53,54]. Conservation efforts are impeded by the lack of current Hume’s pheasant distribution and captive population data. Individual resources are limited and it is essential to prioritize activities to maximize both the quality of genetic resources of Hume’s pheasant and the return on effort. Development of the Hume’s pheasant captive breeding project at the Doi Tung Wildlife Breeding Center is robust, with a suitable number of Hume’s pheasants required before release, in order to increase the wild population in Thailand. Genetic monitoring and an effective breeding plan are essential to improve the retention of high genetic variation and contribute to adaptive management decisions [55].

The current Hume’s pheasant captive population exhibited high AR values compared with those of other bird projects [14,56]. This might be a result of the breadth of the initial founding population [7]. However, a low number of mt D-loop haplotypes was observed in the captive population, combined with low haplotype and nucleotide diversity. Compared with other bird reports such as *Syrmaticus humiae* in China (*h* = 0.85, n = 73) [36], *Tragopan caboti* (*h* = 0.97, n = 53) [57], *Bambusicola thoracica* (*h* = 0.79, n = 180) [58] and *Syrmaticus ellioti* (*h* = 0.992, n = 33) [59], the level of genetic diversity of Hume’s Pheasant in this study was low. This result suggests that the founder population might have contained biased sex individuals, and points to the loss of genetic variation through genetic drift, wherein a founding population was likely composed of related female individuals. Unfortunately, the source of the captive population is unknown. There may therefore be a risk in performing captive mating selection, with development of inbreeding depression in a small number of generations. The expected heterozygosity *H*_e_ was significantly higher than *H*_o_ in the captive population of Hume’s pheasant. Similar cases were also observed in the captive populations of cracids [60] and Black-fronted piping guan (*Aburria jacutinga*, Spix, 1825) [61], which is suggestive of possible inbreeding owing to the small population size. This result is consistent with the positive *F*_IS_ value (mean = 0.153 ± 0.179) and the mean *r* values in the captive population. The Hume’s pheasant captive population may have undergone a large number of generations in captivity. The similarity in genetic profiles may result from different captive population origins, with distribution of the founding individuals restricted to the northern region of Thailand [8]. This was also observed in the present study in which the *M* ratio signaled a historical reduction in population size. Bottlenecks with low genetic diversity often occur when a small number of founders are taken from a declining wild population, which results in poor breeding success [62]. The nature of the population at drift-mutation equilibrium without expansion was supported by the mt D-loop neutrality test, the significant ragged shape, and the Wilcoxon signed-rank tests for the captive population. However, the mismatch distribution was unimodal, which is indicative of rapid population expansion. A multimodal mismatch distribution generally indicates a diminished population size or structured size, whereas a ragged distribution suggests that the lineage was widespread [17,47,63]. The captive population may have originated from wild-caught Hume’s pheasants that subsequently bred within the captive breeding program to produce a large number of individuals, thus reflecting recent expansion. We identified at least 39 individuals to be effective in transferring genetic components to the next generation. An estimate of the ratio of *N*_e_ to the consensus population (*N*) enabled us to understand the population fitness, including the risk arising from genetic factors [64]. Here, the *N*_e_ and *N*_e_/*N* of the captive population were extremely low and have generally remained low, relative to those of captive populations of other birds [65]. This might result from rapid proliferation of individuals with small *N*_e_ or *N*_e_/*N*. This finding suggests there is a low potential for recovery of the Hume’s pheasant population in the wild. However, sampling error might have occurred as a consequence of the small sample size [66]. Null alleles are frequently observed at microsatellite loci amplified by microsatellite primers developed from different species [67]. An increase in *N*_*e*_ and a management strategy that includes new introductions to the captive population are required to mitigate genetic drift.

The primary goal of the captive breeding program is to develop a self-sustaining population by minimizing undesirable genetic changes as a result of genetic drift in the captive environment, avoiding the deleterious effects of inbreeding depression, and maintaining a novel perspective on genetic management. Records of pedigree and breeding plans are important for captive management to reduce substantial errors in data assessments of specific mating events, and to minimize parentage exclusions and inbreeding [68]. Analysis of the captive population structure revealed four tentative subpopulations based on PCoA and Bayesian structural analyses (*K* = 4), although different Bayesian structural analyses (*K* = 21) showed 21 subgroups that were likely to be scattered. There is a strong risk that a breeding plan for captive Hume’s pheasant individuals will result in inbreeding depression. Careful examination of intraspecific genetic variability and formulation of breeding plans for species with low reproductive rates [69], or mating systems in which not all individuals contribute their genes to the next generation, can reduce the presence of inbreeding depression and genetic drift in captivity, and allow more adaptive management decisions to be made. To include more effective criteria for mating selection, consideration of different mt D-loop haplotypes is strongly recommended, together with profiling of microsatellite genotype structure and the relatedness of individuals. The finite availability of captive resources strictly limits the population size and number of individuals in the captive population that can be managed, whereas loss of genetic variability is a function of time. Collaboration with national administrative entities (DNP and the National Research Council of Thailand) is necessary to document and monitor the long-term effects of the proposed strategy on the diversity of Hume’s pheasant individuals.

A primary policy instrument to mitigate the loss of biodiversity and reduce ecological degradation is the creation of statutorily protected or conservation areas. However, the level of protection afforded by such areas is widely acknowledged to be inadequate to achieve biodiversity protection goals in multiple dimensions [70,71]. Conservation success requires an effective strategy for rapid action, such as the use of captive populations as a gene pool source. The process includes the maintenance of effective captive populations. Ideally, preservation of 90% heterozygosity of the founding population will endure over a period of several years; however, it might also depend on generation time and sex behavior [15,72]. Hume’s pheasant exhibits an approximately ten-year generation time and polygamous mating behavior. However, the captive population exhibits a state of low genetic variability, which reflects serious foreseeable levels of inbreeding. We strongly recommend improvement of the captive population gene pool by introducing newly captured wild individuals. This strategy will allow new breeding programs to enhance genetic variability and increase *N*_e_ or *N*_e_/*N* in the captive population. International collaboration to exchange Hume’s pheasant individuals might be an alternative means of enhancing genetic variability in the captive population. Alternatively, the current rapid development of biological technology, especially long-term storage by cryopreservation, will reduce dependence on populations of living Hume’s pheasants for the preservation of gene pools. Once the habitats of Hume’s pheasant are destroyed, they are extremely difficult to rehabilitate within a short period. Therefore, emergency measures are needed to preserve valuable genetic resources of Hume’s pheasants for a limited effective population size [1]. Several techniques for conservation of genetic resources are available, including the formation of biobanks by cryopreservation, assisted reproductive techniques such as artificial insemination, embryo transfer, *in vitro* fertilization, cloning using somatic cell nuclear transfer, and biobanking or genome resource banking [73]. Experiences gained in other captive breeding programs may be informative to improve our understanding of these matters. Consequently, the research priorities should be centered on (1) improvement of genetic variability in the captive population, (2) initiation and coordination of long-term monitoring of populations across Southeast Asia, and (3) assessment of the impact of hunting and habitat fragmentation. Research output based on these priorities will identify Hume’s pheasant populations in need of protection and provide a mechanism to assess the success of intervention in conservation.

## Conclusions

This study of genetic monitoring of a captive population of Hume’s pheasant provides useful information for conservation management officers. Recommended action plans for mating selection in breeding programs can be adjusted and applied to the captive population. The assessment of genetic variability is an important approach to maximize reproductive success and promote genetic variation in captive-bred individuals. Long-term maintenance of populations requires the implementation of a precise genetic breeding plan, accompanied by research that examines synergistic impacts, together with the twin crises of climate change and loss of biodiversity. Results can be used to modify and improve the implementation of effective measures. Given the small sample size, our results should be viewed with caution before implementation of a strict management or conservation strategy, to avoid genetic drift and ensure that a high proportion of the source variation is stabilized to minimize the loss of genetic diversity. Implementation should be accompanied by research to examine impacts, with results used to modify and improve the implementation of effective measures.

## Supporting information

S1 Table Details of the Hume’s pheasant (*Syrmaticus humiae*, Hume 1881) captive population studied. (DOCX)

S2 Table Summary of Hume’s pheasant (*Syrmaticus humiae*, Hume 1881) individuals sampled. (DOCX)

S3 Table Primers for microsatellite loci and partial mitochondrial D-loop used in the study. (DOCX)

S4 Table Summary accession number of mitochondrial D-loop sequences of *Syrmaticus humiae* (Hume, 1881) population. (DOCX)

S5 Table Genetic diversity of 82 Hume’s pheasant (*Syrmaticus humiae*, Hume 1881) individuals based on 12 microsatellite loci. Detailed information for all individuals is presented in S2 Table.
(DOCX)

S6 Table Pairwise differentiation of linkage disequilibrium of Hume’s pheasant (*Syrmaticus humiae*, Hume 1881) individuals at the Doi Tung Wildlife Breeding Center based on 12 microsatellite loci. Numbers indicate *p*-values with 110 permutations.
(DOCX)

S7 Table Observed and expected heterozygosity of Hume’s pheasant (*Syrmaticus humiae*, Hume 1881) based on 12 microsatellite loci at the Doi Tung Wildlife Breeding Center and genetic bottlenecks for all individuals. Data were calculated using Bottleneck version 1.2.02. (Cornuet and Luikart, 1996). Detailed information for all individuals is presented in S2 Table.
(DOCX)

S8 Table Pairwise genetic relatedness (*r*) for 82 Hume’s pheasant (*Syrmaticus humiae*, Hume 1881) individuals. Detailed information for all individuals is presented in S1 Table.
(DOCX)

S9 Table Pairwise inbreeding coefficients (*F*_IS_) for 82 Hume’s pheasant (*Syrmaticus humiae*, Hume 1881) individuals. Detailed information for all individuals is presented in S1 Table.
(DOCX)

S10 Table Parentage analysis of 82 Hume’s pheasant (*Syrmaticus humiae*, Hume 1881) individuals. Detailed information for all individuals is presented in S2 Table.
(DOCX)

S11 Table Probability of identity estimated using Gimlet version 1.3.3 (Valière, 2002) of Hume’s pheasant (*Syrmaticus humiae*, Hume 1881) individuals based on 12 microsatellite loci. Detailed information for all individuals is presented in S2 Table.
(DOCX)

S12 Table Mitochondrial D-loop sequence diversity for the Hume’s pheasant (*Syrmaticus humiae*, Hume 1881) population. (DOCX)

S13 Table Neutrality tests of mitochondrial D-loop sequence for the Hume’s pheasant (*Syrmaticus humiae*, Hume 1881) population. (DOCX)
