# Supplementary material for: Reduced genetic variability in a captive-bred population of the endangered Hume’s pheasant (Syrmaticus humiae, Hume 1881) revealed by microsatellite genotyping and D-loop sequencing
Source: PLoS One. 2021 Aug 27;16(8):e0256573. doi: 10.1371/journal.pone.0256573 (PMC8396778; doi:10.1371/journal.pone.0256573)
Supplement: S1 Table — (DOCX) [file pone.0256573.s001.docx]

**S1 Table** **Details of the Hume’s pheasant (*Syrmaticus humiae,* Hume 1881) captive population studied.**

| Locality | Longitude | Latitude | Amount | Sex | |
| --- | --- | --- | --- | --- | --- |
|  |  |  |  | Male | Female |
| Doi Tung Wildlife Breeding Center | 20°18' 47.016" | 99°49' 1.812" | 82 | 44 | 38 |
