# Supplementary material for: Reduced genetic variability in a captive-bred population of the endangered Hume’s pheasant (Syrmaticus humiae, Hume 1881) revealed by microsatellite genotyping and D-loop sequencing
Source: PLoS One. 2021 Aug 27;16(8):e0256573. doi: 10.1371/journal.pone.0256573 (PMC8396778; doi:10.1371/journal.pone.0256573)
Supplement: S2 Table — (DOCX) [file pone.0256573.s002.docx]

**S2 Table Summary of Hume’s pheasant (*Syrmaticus humiae,* Hume 1881) individuals sampled.**

| No. | Code | Type | Sex | Code ID | Locality | Original |
| --- | --- | --- | --- | --- | --- | --- |
| 1 | SHU1 | Pheasant | Male | - | DTP | DTP |
| 2 | SHU2 | Pheasant | Female | - | DTP | DTP |
| 3 | SHU3 | Pheasant | Male | - | DTP | DTP |
| 4 | SHU4 | Pheasant | Female | - | DTP | DTP |
| 5 | SHU5 | Pheasant | Male | - | DTP | DTP |
| 6 | SHU6 | Pheasant | Male | - | DTP | DTP |
| 7 | SHU7 | Pheasant | Female | - | DTP | DTP |
| 8 | SHU8 | Pheasant | Female | - | DTP | DTP |
| 9 | SHU9 | Pheasant | Male | - | DTP | DTP |
| 10 | SHU10 | Pheasant | Male | - | DTP | DTP |
| 11 | SHU11 | Pheasant | Female | - | DTP | DTP |
| 12 | SHU12 | Pheasant | Female | - | DTP | DTP |
| 13 | SHU13 | Pheasant | Female | - | DTP | DTP |
| 14 | SHU14 | Pheasant | Female | - | DTP | DTP |
| 15 | SHU15 | Pheasant | Female | - | DTP | DTP |
| 16 | SHU16 | Pheasant | Female | - | DTP | DTP |
| 17 | SHU17 | Pheasant | Male | - | DTP | DTP |
| 18 | SHU18 | Pheasant | Male | - | DTP | DTP |
| 19 | SHU19 | Pheasant | Female | - | DTP | DTP |
| 20 | SHU20 | Pheasant | Male | - | DTP | DTP |
| 21 | SHU21 | Pheasant | Male | - | DTP | DTP |
| 22 | SHU22 | Pheasant | Female | - | DTP | DTP |
| 23 | SHU23 | Pheasant | Male | - | DTP | DTP |
| 24 | SHU24 | Pheasant | Male | - | DTP | DTP |
| 25 | SHU25 | Pheasant | Male | - | DTP | DTP |
| 26 | SHU26 | Pheasant | Male | - | DTP | DTP |
| 27 | SHU27 | Pheasant | Female | - | DTP | DTP |
| 28 | SHU28 | Pheasant | Male | - | DTP | DTP |
| 29 | SHU29 | Pheasant | Male | - | DTP | DTP |
| 30 | SHU30 | Pheasant | Female | - | DTP | DTP |
| 31 | SHU31 | Pheasant | Male | - | DTP | DTP |
| 32 | SHU32 | Pheasant | Female | - | DTP | DTP |
| 33 | SHU33 | Pheasant | Female | - | DTP | DTP |
| 34 | SHU34 | Pheasant | Female | - | DTP | DTP |
| 35 | SHU35 | Pheasant | Male | - | DTP | DTP |
| 36 | SHU36 | Pheasant | Female | - | DTP | DTP |
| 37 | SHU37 | Pheasant | Male | - | DTP | DTP |
| 38 | SHU38 | Pheasant | Female | - | DTP | DTP |
| 39 | SHU39 | Pheasant | Female | - | DTP | DTP |
| 40 | SHU40 | Pheasant | Male | - | DTP | DTP |
| 41 | SHU41 | Pheasant | Male | - | DTP | DTP |
| 42 | SHU42 | Pheasant | Female | - | DTP | DTP |
| 43 | SHU43 | Pheasant | Female | - | DTP | DTP |
| 44 | SHU44 | Pheasant | Female | - | DTP | DTP |
| 45 | SHU45 | Pheasant | Female | DNPTHA1100130 | DTP | DTP |
| 46 | SHU46 | Pheasant | Male | DNPTHA1200322 | DTP | DTP |
| 47 | SHU47 | Pheasant | Female | 56KP482 | DTP | DTP |
| 48 | SHU48 | Pheasant | Male | DNPTHA1200323 | DTP | DTP |
| 49 | SHU49 | Pheasant | Female | DNPTHA1100512 | DTP | DTP |
| 50 | SHU50 | Pheasant | Male | 55KP2A7 | DTP | DTP |
| 51 | SHU51 | Pheasant | Female | DNPTHA1100076 | DTP | DTP |
| 52 | SHU52 | Pheasant | Male | DNPTHA1200325 | DTP | DTP |
| 53 | SHU53 | Pheasant | Male | - | DTP | DTP |
| 54 | SHU54 | Pheasant | Male | DNPTHA1200268 | DTP | DTP |
| 55 | SHU55 | Pheasant | Male | - | DTP | DTP |
| 56 | SHU56 | Pheasant | Male | DNPTHA1200308 | DTP | DTP |
| 57 | SHU57 | Pheasant | Female | LO4081 | DTP | DTP |
| 58 | SHU58 | Pheasant | Male | DNPTHA1200316 | DTP | DTP |
| 59 | SHU59 | Pheasant | Female | DNPTHA1100516 | DTP | DTP |
| 60 | SHU60 | Pheasant | Male | 56KP453 | DTP | DTP |
| 61 | SHU61 | Pheasant | Male | DNPTHA1200924 | DTP | DTP |
| 62 | SHU62 | Pheasant | Male | DNATHA1200270 | DTP | DTP |
| 63 | SHU63 | Pheasant | Male | THAM04012 | DTP | DTP |
| 64 | SHU64 | Pheasant | Female | DNPTHA1200939 | DTP | DTP |
| 65 | SHU65 | Pheasant | Male | - | DTP | DTP |
| 66 | SHU66 | Pheasant | Female | - | DTP | DTP |
| 67 | SHU67 | Pheasant | Male | DNPTHA1200302 | DTP | DTP |
| 68 | SHU68 | Pheasant | Female | DNPYHA1100100 | DTP | DTP |
| 69 | SHU69 | Pheasant | Male | DNPTHA1100565 | DTP | DTP |
| 70 | SHU70 | Pheasant | Male | DNPTHA1200925 | DTP | DTP |
| 71 | SHU71 | Pheasant | Female | - | DTP | DTP |
| 72 | SHU72 | Pheasant | Male | DNPTHA1200940 | DTP | DTP |
| 73 | SHU73 | Pheasant | Male | 55KP250 | DTP | DTP |
| 74 | SHU74 | Pheasant | Female | 56KP434 | DTP | DTP |
| 75 | SHU75 | Pheasant | Male | 55KP247 | DTP | DTP |
| 76 | SHU76 | Pheasant | Female | 55KP249 | DTP | DTP |
| 77 | SHU77 | Pheasant | Male | DNPTHA1200304 | DTP | DTP |
| 78 | SHU78 | Pheasant | Female | - | DTP | DTP |
| 79 | SHU79 | Pheasant | Male | DNPTHA1200921 | DTP | DTP |
| 80 | SHU80 | Pheasant | Female | 56KP472 | DTP | DTP |
| 81 | SHU81 | Pheasant | Male | 56KP458 | DTP | DTP |
| 82 | SHU82 | Pheasant | Female | - | DTP | DTP |

DTP: Doi Tung Wildlife Breeding Center
