# Supplementary material for: Reduced genetic variability in a captive-bred population of the endangered Hume’s pheasant (Syrmaticus humiae, Hume 1881) revealed by microsatellite genotyping and D-loop sequencing
Source: PLoS One. 2021 Aug 27;16(8):e0256573. doi: 10.1371/journal.pone.0256573 (PMC8396778; doi:10.1371/journal.pone.0256573)
Supplement: S3 Table — (DOCX) [file pone.0256573.s003.docx]

**S3 Table Primers for microsatellite loci and partial mitochondrial D-loop used in the study.**

| Marker | Primer sequence (5′ to 3′) | Repeat motif | *T*_a_ (ºC) | size (bp) |
| --- | --- | --- | --- | --- |
| Shoul-15 | GCAATGAGAAGAGGGCTGAGT | (AC)_19_ | 58 | 134–184 |
|  | GAGTCTTTGCTGTGGGGTCTA |  |  |  |
| ShuI-16 | ATTTCTGTTATTGCAGTGACCTA | (AC)_10_ | 58 | 261–279 |
|  | CCAGTTACAGTACCGGACAGAGA |  |  |  |
| ShuI-22 | CAGAGCGTTGCATTCCAGT | (AC)_17_ | 50 | 262–300 |
|  | TGCAAACACATCTCCATACAAG |  |  |  |
| ShuI-35 | AATGGCAGCAGTTGGGTGTAAGG | (AC)_21_ | 50 | 354–380 |
|  | GGGCTATGCGCATGCTCTGATT |  |  |  |
| ShuI-36 | GCCGGTCTGCTGATGGTTT | (AC)_21_ | 55 | 311–359 |
|  | GCTTTCTTCTTTCCGCCTTCC |  |  |  |
| ShuI-50 | GCTTTCTTCTTTCCGCCTTCC | (GT)_24_ | 55 | 313–363 |
|  | CGGTCTGCTGATGGTTTTGTTAT |  |  |  |
| ShuI-51 | CTATGCGCATGCTCTGATTGGTT | (AC)_13_ | 55 | 332–358 |
|  | GTAAGGCGTTGGAGCGTGTGAG |  |  |  |
| ShuI-52 | CATCTTCCAGCTGAGCGTCTTAG | (AC)_11_ | 55 | 172–184 |
|  | AATATTTTTGCCACTCCTGCTTCT |  |  |  |
| ShuI-54 | ACTTAGCCCTCTGGTTACG | (AC)_16_ | 50 | 366–420 |
|  | GGAAGCCTGAATGAGTGTT |  |  |  |
| ShuII-62 | TGCCATAGTCCGCTCACCAG | (CT)_12_ | 52 | 236–270 |
|  | ATGCCTAACCAAATCTTCTCCTAT |  |  |  |
| ShuII-67 | AGAGCCTAGGCAGTGAAAA | (AC)_20_  (AG)_12_ | 52 | 274–320 |
|  | GCATTAGATCGTGAGCAGTAT |  |  |  |
| ShuI-108 | AGCAGGAGATCAAGTGAAGTGTAT | (AC)_20_ | 50 | 277–327 |
|  | TGCCAAGCTATCCCAAAAC |  |  |  |

Bei Y, Li J, Meng S, Li G, Sun B, 2013. Isolation and characterization of 12 novel microsatellite loci in Hume’s Pheasant, *Syrmaticus humiae*. *Conserv Genet Resour* 5:19–21.
