# Supplementary material for: Reduced genetic variability in a captive-bred population of the endangered Hume’s pheasant (Syrmaticus humiae, Hume 1881) revealed by microsatellite genotyping and D-loop sequencing
Source: PLoS One. 2021 Aug 27;16(8):e0256573. doi: 10.1371/journal.pone.0256573 (PMC8396778; doi:10.1371/journal.pone.0256573)
Supplement: S4 Table — (DOCX) [file pone.0256573.s004.docx]

**S4 Table Summary accession number of mitochondrial D-loop sequences of *Syrmaticus humiae* (Hume, 1881) population.**

| Code | Locality | Accession number |
| --- | --- | --- |
| SHU1 | DTP | LC625540 |
| SHU2 | DTP | LC625541 |
| SHU3 | DTP | LC625542 |
| SHU4 | DTP | LC625543 |
| SHU5 | DTP | LC625544 |
| SHU6 | DTP | LC625545 |
| SHU7 | DTP | LC625546 |
| SHU8 | DTP | LC625547 |
| SHU9 | DTP | LC625548 |
| SHU10 | DTP | LC625549 |
| SHU11 | DTP | LC625550 |
| SHU12 | DTP | LC625551 |
| SHU13 | DTP | LC625552 |
| SHU14 | DTP | LC625553 |
| SHU15 | DTP | LC625554 |
| SHU16 | DTP | LC625555 |
| SHU17 | DTP | LC625556 |
| SHU18 | DTP | LC625557 |
| SHU19 | DTP | LC625558 |
| SHU20 | DTP | LC625605 |
| SHU21 | DTP | LC625559 |
| SHU22 | DTP | LC625560 |
| SHU23 | DTP | LC625607 |
| SHU24 | DTP | LC625604 |
| SHU25 | DTP | LC625561 |
| SHU26 | DTP | LC625621 |
| SHU27 | DTP | LC625562 |
| SHU28 | DTP | LC625563 |
| SHU29 | DTP | LC625564 |
| SHU30 | DTP | LC625565 |
| SHU31 | DTP | LC625603 |
| SHU32 | DTP | LC625566 |
| SHU33 | DTP | LC625567 |
| SHU34 | DTP | LC625568 |
| SHU35 | DTP | LC625569 |
| SHU36 | DTP | LC625608 |
| SHU37 | DTP | LC625570 |
| SHU38 | DTP | LC625571 |
| SHU39 | DTP | LC625572 |
| SHU40 | DTP | LC625573 |
| SHU41 | DTP | LC625574 |
| SHU42 | DTP | LC625575 |
| SHU43 | DTP | LC625576 |
| SHU44 | DTP | LC625577 |
| SHU45 | DTP | LC625578 |
| SHU46 | DTP | LC625579 |
| SHU47 | DTP | LC625580 |
| SHU48 | DTP | LC625581 |
| SHU49 | DTP | LC625582 |
| SHU50 | DTP | LC625583 |
| SHU51 | DTP | LC625584 |
| SHU52 | DTP | LC625585 |
| SHU53 | DTP | LC625586 |
| SHU54 | DTP | LC625610 |
| SHU55 | DTP | LC625587 |
| SHU56 | DTP | LC625588 |
| SHU57 | DTP | LC625611 |
| SHU58 | DTP | LC625589 |
| SHU59 | DTP | LC625616 |
| SHU60 | DTP | LC625617 |
| SHU61 | DTP | LC625606 |
| SHU62 | DTP | LC625618 |
| SHU63 | DTP | LC625590 |
| SHU64 | DTP | LC625609 |
| SHU65 | DTP | LC625591 |
| SHU66 | DTP | LC625592 |
| SHU67 | DTP | LC625620 |
| SHU68 | DTP | LC625619 |
| SHU69 | DTP | LC625593 |
| SHU70 | DTP | LC625612 |
| SHU71 | DTP | LC625594 |
| SHU72 | DTP | LC625595 |
| SHU73 | DTP | LC625596 |
| SHU74 | DTP | LC625615 |
| SHU75 | DTP | LC625613 |
| SHU76 | DTP | LC625597 |
| SHU77 | DTP | LC625598 |
| SHU78 | DTP | LC625614 |
| SHU79 | DTP | LC625599 |
| SHU80 | DTP | LC625600 |
| SHU81 | DTP | LC625601 |
| SHU82 | DTP | LC625602 |

DTP: Doi Tung Wildlife Breeding Cente
