# Supplementary material for: Reduced genetic variability in a captive-bred population of the endangered Hume’s pheasant (Syrmaticus humiae, Hume 1881) revealed by microsatellite genotyping and D-loop sequencing
Source: PLoS One. 2021 Aug 27;16(8):e0256573. doi: 10.1371/journal.pone.0256573 (PMC8396778; doi:10.1371/journal.pone.0256573)
Supplement: S5 Table — Detailed information for all individuals is presented in S2 Table. (DOCX) [file pone.0256573.s005.docx]

**S5 Table Genetic diversity of 82 Hume’s pheasant (*Syrmaticus humiae,* Hume 1881) individuals based on 12 microsatellite loci.** Detailed information for all individuals is presented in S2 Table.

| Pop | Locus | *N* | *N*_a_ | AR | *N*_e_ | *I* | *H*_o_ | *H*_e_ | *M* ratio | PIC | *F* | *p*-value |
| --- | --- | --- | --- | --- | --- | --- | --- | --- | --- | --- | --- | --- |
| DTP | shul35 | 82 | 7.000 | 7.000 | 1.207 | 0.432 | 0.073 | 0.172 | 0.044 | 0.167 | 0.574 | 0.000 |
|  | shul52 | 82 | 4.000 | 4.000 | 1.050 | 0.140 | 0.024 | 0.048 | 0.025 | 0.048 | 0.491 | 0.000 |
|  | shul36 | 82 | 12.000 | 12.000 | 3.296 | 1.672 | 0.024 | 0.697 | 0.078 | 0.674 | 0.965 | 0.000 |
|  | shul54 | 82 | 4.000 | 4.000 | 1.615 | 0.719 | 0.000 | 0.381 | 0.025 | 0.346 | 1.000 | 0.000 |
|  | shull67 | 82 | 9.000 | 9.000 | 4.554 | 1.709 | 0.012 | 0.780 | 0.058 | 0.749 | 0.984 | 0.000 |
|  | shull108 | 82 | 6.000 | 6.000 | 1.574 | 0.737 | 0.000 | 0.365 | 0.038 | 0.333 | 1.000 | 0.000 |
|  | shul50 | 82 | 10.000 | 10.000 | 3.116 | 1.549 | 0.024 | 0.679 | 0.065 | 0.649 | 0.964 | 0.000 |
|  | shul51 | 82 | 6.000 | 6.000 | 1.224 | 0.456 | 0.012 | 0.183 | 0.038 | 0.178 | 0.933 | 0.000 |
|  | shul62 | 82 | 11.000 | 11.000 | 2.291 | 1.278 | 0.037 | 0.564 | 0.071 | 0.535 | 0.935 | 0.000 |
|  | shoul15 | 82 | 9.000 | 9.000 | 3.296 | 1.502 | 0.122 | 0.697 | 0.058 | 0.659 | 0.825 | 0.000 |
|  | shuI16 | 82 | 8.000 | 8.000 | 1.271 | 0.522 | 0.085 | 0.213 | 0.051 | 0.206 | 0.600 | 0.000 |
|  | shuI22 | 82 | 4.000 | 4.000 | 1.791 | 0.795 | 0.049 | 0.442 | 0.025 | 0.391 | 0.890 | 0.000 |
|  | Mean | 82 | 7.500 | 7.500 | 2.190 | 0.959 | 0.039 | 0.435 | 0.048 | 0.411 | 0.847 | 0.000 |
|  | SD | 0 | 0.802 | 0.802 | 0.324 | 0.159 | 0.011 | 0.071 | 0.019 | 0.237 | 0.053 | 0.000 |
