# Supplementary material for: Reduced genetic variability in a captive-bred population of the endangered Hume’s pheasant (Syrmaticus humiae, Hume 1881) revealed by microsatellite genotyping and D-loop sequencing
Source: PLoS One. 2021 Aug 27;16(8):e0256573. doi: 10.1371/journal.pone.0256573 (PMC8396778; doi:10.1371/journal.pone.0256573)
Supplement: S6 Table — Numbers indicate p-values with 110 permutations. (DOCX) [file pone.0256573.s006.docx]

**S6 Table Pairwise differentiation of linkage disequilibrium of Hume’s pheasant (*Syrmaticus humiae,* Hume 1881) individuals at the Doi Tung Wildlife Breeding Center based on 12 microsatellite loci.** Numbers indicate *p*-values with 110 permutations.

| Locus | SHUL51 | SHUL52 | SHUL36 | SHUL50 | SHUL35 | SHUL16 | SHUL22 | STUL67 | SHUL62 | SHUL108 | SHUL54 | SHUL15 |
| --- | --- | --- | --- | --- | --- | --- | --- | --- | --- | --- | --- | --- |
| SHUL51 | 0.000 |  |  |  |  |  |  |  |  |  |  |  |
| SHUL52 | 0.890 | 0.000 |  |  |  |  |  |  |  |  |  |  |
| SHUL36 | 0.860 | 0.380 | 0.0000 |  |  |  |  |  |  |  |  |  |
| SHUL50 | 0.840 | 0.720 | 0.000 | 0.000 |  |  |  |  |  |  |  |  |
| SHUL35 | 0.490 | 0.050 | 0.390 | 0.470 | 0.000 |  |  |  |  |  |  |  |
| SHUL16 | 0.082 | 0.950 | 0.960 | 0.970 | 0.200 | 0.000 |  |  |  |  |  |  |
| SHUL22 | 0.740 | 0.970 | 0.420 | 0.120 | 0.120 | 0.028 | 0.000 |  |  |  |  |  |
| STUL67 | 0.910 | 0.440 | 0.240 | 0.700 | 0.300 | 0.720 | 0.570 | 0.000 |  |  |  |  |
| SHUL62 | 0.610 | 0.950 | 0.130 | 0.670 | 0.600 | 0.570 | 0.700 | 0.520 | 0.000 |  |  |  |
| SHUL108 | 0.380 | 0.920 | 0.660 | 0.760 | 0.480 | 0.780 | 0.470 | 0.800 | 0.820 | 0.000 |  |  |
| SHUL54 | 0.630 | 0.930 | 0.470 | 0.940 | 0.140 | 0.830 | 0.430 | 0.490 | 0.710 | 0.810 | 0.000 |  |
| SHUL15 | 0.570 | 0.680 | 0.810 | 0.540 | 0.680 | 0.550 | 0.960 | 0.980 | 0.930 | 0.750 | 0.000 | 0.000 |
