# Supplementary material for: Reduced genetic variability in a captive-bred population of the endangered Hume’s pheasant (Syrmaticus humiae, Hume 1881) revealed by microsatellite genotyping and D-loop sequencing
Source: PLoS One. 2021 Aug 27;16(8):e0256573. doi: 10.1371/journal.pone.0256573 (PMC8396778; doi:10.1371/journal.pone.0256573)
Supplement: S7 Table — Data were calculated using Bottleneck version 1.2.02. (Cornuet and Luikart, 1996). Detailed information for all individuals is presented in S2 Table. (DOCX) [file pone.0256573.s007.docx]

**S7 Table Observed and expected heterozygosity of Hume’s pheasant (*Syrmaticus humiae,* Hume 1881) based on 12 microsatellite loci at the Doi Tung Wildlife Breeding Center and genetic bottlenecks for all individuals.** Data were calculated using Bottleneck version 1.2.02. (Cornuet and Luikart, 1996). Detailed information for all individuals is presented in S2 Table.

| Species | Captivity/wild | | *H*_o_ | *H*_e_ | *p* value | Wilcoxon test | | Mode-shift test | *M* ratio |
| --- | --- | --- | --- | --- | --- | --- | --- | --- | --- |
|  |  |  |  |  |  | T.P.M. | S.M.M. |  |  |
| *Syrmaticus humiae* | | DTP | 0.039 ± 0.011 | 0.435 ± 0.071 | < 0.05 | 1.000 | 1.000 | normal L-shaped distribution | 0.048 ± 0.019 |

DTP: Doi Tung Wildlife Breeding Center

T.P.M: Two - Phased Modes

S.M.M:The Step - Wise Mutation Model

Cornuet JM, Luikart G, 1996. Description and power analysis of two tests for detecting recent population bottlenecks from allele frequency data. *Genetics* 144:2001–2014.
