# Supplementary material for: Reduced genetic variability in a captive-bred population of the endangered Hume’s pheasant (Syrmaticus humiae, Hume 1881) revealed by microsatellite genotyping and D-loop sequencing
Source: PLoS One. 2021 Aug 27;16(8):e0256573. doi: 10.1371/journal.pone.0256573 (PMC8396778; doi:10.1371/journal.pone.0256573)
Supplement: S8 Table — Detailed information for all individuals is presented in S1 Table. (DOCX) [file pone.0256573.s008.docx]

**S8 Table. Pairwise genetic relatedness (*r*) for all 82 *Syrmaticus humiae* (Hume, 1881) individuals.** Detailed information for all individuals is presented in S1 Table.

| **Sample 1** | **Sample 2** | **LRM** |
| --- | --- | --- |
| SHU7 | SHU54 | -0.258 |
| SHU7 | SHU57 | -0.253 |
| SHU2 | SHU57 | -0.239 |
| SHU7 | SHU40 | -0.223 |
| SHU40 | SHU63 | -0.221 |
| SHU7 | SHU70 | -0.219 |
| SHU7 | SHU73 | -0.219 |
| SHU40 | SHU58 | -0.217 |
| SHU18 | SHU40 | -0.211 |
| SHU40 | SHU48 | -0.211 |
| SHU2 | SHU40 | -0.210 |
| SHU2 | SHU70 | -0.206 |
| SHU2 | SHU73 | -0.206 |
| SHU2 | SHU4 | -0.205 |
| SHU2 | SHU54 | -0.204 |
| SHU7 | SHU28 | -0.201 |
| SHU57 | SHU63 | -0.200 |
| SHU57 | SHU58 | -0.196 |
| SHU18 | SHU57 | -0.191 |
| SHU48 | SHU57 | -0.191 |
| SHU2 | SHU28 | -0.189 |
| SHU16 | SHU40 | -0.185 |
| SHU54 | SHU69 | -0.182 |
| SHU27 | SHU54 | -0.179 |
| SHU7 | SHU64 | -0.177 |
| SHU7 | SHU15 | -0.176 |
| SHU16 | SHU57 | -0.174 |
| SHU7 | SHU23 | -0.173 |
| SHU63 | SHU73 | -0.172 |
| SHU2 | SHU60 | -0.169 |
| SHU58 | SHU73 | -0.169 |
| SHU58 | SHU70 | -0.169 |
| SHU4 | SHU16 | -0.169 |
| SHU4 | SHU18 | -0.168 |
| SHU4 | SHU48 | -0.168 |
| SHU27 | SHU40 | -0.168 |
| SHU2 | SHU64 | -0.168 |
| SHU54 | SHU58 | -0.167 |
| SHU2 | SHU15 | -0.166 |
| SHU10 | SHU54 | -0.164 |
| SHU10 | SHU23 | -0.164 |
| SHU2 | SHU11 | -0.164 |
| SHU18 | SHU73 | -0.164 |
| SHU48 | SHU73 | -0.164 |
| SHU7 | SHU65 | -0.161 |
| SHU21 | SHU23 | -0.161 |
| SHU28 | SHU63 | -0.159 |
| SHU4 | SHU14 | -0.158 |
| SHU7 | SHU38 | -0.158 |
| SHU7 | SHU44 | -0.155 |
| SHU27 | SHU57 | -0.153 |
| SHU21 | SHU40 | -0.153 |
| SHU14 | SHU40 | -0.152 |
| SHU16 | SHU73 | -0.152 |
| SHU41 | SHU63 | -0.152 |
| SHU18 | SHU28 | -0.152 |
| SHU28 | SHU48 | -0.152 |
| SHU7 | SHU19 | -0.151 |
| SHU23 | SHU69 | -0.151 |
| SHU8 | SHU15 | -0.150 |
| SHU41 | SHU58 | -0.150 |
| SHU11 | SHU76 | -0.149 |
| SHU13 | SHU21 | -0.149 |
| SHU1 | SHU8 | -0.148 |
| SHU29 | SHU54 | -0.147 |
| SHU21 | SHU57 | -0.146 |
| SHU7 | SHU66 | -0.146 |
| SHU18 | SHU41 | -0.145 |
| SHU41 | SHU48 | -0.145 |
| SHU16 | SHU28 | -0.145 |
| SHU23 | SHU52 | -0.145 |
| SHU7 | SHU68 | -0.144 |
| SHU2 | SHU19 | -0.143 |
| SHU35 | SHU40 | -0.143 |
| SHU13 | SHU35 | -0.143 |
| SHU23 | SHU63 | -0.142 |
| SHU4 | SHU69 | -0.141 |
| SHU11 | SHU58 | -0.141 |
| SHU63 | SHU64 | -0.141 |
| SHU58 | SHU60 | -0.139 |
| SHU12 | SHU52 | -0.139 |
| SHU12 | SHU29 | -0.139 |
| SHU15 | SHU63 | -0.138 |
| SHU29 | SHU40 | -0.138 |
| SHU10 | SHU38 | -0.138 |
| SHU13 | SHU29 | -0.137 |
| SHU2 | SHU66 | -0.137 |
| SHU2 | SHU67 | -0.137 |
| SHU15 | SHU58 | -0.136 |
| SHU13 | SHU15 | -0.136 |
| SHU18 | SHU60 | -0.135 |
| SHU48 | SHU60 | -0.135 |
| SHU40 | SHU69 | -0.135 |
| SHU21 | SHU38 | -0.135 |
| SHU18 | SHU64 | -0.135 |
| SHU48 | SHU64 | -0.135 |
| SHU16 | SHU64 | -0.134 |
| SHU17 | SHU40 | -0.134 |
| SHU24 | SHU40 | -0.134 |
| SHU69 | SHU73 | -0.133 |
| SHU69 | SHU70 | -0.133 |
| SHU16 | SHU60 | -0.132 |
| SHU15 | SHU18 | -0.132 |
| SHU15 | SHU48 | -0.132 |
| SHU4 | SHU31 | -0.132 |
| SHU2 | SHU65 | -0.132 |
| SHU27 | SHU73 | -0.131 |
| SHU38 | SHU69 | -0.131 |
| SHU1 | SHU7 | -0.131 |
| SHU27 | SHU70 | -0.131 |
| SHU16 | SHU19 | -0.129 |
| SHU35 | SHU54 | -0.128 |
| SHU13 | SHU27 | -0.128 |
| SHU14 | SHU57 | -0.127 |
| SHU63 | SHU65 | -0.127 |
| SHU38 | SHU63 | -0.127 |
| SHU21 | SHU73 | -0.127 |
| SHU14 | SHU19 | -0.127 |
| SHU12 | SHU27 | -0.126 |
| SHU12 | SHU64 | -0.126 |
| SHU17 | SHU41 | -0.125 |
| SHU24 | SHU41 | -0.125 |
| SHU1 | SHU2 | -0.124 |
| SHU40 | SHU43 | -0.124 |
| SHU44 | SHU69 | -0.124 |
| SHU17 | SHU57 | -0.124 |
| SHU24 | SHU57 | -0.124 |
| SHU21 | SHU44 | -0.124 |
| SHU5 | SHU8 | -0.123 |
| SHU44 | SHU63 | -0.123 |
| SHU19 | SHU63 | -0.122 |
| SHU21 | SHU28 | -0.122 |
| SHU8 | SHU64 | -0.121 |
| SHU16 | SHU42 | -0.121 |
| SHU19 | SHU58 | -0.121 |
| SHU19 | SHU43 | -0.120 |
| SHU12 | SHU57 | -0.119 |
| SHU16 | SHU23 | -0.119 |
| SHU17 | SHU19 | -0.119 |
| SHU19 | SHU24 | -0.119 |
| SHU11 | SHU53 | -0.119 |
| SHU38 | SHU52 | -0.119 |
| SHU17 | SHU60 | -0.118 |
| SHU24 | SHU60 | -0.118 |
| SHU35 | SHU73 | -0.118 |
| SHU9 | SHU63 | -0.118 |
| SHU10 | SHU57 | -0.118 |
| SHU11 | SHU69 | -0.118 |
| SHU4 | SHU34 | -0.118 |
| SHU43 | SHU57 | -0.118 |
| SHU35 | SHU70 | -0.118 |
| SHU18 | SHU19 | -0.118 |
| SHU19 | SHU48 | -0.118 |
| SHU34 | SHU64 | -0.118 |
| SHU53 | SHU54 | -0.118 |
| SHU31 | SHU40 | -0.117 |
| SHU8 | SHU21 | -0.117 |
| SHU10 | SHU68 | -0.117 |
| SHU19 | SHU76 | -0.117 |
| SHU14 | SHU42 | -0.117 |
| SHU27 | SHU41 | -0.117 |
| SHU2 | SHU23 | -0.117 |
| SHU8 | SHU57 | -0.116 |
| SHU9 | SHU27 | -0.116 |
| SHU9 | SHU21 | -0.116 |
| SHU10 | SHU65 | -0.116 |
| SHU15 | SHU17 | -0.115 |
| SHU15 | SHU24 | -0.115 |
| SHU4 | SHU76 | -0.115 |
| SHU42 | SHU58 | -0.115 |
| SHU68 | SHU69 | -0.115 |
| SHU10 | SHU19 | -0.115 |
| SHU63 | SHU66 | -0.115 |
| SHU16 | SHU79 | -0.115 |
| SHU13 | SHU14 | -0.114 |
| SHU58 | SHU67 | -0.114 |
| SHU63 | SHU68 | -0.114 |
| SHU21 | SHU64 | -0.114 |
| SHU7 | SHU26 | -0.113 |
| SHU29 | SHU70 | -0.113 |
| SHU21 | SHU65 | -0.113 |
| SHU27 | SHU68 | -0.113 |
| SHU8 | SHU35 | -0.113 |
| SHU43 | SHU60 | -0.113 |
| SHU58 | SHU66 | -0.113 |
| SHU43 | SHU54 | -0.113 |
| SHU17 | SHU23 | -0.113 |
| SHU23 | SHU24 | -0.113 |
| SHU54 | SHU76 | -0.112 |
| SHU16 | SHU66 | -0.112 |
| SHU40 | SHU72 | -0.112 |
| SHU18 | SHU42 | -0.112 |
| SHU42 | SHU48 | -0.112 |
| SHU19 | SHU21 | -0.112 |
| SHU8 | SHU52 | -0.112 |
| SHU17 | SHU42 | -0.111 |
| SHU24 | SHU42 | -0.111 |
| SHU27 | SHU65 | -0.111 |
| SHU42 | SHU43 | -0.111 |
| SHU16 | SHU65 | -0.111 |
| SHU58 | SHU79 | -0.110 |
| SHU60 | SHU69 | -0.110 |
| SHU10 | SHU15 | -0.110 |
| SHU13 | SHU63 | -0.110 |
| SHU4 | SHU7 | -0.110 |
| SHU4 | SHU20 | -0.110 |
| SHU43 | SHU67 | -0.110 |
| SHU18 | SHU66 | -0.110 |
| SHU48 | SHU66 | -0.110 |
| SHU29 | SHU57 | -0.109 |
| SHU58 | SHU65 | -0.109 |
| SHU9 | SHU35 | -0.108 |
| SHU14 | SHU79 | -0.108 |
| SHU16 | SHU41 | -0.108 |
| SHU8 | SHU29 | -0.108 |
| SHU21 | SHU39 | -0.108 |
| SHU16 | SHU71 | -0.108 |
| SHU7 | SHU32 | -0.108 |
| SHU18 | SHU79 | -0.107 |
| SHU48 | SHU79 | -0.107 |
| SHU2 | SHU26 | -0.107 |
| SHU41 | SHU43 | -0.107 |
| SHU14 | SHU28 | -0.107 |
| SHU13 | SHU47 | -0.106 |
| SHU41 | SHU72 | -0.106 |
| SHU12 | SHU63 | -0.106 |
| SHU15 | SHU27 | -0.106 |
| SHU2 | SHU38 | -0.106 |
| SHU16 | SHU62 | -0.106 |
| SHU11 | SHU64 | -0.106 |
| SHU12 | SHU66 | -0.106 |
| SHU23 | SHU33 | -0.105 |
| SHU18 | SHU65 | -0.105 |
| SHU48 | SHU65 | -0.105 |
| SHU1 | SHU63 | -0.105 |
| SHU40 | SHU52 | -0.105 |
| SHU11 | SHU74 | -0.105 |
| SHU35 | SHU71 | -0.105 |
| SHU57 | SHU72 | -0.105 |
| SHU29 | SHU41 | -0.105 |
| SHU17 | SHU73 | -0.105 |
| SHU24 | SHU73 | -0.105 |
| SHU8 | SHU20 | -0.105 |
| SHU2 | SHU44 | -0.105 |
| SHU25 | SHU29 | -0.105 |
| SHU19 | SHU72 | -0.105 |
| SHU17 | SHU79 | -0.104 |
| SHU24 | SHU79 | -0.104 |
| SHU9 | SHU29 | -0.104 |
| SHU27 | SHU39 | -0.104 |
| SHU12 | SHU55 | -0.104 |
| SHU12 | SHU78 | -0.104 |
| SHU35 | SHU60 | -0.103 |
| SHU53 | SHU70 | -0.103 |
| SHU29 | SHU68 | -0.103 |
| SHU25 | SHU52 | -0.103 |
| SHU12 | SHU73 | -0.103 |
| SHU14 | SHU65 | -0.103 |
| SHU43 | SHU79 | -0.103 |
| SHU12 | SHU53 | -0.103 |
| SHU16 | SHU38 | -0.103 |
| SHU14 | SHU45 | -0.102 |
| SHU4 | SHU72 | -0.102 |
| SHU13 | SHU53 | -0.102 |
| SHU58 | SHU71 | -0.102 |
| SHU14 | SHU71 | -0.102 |
| SHU12 | SHU70 | -0.102 |
| SHU12 | SHU40 | -0.102 |
| SHU29 | SHU65 | -0.102 |
| SHU2 | SHU32 | -0.102 |
| SHU19 | SHU29 | -0.101 |
| SHU39 | SHU63 | -0.101 |
| SHU8 | SHU66 | -0.101 |
| SHU1 | SHU18 | -0.101 |
| SHU1 | SHU48 | -0.101 |
| SHU6 | SHU12 | -0.101 |
| SHU11 | SHU59 | -0.101 |
| SHU35 | SHU39 | -0.101 |
| SHU25 | SHU27 | -0.101 |
| SHU17 | SHU66 | -0.101 |
| SHU24 | SHU66 | -0.101 |
| SHU11 | SHU40 | -0.101 |
| SHU10 | SHU40 | -0.101 |
| SHU58 | SHU62 | -0.101 |
| SHU31 | SHU70 | -0.100 |
| SHU60 | SHU72 | -0.100 |
| SHU43 | SHU70 | -0.100 |
| SHU23 | SHU72 | -0.100 |
| SHU10 | SHU73 | -0.100 |
| SHU31 | SHU57 | -0.100 |
| SHU17 | SHU65 | -0.100 |
| SHU24 | SHU65 | -0.100 |
| SHU53 | SHU57 | -0.100 |
| SHU41 | SHU52 | -0.100 |
| SHU14 | SHU62 | -0.100 |
| SHU10 | SHU70 | -0.100 |
| SHU14 | SHU41 | -0.100 |
| SHU10 | SHU61 | -0.100 |
| SHU18 | SHU71 | -0.100 |
| SHU48 | SHU71 | -0.100 |
| SHU13 | SHU31 | -0.099 |
| SHU11 | SHU57 | -0.099 |
| SHU2 | SHU51 | -0.099 |
| SHU13 | SHU66 | -0.099 |
| SHU8 | SHU40 | -0.099 |
| SHU9 | SHU52 | -0.099 |
| SHU2 | SHU50 | -0.099 |
| SHU12 | SHU75 | -0.098 |
| SHU8 | SHU73 | -0.098 |
| SHU52 | SHU68 | -0.098 |
| SHU6 | SHU23 | -0.098 |
| SHU34 | SHU66 | -0.098 |
| SHU35 | SHU42 | -0.098 |
| SHU8 | SHU70 | -0.098 |
| SHU43 | SHU65 | -0.098 |
| SHU18 | SHU62 | -0.098 |
| SHU48 | SHU62 | -0.098 |
| SHU13 | SHU16 | -0.098 |
| SHU15 | SHU72 | -0.098 |
| SHU20 | SHU42 | -0.098 |
| SHU43 | SHU66 | -0.098 |
| SHU19 | SHU27 | -0.097 |
| SHU18 | SHU23 | -0.097 |
| SHU23 | SHU48 | -0.097 |
| SHU29 | SHU39 | -0.097 |
| SHU21 | SHU61 | -0.097 |
| SHU7 | SHU61 | -0.097 |
| SHU35 | SHU67 | -0.097 |
| SHU16 | SHU44 | -0.097 |
| SHU35 | SHU55 | -0.097 |
| SHU42 | SHU72 | -0.096 |
| SHU8 | SHU54 | -0.096 |
| SHU10 | SHU66 | -0.096 |
| SHU14 | SHU60 | -0.096 |
| SHU16 | SHU45 | -0.096 |
| SHU41 | SHU53 | -0.096 |
| SHU8 | SHU78 | -0.096 |
| SHU51 | SHU69 | -0.096 |
| SHU2 | SHU68 | -0.096 |
| SHU20 | SHU45 | -0.095 |
| SHU31 | SHU42 | -0.095 |
| SHU43 | SHU45 | -0.095 |
| SHU50 | SHU69 | -0.095 |
| SHU34 | SHU73 | -0.095 |
| SHU17 | SHU38 | -0.095 |
| SHU24 | SHU38 | -0.095 |
| SHU16 | SHU32 | -0.095 |
| SHU12 | SHU19 | -0.095 |
| SHU13 | SHU64 | -0.095 |
| SHU52 | SHU60 | -0.095 |
| SHU19 | SHU53 | -0.095 |
| SHU34 | SHU70 | -0.095 |
| SHU34 | SHU40 | -0.094 |
| SHU14 | SHU44 | -0.094 |
| SHU13 | SHU54 | -0.094 |
| SHU21 | SHU66 | -0.094 |
| SHU47 | SHU76 | -0.093 |
| SHU51 | SHU52 | -0.093 |
| SHU14 | SHU32 | -0.093 |
| SHU28 | SHU34 | -0.093 |
| SHU12 | SHU17 | -0.093 |
| SHU12 | SHU24 | -0.093 |
| SHU39 | SHU52 | -0.093 |
| SHU70 | SHU76 | -0.093 |
| SHU50 | SHU52 | -0.092 |
| SHU40 | SHU76 | -0.092 |
| SHU67 | SHU69 | -0.092 |
| SHU65 | SHU76 | -0.092 |
| SHU13 | SHU19 | -0.092 |
| SHU42 | SHU52 | -0.092 |
| SHU21 | SHU74 | -0.092 |
| SHU9 | SHU16 | -0.092 |
| SHU8 | SHU75 | -0.091 |
| SHU35 | SHU61 | -0.091 |
| SHU35 | SHU79 | -0.091 |
| SHU21 | SHU41 | -0.090 |
| SHU57 | SHU76 | -0.090 |
| SHU17 | SHU45 | -0.090 |
| SHU24 | SHU45 | -0.090 |
| SHU12 | SHU60 | -0.090 |
| SHU4 | SHU33 | -0.090 |
| SHU51 | SHU76 | -0.090 |
| SHU16 | SHU55 | -0.090 |
| SHU17 | SHU51 | -0.090 |
| SHU24 | SHU51 | -0.090 |
| SHU72 | SHU79 | -0.089 |
| SHU50 | SHU76 | -0.089 |
| SHU13 | SHU57 | -0.089 |
| SHU4 | SHU63 | -0.089 |
| SHU17 | SHU50 | -0.089 |
| SHU24 | SHU50 | -0.089 |
| SHU17 | SHU64 | -0.089 |
| SHU24 | SHU64 | -0.089 |
| SHU26 | SHU58 | -0.088 |
| SHU20 | SHU40 | -0.088 |
| SHU61 | SHU69 | -0.088 |
| SHU25 | SHU63 | -0.088 |
| SHU4 | SHU15 | -0.088 |
| SHU8 | SHU55 | -0.088 |
| SHU8 | SHU47 | -0.088 |
| SHU21 | SHU68 | -0.088 |
| SHU32 | SHU43 | -0.088 |
| SHU27 | SHU66 | -0.088 |
| SHU9 | SHU14 | -0.087 |
| SHU55 | SHU69 | -0.087 |
| SHU35 | SHU74 | -0.087 |
| SHU29 | SHU61 | -0.087 |
| SHU11 | SHU66 | -0.087 |
| SHU27 | SHU61 | -0.087 |
| SHU23 | SHU62 | -0.087 |
| SHU17 | SHU32 | -0.087 |
| SHU24 | SHU32 | -0.087 |
| SHU42 | SHU53 | -0.087 |
| SHU13 | SHU20 | -0.087 |
| SHU23 | SHU34 | -0.087 |
| SHU20 | SHU79 | -0.087 |
| SHU31 | SHU79 | -0.087 |
| SHU18 | SHU38 | -0.086 |
| SHU38 | SHU48 | -0.086 |
| SHU12 | SHU67 | -0.086 |
| SHU60 | SHU76 | -0.086 |
| SHU23 | SHU40 | -0.086 |
| SHU31 | SHU45 | -0.086 |
| SHU32 | SHU63 | -0.085 |
| SHU66 | SHU72 | -0.085 |
| SHU65 | SHU72 | -0.085 |
| SHU7 | SHU60 | -0.085 |
| SHU13 | SHU60 | -0.085 |
| SHU10 | SHU64 | -0.085 |
| SHU53 | SHU65 | -0.085 |
| SHU52 | SHU61 | -0.085 |
| SHU52 | SHU79 | -0.085 |
| SHU32 | SHU76 | -0.085 |
| SHU35 | SHU41 | -0.085 |
| SHU11 | SHU73 | -0.084 |
| SHU18 | SHU44 | -0.084 |
| SHU44 | SHU48 | -0.084 |
| SHU1 | SHU69 | -0.084 |
| SHU32 | SHU58 | -0.084 |
| SHU12 | SHU72 | -0.084 |
| SHU10 | SHU26 | -0.084 |
| SHU28 | SHU31 | -0.084 |
| SHU28 | SHU72 | -0.084 |
| SHU6 | SHU8 | -0.084 |
| SHU53 | SHU71 | -0.084 |
| SHU7 | SHU36 | -0.084 |
| SHU9 | SHU58 | -0.084 |
| SHU26 | SHU76 | -0.084 |
| SHU35 | SHU62 | -0.084 |
| SHU15 | SHU76 | -0.084 |
| SHU7 | SHU81 | -0.084 |
| SHU21 | SHU45 | -0.083 |
| SHU52 | SHU73 | -0.083 |
| SHU10 | SHU32 | -0.083 |
| SHU29 | SHU74 | -0.083 |
| SHU8 | SHU14 | -0.083 |
| SHU9 | SHU17 | -0.083 |
| SHU9 | SHU24 | -0.083 |
| SHU17 | SHU47 | -0.083 |
| SHU24 | SHU47 | -0.083 |
| SHU38 | SHU72 | -0.083 |
| SHU63 | SHU71 | -0.083 |
| SHU13 | SHU67 | -0.083 |
| SHU34 | SHU52 | -0.083 |
| SHU2 | SHU55 | -0.083 |
| SHU9 | SHU43 | -0.082 |
| SHU35 | SHU68 | -0.082 |
| SHU11 | SHU19 | -0.082 |
| SHU50 | SHU58 | -0.082 |
| SHU8 | SHU60 | -0.082 |
| SHU21 | SHU25 | -0.082 |
| SHU18 | SHU32 | -0.082 |
| SHU32 | SHU48 | -0.082 |
| SHU31 | SHU54 | -0.082 |
| SHU31 | SHU71 | -0.082 |
| SHU47 | SHU57 | -0.082 |
| SHU11 | SHU75 | -0.082 |
| SHU8 | SHU27 | -0.081 |
| SHU9 | SHU18 | -0.081 |
| SHU9 | SHU48 | -0.081 |
| SHU31 | SHU65 | -0.081 |
| SHU17 | SHU68 | -0.081 |
| SHU24 | SHU68 | -0.081 |
| SHU1 | SHU45 | -0.081 |
| SHU34 | SHU67 | -0.081 |
| SHU23 | SHU74 | -0.080 |
| SHU18 | SHU51 | -0.080 |
| SHU48 | SHU51 | -0.080 |
| SHU21 | SHU32 | -0.080 |
| SHU16 | SHU39 | -0.080 |
| SHU14 | SHU39 | -0.080 |
| SHU53 | SHU79 | -0.080 |
| SHU4 | SHU25 | -0.080 |
| SHU5 | SHU19 | -0.080 |
| SHU12 | SHU54 | -0.080 |
| SHU18 | SHU50 | -0.080 |
| SHU48 | SHU50 | -0.080 |
| SHU33 | SHU38 | -0.080 |
| SHU23 | SHU37 | -0.080 |
| SHU20 | SHU65 | -0.079 |
| SHU29 | SHU34 | -0.079 |
| SHU45 | SHU72 | -0.079 |
| SHU31 | SHU62 | -0.079 |
| SHU35 | SHU45 | -0.079 |
| SHU58 | SHU68 | -0.079 |
| SHU2 | SHU81 | -0.079 |
| SHU36 | SHU69 | -0.079 |
| SHU9 | SHU53 | -0.079 |
| SHU29 | SHU36 | -0.078 |
| SHU15 | SHU74 | -0.078 |
| SHU31 | SHU41 | -0.078 |
| SHU16 | SHU81 | -0.078 |
| SHU50 | SHU53 | -0.078 |
| SHU14 | SHU81 | -0.078 |
| SHU1 | SHU25 | -0.078 |
| SHU4 | SHU19 | -0.078 |
| SHU20 | SHU57 | -0.078 |
| SHU51 | SHU72 | -0.078 |
| SHU25 | SHU35 | -0.078 |
| SHU68 | SHU76 | -0.078 |
| SHU43 | SHU50 | -0.078 |
| SHU10 | SHU14 | -0.078 |
| SHU53 | SHU68 | -0.078 |
| SHU27 | SHU36 | -0.077 |
| SHU11 | SHU65 | -0.077 |
| SHU8 | SHU19 | -0.077 |
| SHU19 | SHU74 | -0.077 |
| SHU61 | SHU63 | -0.077 |
| SHU50 | SHU72 | -0.077 |
| SHU21 | SHU80 | -0.077 |
| SHU20 | SHU32 | -0.077 |
| SHU40 | SHU46 | -0.077 |
| SHU7 | SHU51 | -0.077 |
| SHU18 | SHU68 | -0.077 |
| SHU48 | SHU68 | -0.077 |
| SHU20 | SHU54 | -0.077 |
| SHU45 | SHU63 | -0.077 |
| SHU20 | SHU69 | -0.076 |
| SHU7 | SHU50 | -0.076 |
| SHU20 | SHU70 | -0.076 |
| SHU20 | SHU41 | -0.076 |
| SHU12 | SHU65 | -0.076 |
| SHU1 | SHU67 | -0.076 |
| SHU25 | SHU53 | -0.076 |
| SHU64 | SHU72 | -0.076 |
| SHU46 | SHU57 | -0.076 |
| SHU45 | SHU52 | -0.076 |
| SHU5 | SHU7 | -0.076 |
| SHU20 | SHU27 | -0.076 |
| SHU45 | SHU58 | -0.075 |
| SHU23 | SHU78 | -0.075 |
| SHU31 | SHU60 | -0.075 |
| SHU3 | SHU12 | -0.075 |
| SHU29 | SHU45 | -0.075 |
| SHU31 | SHU44 | -0.075 |
| SHU30 | SHU34 | -0.075 |
| SHU39 | SHU43 | -0.075 |
| SHU15 | SHU29 | -0.075 |
| SHU1 | SHU76 | -0.075 |
| SHU13 | SHU73 | -0.075 |
| SHU31 | SHU32 | -0.075 |
| SHU32 | SHU72 | -0.075 |
| SHU14 | SHU30 | -0.075 |
| SHU13 | SHU78 | -0.074 |
| SHU13 | SHU40 | -0.074 |
| SHU13 | SHU70 | -0.074 |
| SHU20 | SHU60 | -0.074 |
| SHU8 | SHU31 | -0.074 |
| SHU18 | SHU45 | -0.074 |
| SHU45 | SHU48 | -0.074 |
| SHU47 | SHU72 | -0.074 |
| SHU17 | SHU39 | -0.074 |
| SHU24 | SHU39 | -0.074 |
| SHU47 | SHU54 | -0.073 |
| SHU53 | SHU62 | -0.073 |
| SHU43 | SHU81 | -0.073 |
| SHU35 | SHU80 | -0.073 |
| SHU21 | SHU76 | -0.073 |
| SHU26 | SHU29 | -0.073 |
| SHU7 | SHU41 | -0.072 |
| SHU13 | SHU65 | -0.072 |
| SHU28 | SHU76 | -0.072 |
| SHU12 | SHU62 | -0.072 |
| SHU64 | SHU74 | -0.072 |
| SHU29 | SHU32 | -0.072 |
| SHU6 | SHU38 | -0.072 |
| SHU39 | SHU53 | -0.072 |
| SHU16 | SHU30 | -0.072 |
| SHU17 | SHU25 | -0.072 |
| SHU24 | SHU25 | -0.072 |
| SHU2 | SHU5 | -0.072 |
| SHU19 | SHU37 | -0.072 |
| SHU4 | SHU37 | -0.072 |
| SHU3 | SHU13 | -0.072 |
| SHU17 | SHU81 | -0.072 |
| SHU24 | SHU81 | -0.072 |
| SHU23 | SHU70 | -0.071 |
| SHU13 | SHU55 | -0.071 |
| SHU19 | SHU47 | -0.071 |
| SHU8 | SHU43 | -0.071 |
| SHU47 | SHU66 | -0.071 |
| SHU25 | SHU64 | -0.071 |
| SHU34 | SHU65 | -0.071 |
| SHU8 | SHU53 | -0.071 |
| SHU8 | SHU67 | -0.071 |
| SHU14 | SHU50 | -0.071 |
| SHU47 | SHU52 | -0.071 |
| SHU52 | SHU71 | -0.071 |
| SHU14 | SHU61 | -0.071 |
| SHU35 | SHU50 | -0.071 |
| SHU53 | SHU60 | -0.071 |
| SHU11 | SHU43 | -0.070 |
| SHU76 | SHU81 | -0.070 |
| SHU9 | SHU72 | -0.070 |
| SHU23 | SHU71 | -0.070 |
| SHU13 | SHU75 | -0.070 |
| SHU34 | SHU38 | -0.070 |
| SHU43 | SHU59 | -0.070 |
| SHU1 | SHU74 | -0.070 |
| SHU35 | SHU76 | -0.070 |
| SHU2 | SHU47 | -0.070 |
| SHU27 | SHU74 | -0.069 |
| SHU55 | SHU58 | -0.069 |
| SHU11 | SHU46 | -0.069 |
| SHU26 | SHU27 | -0.069 |
| SHU43 | SHU68 | -0.069 |
| SHU52 | SHU80 | -0.069 |
| SHU15 | SHU45 | -0.069 |
| SHU29 | SHU80 | -0.069 |
| SHU4 | SHU54 | -0.069 |
| SHU3 | SHU23 | -0.069 |
| SHU43 | SHU71 | -0.069 |
| SHU10 | SHU31 | -0.069 |
| SHU5 | SHU42 | -0.069 |
| SHU16 | SHU59 | -0.069 |
| SHU68 | SHU72 | -0.069 |
| SHU22 | SHU23 | -0.069 |
| SHU5 | SHU45 | -0.069 |
| SHU47 | SHU73 | -0.069 |
| SHU33 | SHU54 | -0.068 |
| SHU1 | SHU80 | -0.068 |
| SHU11 | SHU77 | -0.068 |
| SHU2 | SHU41 | -0.068 |
| SHU10 | SHU81 | -0.068 |
| SHU9 | SHU31 | -0.068 |
| SHU15 | SHU53 | -0.068 |
| SHU1 | SHU55 | -0.068 |
| SHU11 | SHU55 | -0.068 |
| SHU9 | SHU20 | -0.068 |
| SHU47 | SHU70 | -0.068 |
| SHU23 | SHU41 | -0.068 |
| SHU18 | SHU55 | -0.068 |
| SHU48 | SHU55 | -0.068 |
| SHU11 | SHU61 | -0.068 |
| SHU5 | SHU67 | -0.068 |
| SHU33 | SHU44 | -0.068 |
| SHU34 | SHU78 | -0.068 |
| SHU60 | SHU63 | -0.067 |
| SHU67 | SHU74 | -0.067 |
| SHU41 | SHU76 | -0.067 |
| SHU26 | SHU53 | -0.067 |
| SHU39 | SHU58 | -0.067 |
| SHU20 | SHU50 | -0.066 |
| SHU27 | SHU32 | -0.066 |
| SHU36 | SHU63 | -0.066 |
| SHU54 | SHU55 | -0.066 |
| SHU57 | SHU74 | -0.066 |
| SHU5 | SHU25 | -0.066 |
| SHU32 | SHU53 | -0.066 |
| SHU25 | SHU55 | -0.066 |
| SHU63 | SHU81 | -0.066 |
| SHU15 | SHU25 | -0.066 |
| SHU2 | SHU30 | -0.066 |
| SHU21 | SHU81 | -0.066 |
| SHU20 | SHU39 | -0.066 |
| SHU17 | SHU71 | -0.065 |
| SHU24 | SHU71 | -0.065 |
| SHU20 | SHU28 | -0.065 |
| SHU7 | SHU20 | -0.065 |
| SHU21 | SHU71 | -0.065 |
| SHU14 | SHU78 | -0.065 |
| SHU12 | SHU59 | -0.065 |
| SHU23 | SHU36 | -0.065 |
| SHU58 | SHU81 | -0.065 |
| SHU13 | SHU58 | -0.065 |
| SHU61 | SHU76 | -0.065 |
| SHU6 | SHU25 | -0.065 |
| SHU18 | SHU39 | -0.065 |
| SHU39 | SHU48 | -0.065 |
| SHU17 | SHU59 | -0.065 |
| SHU24 | SHU59 | -0.065 |
| SHU43 | SHU61 | -0.065 |
| SHU52 | SHU54 | -0.064 |
| SHU20 | SHU71 | -0.064 |
| SHU8 | SHU65 | -0.064 |
| SHU14 | SHU74 | -0.064 |
| SHU23 | SHU60 | -0.064 |
| SHU16 | SHU61 | -0.064 |
| SHU33 | SHU73 | -0.064 |
| SHU17 | SHU62 | -0.064 |
| SHU24 | SHU62 | -0.064 |
| SHU1 | SHU50 | -0.064 |
| SHU45 | SHU55 | -0.064 |
| SHU47 | SHU65 | -0.064 |
| SHU34 | SHU75 | -0.064 |
| SHU9 | SHU47 | -0.064 |
| SHU10 | SHU12 | -0.063 |
| SHU13 | SHU18 | -0.063 |
| SHU13 | SHU48 | -0.063 |
| SHU31 | SHU39 | -0.063 |
| SHU13 | SHU59 | -0.063 |
| SHU33 | SHU70 | -0.063 |
| SHU10 | SHU41 | -0.063 |
| SHU46 | SHU73 | -0.063 |
| SHU25 | SHU43 | -0.063 |
| SHU34 | SHU44 | -0.063 |
| SHU14 | SHU68 | -0.063 |
| SHU18 | SHU81 | -0.063 |
| SHU48 | SHU81 | -0.063 |
| SHU1 | SHU39 | -0.063 |
| SHU9 | SHU10 | -0.063 |
| SHU25 | SHU72 | -0.063 |
| SHU20 | SHU81 | -0.063 |
| SHU4 | SHU57 | -0.063 |
| SHU20 | SHU61 | -0.063 |
| SHU3 | SHU8 | -0.063 |
| SHU60 | SHU74 | -0.063 |
| SHU46 | SHU70 | -0.063 |
| SHU23 | SHU25 | -0.063 |
| SHU39 | SHU72 | -0.063 |
| SHU4 | SHU65 | -0.062 |
| SHU4 | SHU41 | -0.062 |
| SHU6 | SHU19 | -0.062 |
| SHU33 | SHU40 | -0.062 |
| SHU34 | SHU55 | -0.062 |
| SHU53 | SHU61 | -0.062 |
| SHU32 | SHU34 | -0.062 |
| SHU33 | SHU55 | -0.062 |
| SHU14 | SHU75 | -0.062 |
| SHU19 | SHU46 | -0.062 |
| SHU20 | SHU25 | -0.062 |
| SHU14 | SHU22 | -0.062 |
| SHU10 | SHU20 | -0.062 |
| SHU54 | SHU74 | -0.062 |
| SHU51 | SHU63 | -0.061 |
| SHU13 | SHU43 | -0.061 |
| SHU31 | SHU52 | -0.061 |
| SHU22 | SHU35 | -0.061 |
| SHU35 | SHU81 | -0.061 |
| SHU26 | SHU43 | -0.061 |
| SHU31 | SHU81 | -0.061 |
| SHU50 | SHU63 | -0.061 |
| SHU27 | SHU45 | -0.061 |
| SHU11 | SHU41 | -0.061 |
| SHU16 | SHU50 | -0.061 |
| SHU44 | SHU76 | -0.061 |
| SHU5 | SHU63 | -0.061 |
| SHU47 | SHU59 | -0.061 |
| SHU4 | SHU40 | -0.061 |
| SHU45 | SHU64 | -0.061 |
| SHU72 | SHU81 | -0.061 |
| SHU47 | SHU74 | -0.061 |
| SHU11 | SHU25 | -0.061 |
| SHU39 | SHU47 | -0.061 |
| SHU38 | SHU74 | -0.061 |
| SHU5 | SHU57 | -0.060 |
| SHU5 | SHU54 | -0.060 |
| SHU37 | SHU67 | -0.060 |
| SHU41 | SHU55 | -0.060 |
| SHU3 | SHU10 | -0.060 |
| SHU52 | SHU55 | -0.060 |
| SHU17 | SHU61 | -0.060 |
| SHU24 | SHU61 | -0.060 |
| SHU10 | SHU36 | -0.060 |
| SHU15 | SHU43 | -0.060 |
| SHU11 | SHU68 | -0.060 |
| SHU45 | SHU54 | -0.060 |
| SHU1 | SHU72 | -0.060 |
| SHU20 | SHU44 | -0.059 |
| SHU33 | SHU67 | -0.059 |
| SHU12 | SHU58 | -0.059 |
| SHU4 | SHU61 | -0.059 |
| SHU6 | SHU34 | -0.059 |
| SHU30 | SHU31 | -0.059 |
| SHU5 | SHU61 | -0.059 |
| SHU5 | SHU79 | -0.059 |
| SHU8 | SHU62 | -0.059 |
| SHU21 | SHU59 | -0.059 |
| SHU5 | SHU18 | -0.059 |
| SHU5 | SHU48 | -0.059 |
| SHU1 | SHU32 | -0.058 |
| SHU51 | SHU59 | -0.058 |
| SHU5 | SHU80 | -0.058 |
| SHU1 | SHU9 | -0.058 |
| SHU13 | SHU68 | -0.058 |
| SHU10 | SHU51 | -0.058 |
| SHU25 | SHU78 | -0.058 |
| SHU7 | SHU55 | -0.058 |
| SHU16 | SHU78 | -0.058 |
| SHU12 | SHU18 | -0.058 |
| SHU12 | SHU48 | -0.058 |
| SHU29 | SHU81 | -0.058 |
| SHU29 | SHU71 | -0.058 |
| SHU5 | SHU39 | -0.058 |
| SHU10 | SHU50 | -0.058 |
| SHU40 | SHU55 | -0.058 |
| SHU23 | SHU68 | -0.058 |
| SHU18 | SHU47 | -0.058 |
| SHU47 | SHU48 | -0.058 |
| SHU3 | SHU19 | -0.058 |
| SHU31 | SHU50 | -0.057 |
| SHU27 | SHU80 | -0.057 |
| SHU50 | SHU59 | -0.057 |
| SHU33 | SHU60 | -0.057 |
| SHU1 | SHU71 | -0.057 |
| SHU4 | SHU56 | -0.057 |
| SHU52 | SHU81 | -0.057 |
| SHU11 | SHU56 | -0.057 |
| SHU4 | SHU82 | -0.057 |
| SHU16 | SHU68 | -0.057 |
| SHU11 | SHU82 | -0.057 |
| SHU47 | SHU61 | -0.057 |
| SHU5 | SHU55 | -0.057 |
| SHU31 | SHU61 | -0.057 |
| SHU30 | SHU35 | -0.057 |
| SHU37 | SHU38 | -0.056 |
| SHU21 | SHU77 | -0.056 |
| SHU51 | SHU74 | -0.056 |
| SHU7 | SHU11 | -0.056 |
| SHU38 | SHU62 | -0.056 |
| SHU14 | SHU25 | -0.056 |
| SHU20 | SHU80 | -0.056 |
| SHU71 | SHU72 | -0.056 |
| SHU8 | SHU36 | -0.056 |
| SHU16 | SHU75 | -0.056 |
| SHU55 | SHU65 | -0.056 |
| SHU59 | SHU72 | -0.056 |
| SHU42 | SHU76 | -0.056 |
| SHU5 | SHU74 | -0.056 |
| SHU19 | SHU44 | -0.056 |
| SHU12 | SHU49 | -0.056 |
| SHU50 | SHU64 | -0.055 |
| SHU25 | SHU66 | -0.055 |
| SHU45 | SHU57 | -0.055 |
| SHU42 | SHU46 | -0.055 |
| SHU46 | SHU60 | -0.055 |
| SHU6 | SHU51 | -0.055 |
| SHU65 | SHU74 | -0.055 |
| SHU4 | SHU36 | -0.055 |
| SHU53 | SHU74 | -0.055 |
| SHU1 | SHU68 | -0.055 |
| SHU36 | SHU53 | -0.055 |
| SHU5 | SHU50 | -0.055 |
| SHU19 | SHU36 | -0.055 |
| SHU23 | SHU49 | -0.055 |
| SHU46 | SHU54 | -0.054 |
| SHU25 | SHU28 | -0.054 |
| SHU28 | SHU74 | -0.054 |
| SHU6 | SHU50 | -0.054 |
| SHU46 | SHU67 | -0.054 |
| SHU27 | SHU34 | -0.054 |
| SHU6 | SHU42 | -0.054 |
| SHU12 | SHU15 | -0.054 |
| SHU20 | SHU52 | -0.054 |
| SHU15 | SHU80 | -0.054 |
| SHU30 | SHU58 | -0.054 |
| SHU31 | SHU74 | -0.054 |
| SHU45 | SHU47 | -0.054 |
| SHU5 | SHU9 | -0.054 |
| SHU20 | SHU59 | -0.054 |
| SHU3 | SHU47 | -0.054 |
| SHU37 | SHU57 | -0.054 |
| SHU5 | SHU76 | -0.054 |
| SHU5 | SHU23 | -0.054 |
| SHU1 | SHU66 | -0.054 |
| SHU33 | SHU61 | -0.054 |
| SHU20 | SHU63 | -0.054 |
| SHU13 | SHU49 | -0.053 |
| SHU3 | SHU35 | -0.053 |
| SHU10 | SHU39 | -0.053 |
| SHU1 | SHU73 | -0.053 |
| SHU8 | SHU17 | -0.053 |
| SHU8 | SHU24 | -0.053 |
| SHU14 | SHU70 | -0.053 |
| SHU23 | SHU82 | -0.053 |
| SHU40 | SHU74 | -0.053 |
| SHU1 | SHU77 | -0.053 |
| SHU53 | SHU81 | -0.053 |
| SHU35 | SHU77 | -0.053 |
| SHU19 | SHU62 | -0.053 |
| SHU5 | SHU40 | -0.053 |
| SHU34 | SHU62 | -0.053 |
| SHU4 | SHU68 | -0.053 |
| SHU18 | SHU30 | -0.053 |
| SHU30 | SHU48 | -0.053 |
| SHU25 | SHU75 | -0.053 |
| SHU25 | SHU47 | -0.053 |
| SHU54 | SHU63 | -0.052 |
| SHU14 | SHU80 | -0.052 |
| SHU6 | SHU57 | -0.052 |
| SHU6 | SHU41 | -0.052 |
| SHU14 | SHU59 | -0.052 |
| SHU25 | SHU67 | -0.052 |
| SHU56 | SHU64 | -0.052 |
| SHU6 | SHU65 | -0.052 |
| SHU6 | SHU68 | -0.052 |
| SHU52 | SHU62 | -0.052 |
| SHU19 | SHU51 | -0.052 |
| SHU61 | SHU72 | -0.051 |
| SHU70 | SHU74 | -0.051 |
| SHU41 | SHU46 | -0.051 |
| SHU5 | SHU32 | -0.051 |
| SHU8 | SHU16 | -0.051 |
| SHU69 | SHU81 | -0.051 |
| SHU6 | SHU40 | -0.051 |
| SHU35 | SHU65 | -0.051 |
| SHU1 | SHU56 | -0.051 |
| SHU25 | SHU40 | -0.051 |
| SHU76 | SHU79 | -0.051 |
| SHU19 | SHU50 | -0.051 |
| SHU52 | SHU77 | -0.051 |
| SHU6 | SHU9 | -0.051 |
| SHU16 | SHU22 | -0.051 |
| SHU5 | SHU71 | -0.051 |
| SHU37 | SHU60 | -0.051 |
| SHU27 | SHU81 | -0.051 |
| SHU29 | SHU37 | -0.050 |
| SHU36 | SHU52 | -0.050 |
| SHU9 | SHU55 | -0.050 |
| SHU19 | SHU40 | -0.050 |
| SHU55 | SHU59 | -0.050 |
| SHU3 | SHU29 | -0.050 |
| SHU38 | SHU40 | -0.050 |
| SHU2 | SHU61 | -0.050 |
| SHU55 | SHU57 | -0.050 |
| SHU19 | SHU22 | -0.050 |
| SHU19 | SHU71 | -0.050 |
| SHU22 | SHU31 | -0.050 |
| SHU51 | SHU57 | -0.050 |
| SHU3 | SHU45 | -0.050 |
| SHU19 | SHU67 | -0.050 |
| SHU21 | SHU54 | -0.050 |
| SHU26 | SHU74 | -0.050 |
| SHU34 | SHU81 | -0.050 |
| SHU29 | SHU77 | -0.050 |
| SHU5 | SHU41 | -0.050 |
| SHU52 | SHU74 | -0.050 |
| SHU64 | SHU77 | -0.049 |
| SHU5 | SHU68 | -0.049 |
| SHU46 | SHU79 | -0.049 |
| SHU25 | SHU58 | -0.049 |
| SHU64 | SHU80 | -0.049 |
| SHU5 | SHU73 | -0.049 |
| SHU15 | SHU67 | -0.049 |
| SHU51 | SHU55 | -0.049 |
| SHU39 | SHU55 | -0.049 |
| SHU38 | SHU78 | -0.049 |
| SHU6 | SHU39 | -0.049 |
| SHU47 | SHU68 | -0.049 |
| SHU6 | SHU60 | -0.049 |
| SHU5 | SHU66 | -0.049 |
| SHU47 | SHU69 | -0.049 |
| SHU30 | SHU53 | -0.049 |
| SHU5 | SHU69 | -0.049 |
| SHU55 | SHU70 | -0.049 |
| SHU8 | SHU72 | -0.049 |
| SHU23 | SHU77 | -0.049 |
| SHU50 | SHU57 | -0.049 |
| SHU15 | SHU50 | -0.049 |
| SHU31 | SHU75 | -0.049 |
| SHU5 | SHU70 | -0.049 |
| SHU52 | SHU65 | -0.048 |
| SHU10 | SHU25 | -0.048 |
| SHU6 | SHU45 | -0.048 |
| SHU1 | SHU40 | -0.048 |
| SHU15 | SHU44 | -0.048 |
| SHU18 | SHU25 | -0.048 |
| SHU25 | SHU48 | -0.048 |
| SHU29 | SHU50 | -0.048 |
| SHU13 | SHU46 | -0.048 |
| SHU55 | SHU61 | -0.048 |
| SHU21 | SHU55 | -0.048 |
| SHU38 | SHU50 | -0.048 |
| SHU47 | SHU80 | -0.048 |
| SHU8 | SHU63 | -0.048 |
| SHU27 | SHU71 | -0.048 |
| SHU9 | SHU19 | -0.048 |
| SHU13 | SHU41 | -0.048 |
| SHU44 | SHU72 | -0.048 |
| SHU45 | SHU78 | -0.048 |
| SHU26 | SHU55 | -0.047 |
| SHU6 | SHU61 | -0.047 |
| SHU6 | SHU79 | -0.047 |
| SHU34 | SHU53 | -0.047 |
| SHU4 | SHU73 | -0.047 |
| SHU55 | SHU63 | -0.047 |
| SHU6 | SHU47 | -0.047 |
| SHU5 | SHU59 | -0.047 |
| SHU6 | SHU15 | -0.047 |
| SHU21 | SHU75 | -0.047 |
| SHU4 | SHU55 | -0.047 |
| SHU8 | SHU59 | -0.047 |
| SHU22 | SHU38 | -0.047 |
| SHU32 | SHU47 | -0.047 |
| SHU30 | SHU38 | -0.047 |
| SHU17 | SHU55 | -0.047 |
| SHU24 | SHU55 | -0.047 |
| SHU45 | SHU66 | -0.047 |
| SHU63 | SHU74 | -0.047 |
| SHU36 | SHU45 | -0.047 |
| SHU25 | SHU31 | -0.047 |
| SHU4 | SHU70 | -0.047 |
| SHU8 | SHU68 | -0.047 |
| SHU3 | SHU42 | -0.047 |
| SHU56 | SHU57 | -0.046 |
| SHU11 | SHU49 | -0.046 |
| SHU20 | SHU30 | -0.046 |
| SHU37 | SHU65 | -0.046 |
| SHU57 | SHU82 | -0.046 |
| SHU33 | SHU68 | -0.046 |
| SHU35 | SHU78 | -0.046 |
| SHU19 | SHU39 | -0.046 |
| SHU59 | SHU63 | -0.046 |
| SHU16 | SHU70 | -0.046 |
| SHU1 | SHU41 | -0.046 |
| SHU15 | SHU46 | -0.046 |
| SHU25 | SHU38 | -0.046 |
| SHU46 | SHU65 | -0.046 |
| SHU38 | SHU71 | -0.046 |
| SHU40 | SHU51 | -0.046 |
| SHU42 | SHU44 | -0.046 |
| SHU59 | SHU67 | -0.046 |
| SHU13 | SHU62 | -0.046 |
| SHU28 | SHU37 | -0.046 |
| SHU46 | SHU71 | -0.046 |
| SHU1 | SHU81 | -0.045 |
| SHU19 | SHU77 | -0.045 |
| SHU58 | SHU59 | -0.045 |
| SHU16 | SHU74 | -0.045 |
| SHU46 | SHU66 | -0.045 |
| SHU11 | SHU71 | -0.045 |
| SHU43 | SHU80 | -0.045 |
| SHU15 | SHU71 | -0.045 |
| SHU12 | SHU61 | -0.045 |
| SHU32 | SHU37 | -0.045 |
| SHU59 | SHU68 | -0.045 |
| SHU44 | SHU55 | -0.045 |
| SHU11 | SHU15 | -0.045 |
| SHU45 | SHU73 | -0.045 |
| SHU1 | SHU70 | -0.045 |
| SHU9 | SHU69 | -0.045 |
| SHU41 | SHU69 | -0.045 |
| SHU36 | SHU55 | -0.044 |
| SHU40 | SHU50 | -0.044 |
| SHU25 | SHU30 | -0.044 |
| SHU3 | SHU55 | -0.044 |
| SHU5 | SHU77 | -0.044 |
| SHU19 | SHU45 | -0.044 |
| SHU40 | SHU67 | -0.044 |
| SHU30 | SHU76 | -0.044 |
| SHU10 | SHU16 | -0.044 |
| SHU36 | SHU42 | -0.044 |
| SHU13 | SHU61 | -0.044 |
| SHU45 | SHU70 | -0.044 |
| SHU18 | SHU59 | -0.044 |
| SHU48 | SHU59 | -0.044 |
| SHU3 | SHU38 | -0.044 |
| SHU53 | SHU80 | -0.044 |
| SHU22 | SHU53 | -0.044 |
| SHU15 | SHU56 | -0.044 |
| SHU46 | SHU62 | -0.044 |
| SHU57 | SHU80 | -0.044 |
| SHU45 | SHU46 | -0.044 |
| SHU7 | SHU67 | -0.044 |
| SHU37 | SHU73 | -0.043 |
| SHU13 | SHU36 | -0.043 |
| SHU7 | SHU75 | -0.043 |
| SHU33 | SHU50 | -0.043 |
| SHU45 | SHU75 | -0.043 |
| SHU55 | SHU80 | -0.043 |
| SHU36 | SHU67 | -0.043 |
| SHU37 | SHU66 | -0.043 |
| SHU30 | SHU69 | -0.043 |
| SHU57 | SHU77 | -0.043 |
| SHU31 | SHU80 | -0.043 |
| SHU6 | SHU31 | -0.043 |
| SHU38 | SHU70 | -0.043 |
| SHU37 | SHU70 | -0.043 |
| SHU37 | SHU54 | -0.043 |
| SHU30 | SHU74 | -0.043 |
| SHU19 | SHU30 | -0.042 |
| SHU15 | SHU22 | -0.042 |
| SHU4 | SHU49 | -0.042 |
| SHU64 | SHU68 | -0.042 |
| SHU32 | SHU38 | -0.042 |
| SHU31 | SHU59 | -0.042 |
| SHU38 | SHU41 | -0.042 |
| SHU13 | SHU33 | -0.042 |
| SHU13 | SHU72 | -0.042 |
| SHU20 | SHU26 | -0.042 |
| SHU51 | SHU66 | -0.042 |
| SHU34 | SHU61 | -0.042 |
| SHU6 | SHU73 | -0.042 |
| SHU17 | SHU54 | -0.042 |
| SHU24 | SHU54 | -0.042 |
| SHU42 | SHU66 | -0.042 |
| SHU54 | SHU82 | -0.042 |
| SHU2 | SHU78 | -0.042 |
| SHU6 | SHU80 | -0.042 |
| SHU39 | SHU64 | -0.042 |
| SHU30 | SHU43 | -0.042 |
| SHU58 | SHU61 | -0.042 |
| SHU36 | SHU38 | -0.042 |
| SHU25 | SHU65 | -0.041 |
| SHU42 | SHU55 | -0.041 |
| SHU36 | SHU59 | -0.041 |
| SHU3 | SHU34 | -0.041 |
| SHU3 | SHU57 | -0.041 |
| SHU20 | SHU77 | -0.041 |
| SHU3 | SHU25 | -0.041 |
| SHU37 | SHU40 | -0.041 |
| SHU1 | SHU12 | -0.041 |
| SHU34 | SHU50 | -0.041 |
| SHU50 | SHU66 | -0.041 |
| SHU6 | SHU66 | -0.041 |
| SHU2 | SHU75 | -0.041 |
| SHU45 | SHU68 | -0.041 |
| SHU18 | SHU61 | -0.040 |
| SHU48 | SHU61 | -0.040 |
| SHU28 | SHU55 | -0.040 |
| SHU45 | SHU65 | -0.040 |
| SHU11 | SHU22 | -0.040 |
| SHU20 | SHU22 | -0.040 |
| SHU57 | SHU67 | -0.040 |
| SHU5 | SHU62 | -0.040 |
| SHU51 | SHU73 | -0.040 |
| SHU19 | SHU75 | -0.040 |
| SHU15 | SHU77 | -0.040 |
| SHU74 | SHU81 | -0.040 |
| SHU25 | SHU62 | -0.040 |
| SHU5 | SHU81 | -0.040 |
| SHU46 | SHU55 | -0.040 |
| SHU42 | SHU73 | -0.040 |
| SHU38 | SHU60 | -0.040 |
| SHU9 | SHU64 | -0.040 |
| SHU5 | SHU56 | -0.040 |
| SHU68 | SHU74 | -0.040 |
| SHU3 | SHU51 | -0.040 |
| SHU3 | SHU64 | -0.040 |
| SHU6 | SHU64 | -0.040 |
| SHU22 | SHU54 | -0.040 |
| SHU50 | SHU75 | -0.040 |
| SHU17 | SHU35 | -0.040 |
| SHU24 | SHU35 | -0.040 |
| SHU53 | SHU78 | -0.039 |
| SHU19 | SHU56 | -0.039 |
| SHU32 | SHU46 | -0.039 |
| SHU8 | SHU49 | -0.039 |
| SHU19 | SHU82 | -0.039 |
| SHU51 | SHU70 | -0.039 |
| SHU67 | SHU75 | -0.039 |
| SHU6 | SHU32 | -0.039 |
| SHU59 | SHU73 | -0.039 |
| SHU12 | SHU68 | -0.039 |
| SHU25 | SHU59 | -0.039 |
| SHU50 | SHU73 | -0.039 |
| SHU42 | SHU68 | -0.039 |
| SHU3 | SHU69 | -0.039 |
| SHU3 | SHU20 | -0.039 |
| SHU6 | SHU71 | -0.039 |
| SHU3 | SHU50 | -0.039 |
| SHU44 | SHU62 | -0.039 |
| SHU64 | SHU71 | -0.039 |
| SHU56 | SHU66 | -0.039 |
| SHU2 | SHU36 | -0.039 |
| SHU78 | SHU80 | -0.039 |
| SHU10 | SHU35 | -0.038 |
| SHU45 | SHU60 | -0.038 |
| SHU55 | SHU81 | -0.038 |
| SHU21 | SHU70 | -0.038 |
| SHU59 | SHU70 | -0.038 |
| SHU3 | SHU27 | -0.038 |
| SHU5 | SHU38 | -0.038 |
| SHU62 | SHU68 | -0.038 |
| SHU59 | SHU60 | -0.038 |
| SHU50 | SHU70 | -0.038 |
| SHU19 | SHU80 | -0.038 |
| SHU25 | SHU44 | -0.038 |
| SHU40 | SHU59 | -0.038 |
| SHU38 | SHU82 | -0.038 |
| SHU23 | SHU35 | -0.038 |
| SHU45 | SHU67 | -0.038 |
| SHU41 | SHU67 | -0.038 |
| SHU23 | SHU56 | -0.038 |
| SHU45 | SHU76 | -0.038 |
| SHU67 | SHU68 | -0.038 |
| SHU25 | SHU32 | -0.038 |
| SHU37 | SHU50 | -0.038 |
| SHU47 | SHU71 | -0.038 |
| SHU41 | SHU74 | -0.038 |
| SHU74 | SHU78 | -0.038 |
| SHU28 | SHU56 | -0.037 |
| SHU16 | SHU25 | -0.037 |
| SHU11 | SHU62 | -0.037 |
| SHU1 | SHU62 | -0.037 |
| SHU6 | SHU55 | -0.037 |
| SHU61 | SHU74 | -0.037 |
| SHU67 | SHU77 | -0.037 |
| SHU28 | SHU82 | -0.037 |
| SHU37 | SHU55 | -0.037 |
| SHU3 | SHU28 | -0.037 |
| SHU44 | SHU79 | -0.037 |
| SHU3 | SHU79 | -0.037 |
| SHU27 | SHU76 | -0.037 |
| SHU56 | SHU73 | -0.037 |
| SHU9 | SHU67 | -0.037 |
| SHU22 | SHU57 | -0.037 |
| SHU73 | SHU82 | -0.037 |
| SHU4 | SHU35 | -0.037 |
| SHU66 | SHU80 | -0.036 |
| SHU28 | SHU77 | -0.036 |
| SHU38 | SHU68 | -0.036 |
| SHU47 | SHU81 | -0.036 |
| SHU56 | SHU70 | -0.036 |
| SHU65 | SHU69 | -0.036 |
| SHU32 | SHU62 | -0.036 |
| SHU51 | SHU54 | -0.036 |
| SHU9 | SHU76 | -0.036 |
| SHU27 | SHU59 | -0.036 |
| SHU44 | SHU57 | -0.036 |
| SHU35 | SHU72 | -0.036 |
| SHU39 | SHU67 | -0.036 |
| SHU70 | SHU82 | -0.036 |
| SHU51 | SHU62 | -0.036 |
| SHU55 | SHU79 | -0.036 |
| SHU51 | SHU65 | -0.036 |
| SHU63 | SHU80 | -0.036 |
| SHU76 | SHU78 | -0.036 |
| SHU9 | SHU46 | -0.036 |
| SHU42 | SHU63 | -0.036 |
| SHU6 | SHU20 | -0.036 |
| SHU41 | SHU64 | -0.036 |
| SHU1 | SHU30 | -0.036 |
| SHU47 | SHU64 | -0.036 |
| SHU16 | SHU80 | -0.035 |
| SHU36 | SHU74 | -0.035 |
| SHU17 | SHU80 | -0.035 |
| SHU24 | SHU80 | -0.035 |
| SHU4 | SHU21 | -0.035 |
| SHU64 | SHU82 | -0.035 |
| SHU40 | SHU56 | -0.035 |
| SHU26 | SHU46 | -0.035 |
| SHU54 | SHU56 | -0.035 |
| SHU54 | SHU72 | -0.035 |
| SHU41 | SHU44 | -0.035 |
| SHU1 | SHU11 | -0.035 |
| SHU50 | SHU62 | -0.035 |
| SHU40 | SHU82 | -0.035 |
| SHU17 | SHU29 | -0.035 |
| SHU24 | SHU29 | -0.035 |
| SHU36 | SHU79 | -0.035 |
| SHU37 | SHU61 | -0.035 |
| SHU21 | SHU56 | -0.035 |
| SHU38 | SHU49 | -0.035 |
| SHU5 | SHU72 | -0.035 |
| SHU73 | SHU80 | -0.035 |
| SHU58 | SHU78 | -0.035 |
| SHU14 | SHU77 | -0.034 |
| SHU39 | SHU57 | -0.034 |
| SHU17 | SHU30 | -0.034 |
| SHU24 | SHU30 | -0.034 |
| SHU39 | SHU78 | -0.034 |
| SHU63 | SHU79 | -0.034 |
| SHU37 | SHU81 | -0.034 |
| SHU61 | SHU62 | -0.034 |
| SHU44 | SHU78 | -0.034 |
| SHU54 | SHU78 | -0.034 |
| SHU75 | SHU80 | -0.034 |
| SHU63 | SHU75 | -0.034 |
| SHU70 | SHU80 | -0.034 |
| SHU22 | SHU47 | -0.034 |
| SHU28 | SHU62 | -0.034 |
| SHU33 | SHU81 | -0.034 |
| SHU3 | SHU76 | -0.034 |
| SHU45 | SHU61 | -0.034 |
| SHU60 | SHU64 | -0.034 |
| SHU36 | SHU76 | -0.034 |
| SHU27 | SHU77 | -0.034 |
| SHU55 | SHU71 | -0.034 |
| SHU15 | SHU39 | -0.034 |
| SHU47 | SHU77 | -0.034 |
| SHU18 | SHU78 | -0.034 |
| SHU48 | SHU78 | -0.034 |
| SHU3 | SHU41 | -0.034 |
| SHU44 | SHU60 | -0.033 |
| SHU22 | SHU65 | -0.033 |
| SHU13 | SHU42 | -0.033 |
| SHU52 | SHU56 | -0.033 |
| SHU36 | SHU57 | -0.033 |
| SHU56 | SHU67 | -0.033 |
| SHU38 | SHU77 | -0.033 |
| SHU62 | SHU65 | -0.033 |
| SHU28 | SHU61 | -0.033 |
| SHU40 | SHU80 | -0.033 |
| SHU28 | SHU79 | -0.033 |
| SHU54 | SHU80 | -0.033 |
| SHU8 | SHU41 | -0.033 |
| SHU67 | SHU82 | -0.033 |
| SHU9 | SHU78 | -0.033 |
| SHU35 | SHU56 | -0.033 |
| SHU14 | SHU69 | -0.033 |
| SHU11 | SHU54 | -0.033 |
| SHU28 | SHU54 | -0.033 |
| SHU1 | SHU26 | -0.033 |
| SHU57 | SHU62 | -0.033 |
| SHU12 | SHU42 | -0.033 |
| SHU12 | SHU76 | -0.033 |
| SHU26 | SHU62 | -0.033 |
| SHU32 | SHU54 | -0.033 |
| SHU14 | SHU27 | -0.033 |
| SHU18 | SHU75 | -0.033 |
| SHU48 | SHU75 | -0.033 |
| SHU10 | SHU53 | -0.033 |
| SHU26 | SHU31 | -0.033 |
| SHU28 | SHU67 | -0.033 |
| SHU37 | SHU42 | -0.033 |
| SHU37 | SHU44 | -0.032 |
| SHU27 | SHU37 | -0.032 |
| SHU46 | SHU50 | -0.032 |
| SHU6 | SHU74 | -0.032 |
| SHU30 | SHU33 | -0.032 |
| SHU22 | SHU55 | -0.032 |
| SHU56 | SHU60 | -0.032 |
| SHU30 | SHU59 | -0.032 |
| SHU9 | SHU57 | -0.032 |
| SHU61 | SHU66 | -0.032 |
| SHU29 | SHU72 | -0.032 |
| SHU66 | SHU79 | -0.032 |
| SHU6 | SHU54 | -0.032 |
| SHU69 | SHU71 | -0.032 |
| SHU42 | SHU75 | -0.032 |
| SHU67 | SHU80 | -0.032 |
| SHU60 | SHU82 | -0.032 |
| SHU54 | SHU62 | -0.032 |
| SHU16 | SHU29 | -0.032 |
| SHU36 | SHU58 | -0.032 |
| SHU3 | SHU32 | -0.032 |
| SHU35 | SHU38 | -0.032 |
| SHU11 | SHU20 | -0.032 |
| SHU42 | SHU70 | -0.032 |
| SHU40 | SHU77 | -0.032 |
| SHU32 | SHU78 | -0.031 |
| SHU43 | SHU78 | -0.031 |
| SHU59 | SHU81 | -0.031 |
| SHU20 | SHU56 | -0.031 |
| SHU60 | SHU77 | -0.031 |
| SHU42 | SHU74 | -0.031 |
| SHU6 | SHU59 | -0.031 |
| SHU4 | SHU53 | -0.031 |
| SHU39 | SHU46 | -0.031 |
| SHU12 | SHU46 | -0.031 |
| SHU17 | SHU34 | -0.031 |
| SHU24 | SHU34 | -0.031 |
| SHU44 | SHU65 | -0.031 |
| SHU54 | SHU71 | -0.031 |
| SHU1 | SHU82 | -0.031 |
| SHU44 | SHU82 | -0.031 |
| SHU29 | SHU52 | -0.031 |
| SHU73 | SHU77 | -0.031 |
| SHU18 | SHU36 | -0.031 |
| SHU36 | SHU48 | -0.031 |
| SHU9 | SHU33 | -0.031 |
| SHU40 | SHU64 | -0.031 |
| SHU72 | SHU80 | -0.031 |
| SHU39 | SHU69 | -0.031 |
| SHU34 | SHU42 | -0.031 |
| SHU28 | SHU68 | -0.031 |
| SHU30 | SHU54 | -0.031 |
| SHU40 | SHU44 | -0.031 |
| SHU35 | SHU44 | -0.031 |
| SHU27 | SHU50 | -0.031 |
| SHU7 | SHU9 | -0.031 |
| SHU25 | SHU26 | -0.031 |
| SHU13 | SHU79 | -0.031 |
| SHU25 | SHU49 | -0.030 |
| SHU47 | SHU55 | -0.030 |
| SHU3 | SHU33 | -0.030 |
| SHU28 | SHU78 | -0.030 |
| SHU68 | SHU79 | -0.030 |
| SHU60 | SHU80 | -0.030 |
| SHU41 | SHU51 | -0.030 |
| SHU15 | SHU30 | -0.030 |
| SHU17 | SHU33 | -0.030 |
| SHU24 | SHU33 | -0.030 |
| SHU15 | SHU82 | -0.030 |
| SHU70 | SHU77 | -0.030 |
| SHU32 | SHU51 | -0.030 |
| SHU12 | SHU79 | -0.030 |
| SHU14 | SHU54 | -0.030 |
| SHU73 | SHU79 | -0.030 |
| SHU22 | SHU68 | -0.030 |
| SHU32 | SHU36 | -0.030 |
| SHU29 | SHU56 | -0.030 |
| SHU31 | SHU69 | -0.030 |
| SHU65 | SHU82 | -0.030 |
| SHU51 | SHU77 | -0.030 |
| SHU32 | SHU42 | -0.030 |
| SHU46 | SHU81 | -0.030 |
| SHU36 | SHU64 | -0.030 |
| SHU6 | SHU81 | -0.030 |
| SHU3 | SHU73 | -0.030 |
| SHU30 | SHU77 | -0.030 |
| SHU3 | SHU43 | -0.030 |
| SHU65 | SHU78 | -0.030 |
| SHU27 | SHU31 | -0.030 |
| SHU57 | SHU78 | -0.029 |
| SHU52 | SHU53 | -0.029 |
| SHU32 | SHU40 | -0.029 |
| SHU51 | SHU82 | -0.029 |
| SHU30 | SHU72 | -0.029 |
| SHU61 | SHU78 | -0.029 |
| SHU6 | SHU77 | -0.029 |
| SHU51 | SHU78 | -0.029 |
| SHU9 | SHU59 | -0.029 |
| SHU39 | SHU76 | -0.029 |
| SHU33 | SHU65 | -0.029 |
| SHU32 | SHU67 | -0.029 |
| SHU19 | SHU49 | -0.029 |
| SHU3 | SHU53 | -0.029 |
| SHU51 | SHU71 | -0.029 |
| SHU50 | SHU82 | -0.029 |
| SHU22 | SHU64 | -0.029 |
| SHU55 | SHU60 | -0.029 |
| SHU34 | SHU59 | -0.029 |
| SHU26 | SHU44 | -0.029 |
| SHU45 | SHU62 | -0.029 |
| SHU62 | SHU64 | -0.029 |
| SHU22 | SHU32 | -0.029 |
| SHU51 | SHU61 | -0.029 |
| SHU26 | SHU78 | -0.029 |
| SHU64 | SHU81 | -0.029 |
| SHU15 | SHU73 | -0.029 |
| SHU37 | SHU68 | -0.029 |
| SHU33 | SHU71 | -0.029 |
| SHU50 | SHU78 | -0.029 |
| SHU28 | SHU36 | -0.029 |
| SHU68 | SHU75 | -0.028 |
| SHU3 | SHU31 | -0.028 |
| SHU25 | SHU81 | -0.028 |
| SHU30 | SHU56 | -0.028 |
| SHU40 | SHU61 | -0.028 |
| SHU60 | SHU61 | -0.028 |
| SHU55 | SHU82 | -0.028 |
| SHU26 | SHU42 | -0.028 |
| SHU50 | SHU71 | -0.028 |
| SHU22 | SHU51 | -0.028 |
| SHU22 | SHU73 | -0.028 |
| SHU34 | SHU79 | -0.028 |
| SHU50 | SHU61 | -0.028 |
| SHU5 | SHU30 | -0.028 |
| SHU39 | SHU75 | -0.028 |
| SHU34 | SHU72 | -0.028 |
| SHU39 | SHU59 | -0.028 |
| SHU35 | SHU57 | -0.028 |
| SHU22 | SHU66 | -0.028 |
| SHU6 | SHU62 | -0.028 |
| SHU29 | SHU44 | -0.028 |
| SHU31 | SHU77 | -0.028 |
| SHU52 | SHU76 | -0.028 |
| SHU12 | SHU41 | -0.028 |
| SHU22 | SHU50 | -0.028 |
| SHU37 | SHU79 | -0.028 |
| SHU74 | SHU79 | -0.028 |
| SHU32 | SHU71 | -0.028 |
| SHU30 | SHU52 | -0.028 |
| SHU22 | SHU70 | -0.028 |
| SHU15 | SHU62 | -0.028 |
| SHU69 | SHU78 | -0.028 |
| SHU44 | SHU54 | -0.028 |
| SHU22 | SHU61 | -0.027 |
| SHU65 | SHU77 | -0.027 |
| SHU43 | SHU77 | -0.027 |
| SHU46 | SHU68 | -0.027 |
| SHU15 | SHU41 | -0.027 |
| SHU15 | SHU70 | -0.027 |
| SHU28 | SHU71 | -0.027 |
| SHU77 | SHU78 | -0.027 |
| SHU21 | SHU53 | -0.027 |
| SHU30 | SHU65 | -0.027 |
| SHU23 | SHU47 | -0.027 |
| SHU41 | SHU66 | -0.027 |
| SHU22 | SHU26 | -0.027 |
| SHU64 | SHU70 | -0.027 |
| SHU30 | SHU46 | -0.027 |
| SHU38 | SHU56 | -0.027 |
| SHU6 | SHU36 | -0.027 |
| SHU9 | SHU15 | -0.027 |
| SHU73 | SHU78 | -0.027 |
| SHU46 | SHU59 | -0.027 |
| SHU53 | SHU77 | -0.027 |
| SHU49 | SHU57 | -0.027 |
| SHU65 | SHU73 | -0.027 |
| SHU19 | SHU81 | -0.027 |
| SHU33 | SHU72 | -0.027 |
| SHU15 | SHU81 | -0.027 |
| SHU40 | SHU68 | -0.027 |
| SHU37 | SHU53 | -0.027 |
| SHU33 | SHU39 | -0.027 |
| SHU30 | SHU68 | -0.027 |
| SHU25 | SHU51 | -0.027 |
| SHU36 | SHU62 | -0.027 |
| SHU69 | SHU75 | -0.027 |
| SHU32 | SHU75 | -0.027 |
| SHU33 | SHU78 | -0.027 |
| SHU29 | SHU59 | -0.027 |
| SHU54 | SHU77 | -0.027 |
| SHU3 | SHU11 | -0.027 |
| SHU22 | SHU40 | -0.026 |
| SHU36 | SHU41 | -0.026 |
| SHU4 | SHU64 | -0.026 |
| SHU60 | SHU75 | -0.026 |
| SHU30 | SHU41 | -0.026 |
| SHU9 | SHU75 | -0.026 |
| SHU68 | SHU82 | -0.026 |
| SHU44 | SHU59 | -0.026 |
| SHU12 | SHU71 | -0.026 |
| SHU6 | SHU35 | -0.026 |
| SHU38 | SHU80 | -0.026 |
| SHU54 | SHU75 | -0.026 |
| SHU56 | SHU65 | -0.026 |
| SHU62 | SHU63 | -0.026 |
| SHU25 | SHU36 | -0.026 |
| SHU3 | SHU40 | -0.026 |
| SHU3 | SHU62 | -0.026 |
| SHU20 | SHU49 | -0.026 |
| SHU34 | SHU41 | -0.026 |
| SHU25 | SHU61 | -0.026 |
| SHU71 | SHU75 | -0.026 |
| SHU39 | SHU73 | -0.026 |
| SHU32 | SHU68 | -0.026 |
| SHU32 | SHU41 | -0.026 |
| SHU62 | SHU81 | -0.026 |
| SHU39 | SHU60 | -0.025 |
| SHU22 | SHU43 | -0.025 |
| SHU26 | SHU71 | -0.025 |
| SHU11 | SHU70 | -0.025 |
| SHU3 | SHU61 | -0.025 |
| SHU36 | SHU77 | -0.025 |
| SHU36 | SHU66 | -0.025 |
| SHU49 | SHU54 | -0.025 |
| SHU10 | SHU60 | -0.025 |
| SHU8 | SHU61 | -0.025 |
| SHU5 | SHU26 | -0.025 |
| SHU49 | SHU55 | -0.025 |
| SHU44 | SHU73 | -0.025 |
| SHU32 | SHU70 | -0.025 |
| SHU44 | SHU49 | -0.025 |
| SHU66 | SHU82 | -0.025 |
| SHU45 | SHU59 | -0.025 |
| SHU36 | SHU73 | -0.025 |
| SHU61 | SHU75 | -0.025 |
| SHU75 | SHU79 | -0.025 |
| SHU39 | SHU70 | -0.025 |
| SHU3 | SHU22 | -0.025 |
| SHU26 | SHU40 | -0.025 |
| SHU65 | SHU80 | -0.025 |
| SHU56 | SHU78 | -0.025 |
| SHU37 | SHU45 | -0.025 |
| SHU53 | SHU59 | -0.025 |
| SHU68 | SHU71 | -0.025 |
| SHU65 | SHU68 | -0.025 |
| SHU5 | SHU78 | -0.024 |
| SHU44 | SHU70 | -0.024 |
| SHU3 | SHU80 | -0.024 |
| SHU33 | SHU75 | -0.024 |
| SHU23 | SHU26 | -0.024 |
| SHU21 | SHU50 | -0.024 |
| SHU34 | SHU71 | -0.024 |
| SHU26 | SHU77 | -0.024 |
| SHU51 | SHU56 | -0.024 |
| SHU65 | SHU75 | -0.024 |
| SHU44 | SHU61 | -0.024 |
| SHU32 | SHU79 | -0.024 |
| SHU36 | SHU70 | -0.024 |
| SHU46 | SHU61 | -0.024 |
| SHU64 | SHU78 | -0.024 |
| SHU49 | SHU51 | -0.024 |
| SHU57 | SHU75 | -0.024 |
| SHU1 | SHU60 | -0.024 |
| SHU50 | SHU56 | -0.024 |
| SHU32 | SHU60 | -0.024 |
| SHU41 | SHU61 | -0.024 |
| SHU39 | SHU65 | -0.024 |
| SHU61 | SHU70 | -0.024 |
| SHU39 | SHU66 | -0.024 |
| SHU36 | SHU54 | -0.024 |
| SHU26 | SHU75 | -0.024 |
| SHU70 | SHU79 | -0.024 |
| SHU25 | SHU76 | -0.024 |
| SHU36 | SHU71 | -0.024 |
| SHU13 | SHU69 | -0.024 |
| SHU75 | SHU77 | -0.024 |
| SHU28 | SHU60 | -0.024 |
| SHU71 | SHU73 | -0.024 |
| SHU40 | SHU57 | -0.024 |
| SHU3 | SHU30 | -0.024 |
| SHU36 | SHU61 | -0.023 |
| SHU26 | SHU50 | -0.023 |
| SHU42 | SHU49 | -0.023 |
| SHU49 | SHU50 | -0.023 |
| SHU41 | SHU65 | -0.023 |
| SHU51 | SHU80 | -0.023 |
| SHU57 | SHU81 | -0.023 |
| SHU9 | SHU73 | -0.023 |
| SHU15 | SHU34 | -0.023 |
| SHU62 | SHU80 | -0.023 |
| SHU51 | SHU68 | -0.023 |
| SHU41 | SHU75 | -0.023 |
| SHU37 | SHU41 | -0.023 |
| SHU49 | SHU74 | -0.023 |
| SHU6 | SHU29 | -0.023 |
| SHU36 | SHU56 | -0.023 |
| SHU34 | SHU49 | -0.023 |
| SHU1 | SHU78 | -0.023 |
| SHU11 | SHU78 | -0.023 |
| SHU30 | SHU37 | -0.023 |
| SHU70 | SHU71 | -0.023 |
| SHU51 | SHU64 | -0.023 |
| SHU49 | SHU67 | -0.023 |
| SHU36 | SHU39 | -0.023 |
| SHU33 | SHU42 | -0.023 |
| SHU4 | SHU62 | -0.023 |
| SHU15 | SHU40 | -0.023 |
| SHU40 | SHU71 | -0.023 |
| SHU26 | SHU61 | -0.023 |
| SHU8 | SHU10 | -0.022 |
| SHU40 | SHU79 | -0.022 |
| SHU71 | SHU76 | -0.022 |
| SHU30 | SHU40 | -0.022 |
| SHU55 | SHU56 | -0.022 |
| SHU36 | SHU78 | -0.022 |
| SHU44 | SHU56 | -0.022 |
| SHU26 | SHU73 | -0.022 |
| SHU9 | SHU36 | -0.022 |
| SHU3 | SHU78 | -0.022 |
| SHU30 | SHU64 | -0.022 |
| SHU42 | SHU51 | -0.022 |
| SHU9 | SHU70 | -0.022 |
| SHU49 | SHU64 | -0.022 |
| SHU28 | SHU70 | -0.022 |
| SHU37 | SHU52 | -0.022 |
| SHU44 | SHU81 | -0.022 |
| SHU41 | SHU59 | -0.022 |
| SHU51 | SHU60 | -0.022 |
| SHU21 | SHU60 | -0.022 |
| SHU78 | SHU81 | -0.022 |
| SHU28 | SHU49 | -0.022 |
| SHU17 | SHU78 | -0.022 |
| SHU24 | SHU78 | -0.022 |
| SHU3 | SHU81 | -0.022 |
| SHU56 | SHU75 | -0.022 |
| SHU54 | SHU67 | -0.022 |
| SHU9 | SHU66 | -0.022 |
| SHU66 | SHU70 | -0.022 |
| SHU32 | SHU56 | -0.022 |
| SHU54 | SHU66 | -0.022 |
| SHU42 | SHU50 | -0.022 |
| SHU36 | SHU80 | -0.022 |
| SHU47 | SHU56 | -0.022 |
| SHU32 | SHU82 | -0.022 |
| SHU47 | SHU82 | -0.022 |
| SHU30 | SHU55 | -0.022 |
| SHU49 | SHU71 | -0.022 |
| SHU1 | SHU49 | -0.022 |
| SHU51 | SHU67 | -0.022 |
| SHU15 | SHU51 | -0.022 |
| SHU26 | SHU56 | -0.022 |
| SHU40 | SHU66 | -0.022 |
| SHU63 | SHU70 | -0.022 |
| SHU22 | SHU58 | -0.022 |
| SHU60 | SHU66 | -0.022 |
| SHU22 | SHU67 | -0.022 |
| SHU11 | SHU33 | -0.022 |
| SHU66 | SHU75 | -0.022 |
| SHU26 | SHU82 | -0.022 |
| SHU6 | SHU53 | -0.021 |
| SHU36 | SHU44 | -0.021 |
| SHU42 | SHU81 | -0.021 |
| SHU15 | SHU23 | -0.021 |
| SHU50 | SHU67 | -0.021 |
| SHU50 | SHU81 | -0.021 |
| SHU26 | SHU60 | -0.021 |
| SHU25 | SHU46 | -0.021 |
| SHU40 | SHU65 | -0.021 |
| SHU17 | SHU75 | -0.021 |
| SHU24 | SHU75 | -0.021 |
| SHU34 | SHU63 | -0.021 |
| SHU18 | SHU22 | -0.021 |
| SHU22 | SHU48 | -0.021 |
| SHU59 | SHU80 | -0.021 |
| SHU38 | SHU47 | -0.021 |
| SHU54 | SHU81 | -0.021 |
| SHU22 | SHU60 | -0.021 |
| SHU46 | SHU78 | -0.021 |
| SHU36 | SHU81 | -0.021 |
| SHU18 | SHU70 | -0.021 |
| SHU48 | SHU70 | -0.021 |
| SHU29 | SHU33 | -0.021 |
| SHU4 | SHU60 | -0.021 |
| SHU26 | SHU80 | -0.021 |
| SHU62 | SHU77 | -0.021 |
| SHU22 | SHU81 | -0.020 |
| SHU59 | SHU78 | -0.020 |
| SHU26 | SHU30 | -0.020 |
| SHU6 | SHU56 | -0.020 |
| SHU3 | SHU36 | -0.020 |
| SHU39 | SHU54 | -0.020 |
| SHU9 | SHU60 | -0.020 |
| SHU3 | SHU68 | -0.020 |
| SHU47 | SHU62 | -0.020 |
| SHU17 | SHU36 | -0.020 |
| SHU24 | SHU36 | -0.020 |
| SHU10 | SHU75 | -0.020 |
| SHU33 | SHU79 | -0.020 |
| SHU49 | SHU65 | -0.020 |
| SHU26 | SHU37 | -0.020 |
| SHU4 | SHU43 | -0.020 |
| SHU61 | SHU82 | -0.020 |
| SHU26 | SHU68 | -0.020 |
| SHU15 | SHU75 | -0.020 |
| SHU44 | SHU68 | -0.020 |
| SHU26 | SHU41 | -0.020 |
| SHU30 | SHU66 | -0.020 |
| SHU6 | SHU76 | -0.020 |
| SHU25 | SHU42 | -0.020 |
| SHU49 | SHU68 | -0.020 |
| SHU41 | SHU49 | -0.020 |
| SHU49 | SHU73 | -0.020 |
| SHU3 | SHU7 | -0.020 |
| SHU4 | SHU26 | -0.020 |
| SHU61 | SHU68 | -0.020 |
| SHU60 | SHU71 | -0.019 |
| SHU33 | SHU53 | -0.019 |
| SHU3 | SHU39 | -0.019 |
| SHU9 | SHU42 | -0.019 |
| SHU37 | SHU78 | -0.019 |
| SHU28 | SHU81 | -0.019 |
| SHU22 | SHU41 | -0.019 |
| SHU30 | SHU70 | -0.019 |
| SHU49 | SHU70 | -0.019 |
| SHU20 | SHU82 | -0.019 |
| SHU18 | SHU54 | -0.019 |
| SHU48 | SHU54 | -0.019 |
| SHU9 | SHU61 | -0.019 |
| SHU39 | SHU68 | -0.019 |
| SHU27 | SHU55 | -0.019 |
| SHU44 | SHU47 | -0.019 |
| SHU72 | SHU78 | -0.019 |
| SHU9 | SHU65 | -0.019 |
| SHU40 | SHU75 | -0.019 |
| SHU23 | SHU75 | -0.019 |
| SHU51 | SHU79 | -0.019 |
| SHU9 | SHU49 | -0.019 |
| SHU39 | SHU49 | -0.019 |
| SHU28 | SHU73 | -0.019 |
| SHU30 | SHU51 | -0.019 |
| SHU49 | SHU61 | -0.019 |
| SHU49 | SHU79 | -0.019 |
| SHU56 | SHU68 | -0.019 |
| SHU5 | SHU82 | -0.019 |
| SHU9 | SHU68 | -0.019 |
| SHU28 | SHU65 | -0.018 |
| SHU28 | SHU43 | -0.018 |
| SHU36 | SHU75 | -0.018 |
| SHU40 | SHU73 | -0.018 |
| SHU62 | SHU67 | -0.018 |
| SHU50 | SHU79 | -0.018 |
| SHU3 | SHU75 | -0.018 |
| SHU1 | SHU54 | -0.018 |
| SHU49 | SHU60 | -0.018 |
| SHU41 | SHU71 | -0.018 |
| SHU40 | SHU81 | -0.018 |
| SHU12 | SHU69 | -0.018 |
| SHU45 | SHU74 | -0.018 |
| SHU39 | SHU61 | -0.018 |
| SHU65 | SHU66 | -0.018 |
| SHU66 | SHU81 | -0.018 |
| SHU77 | SHU81 | -0.018 |
| SHU12 | SHU22 | -0.018 |
| SHU43 | SHU69 | -0.018 |
| SHU59 | SHU75 | -0.018 |
| SHU52 | SHU78 | -0.018 |
| SHU9 | SHU37 | -0.018 |
| SHU3 | SHU37 | -0.018 |
| SHU33 | SHU41 | -0.018 |
| SHU36 | SHU68 | -0.018 |
| SHU71 | SHU78 | -0.018 |
| SHU68 | SHU80 | -0.018 |
| SHU40 | SHU54 | -0.017 |
| SHU5 | SHU12 | -0.017 |
| SHU12 | SHU43 | -0.017 |
| SHU22 | SHU46 | -0.017 |
| SHU9 | SHU74 | -0.017 |
| SHU25 | SHU79 | -0.017 |
| SHU8 | SHU46 | -0.017 |
| SHU36 | SHU72 | -0.017 |
| SHU22 | SHU75 | -0.017 |
| SHU68 | SHU81 | -0.017 |
| SHU47 | SHU49 | -0.017 |
| SHU37 | SHU75 | -0.017 |
| SHU39 | SHU42 | -0.017 |
| SHU41 | SHU81 | -0.017 |
| SHU9 | SHU54 | -0.017 |
| SHU61 | SHU77 | -0.017 |
| SHU26 | SHU34 | -0.017 |
| SHU52 | SHU75 | -0.017 |
| SHU6 | SHU33 | -0.017 |
| SHU15 | SHU47 | -0.017 |
| SHU15 | SHU49 | -0.017 |
| SHU56 | SHU62 | -0.017 |
| SHU22 | SHU34 | -0.017 |
| SHU79 | SHU81 | -0.017 |
| SHU41 | SHU77 | -0.017 |
| SHU5 | SHU60 | -0.016 |
| SHU73 | SHU81 | -0.016 |
| SHU33 | SHU62 | -0.016 |
| SHU70 | SHU75 | -0.016 |
| SHU17 | SHU77 | -0.016 |
| SHU24 | SHU77 | -0.016 |
| SHU44 | SHU64 | -0.016 |
| SHU2 | SHU42 | -0.016 |
| SHU10 | SHU45 | -0.016 |
| SHU54 | SHU64 | -0.016 |
| SHU60 | SHU62 | -0.016 |
| SHU21 | SHU22 | -0.016 |
| SHU36 | SHU82 | -0.016 |
| SHU50 | SHU51 | -0.016 |
| SHU70 | SHU81 | -0.016 |
| SHU13 | SHU26 | -0.016 |
| SHU9 | SHU34 | -0.016 |
| SHU33 | SHU57 | -0.016 |
| SHU26 | SHU38 | -0.016 |
| SHU41 | SHU45 | -0.016 |
| SHU49 | SHU76 | -0.016 |
| SHU4 | SHU42 | -0.016 |
| SHU60 | SHU81 | -0.016 |
| SHU2 | SHU20 | -0.016 |
| SHU2 | SHU79 | -0.016 |
| SHU39 | SHU41 | -0.015 |
| SHU49 | SHU80 | -0.015 |
| SHU9 | SHU41 | -0.015 |
| SHU22 | SHU59 | -0.015 |
| SHU10 | SHU55 | -0.015 |
| SHU4 | SHU67 | -0.015 |
| SHU49 | SHU62 | -0.015 |
| SHU37 | SHU39 | -0.015 |
| SHU10 | SHU43 | -0.015 |
| SHU5 | SHU11 | -0.015 |
| SHU11 | SHU60 | -0.015 |
| SHU3 | SHU9 | -0.015 |
| SHU56 | SHU81 | -0.015 |
| SHU30 | SHU81 | -0.015 |
| SHU14 | SHU49 | -0.015 |
| SHU47 | SHU50 | -0.015 |
| SHU6 | SHU30 | -0.015 |
| SHU81 | SHU82 | -0.015 |
| SHU46 | SHU74 | -0.015 |
| SHU9 | SHU79 | -0.015 |
| SHU10 | SHU80 | -0.015 |
| SHU25 | SHU71 | -0.015 |
| SHU14 | SHU56 | -0.015 |
| SHU68 | SHU77 | -0.015 |
| SHU30 | SHU36 | -0.015 |
| SHU25 | SHU69 | -0.015 |
| SHU36 | SHU49 | -0.015 |
| SHU4 | SHU46 | -0.014 |
| SHU33 | SHU51 | -0.014 |
| SHU16 | SHU77 | -0.014 |
| SHU80 | SHU81 | -0.014 |
| SHU72 | SHU77 | -0.014 |
| SHU16 | SHU54 | -0.014 |
| SHU32 | SHU49 | -0.014 |
| SHU49 | SHU66 | -0.014 |
| SHU5 | SHU64 | -0.014 |
| SHU15 | SHU28 | -0.014 |
| SHU12 | SHU28 | -0.014 |
| SHU43 | SHU52 | -0.014 |
| SHU4 | SHU79 | -0.014 |
| SHU59 | SHU62 | -0.014 |
| SHU54 | SHU59 | -0.013 |
| SHU6 | SHU37 | -0.013 |
| SHU39 | SHU79 | -0.013 |
| SHU5 | SHU49 | -0.013 |
| SHU17 | SHU70 | -0.013 |
| SHU24 | SHU70 | -0.013 |
| SHU20 | SHU58 | -0.013 |
| SHU30 | SHU82 | -0.013 |
| SHU15 | SHU38 | -0.013 |
| SHU41 | SHU56 | -0.013 |
| SHU26 | SHU49 | -0.013 |
| SHU4 | SHU78 | -0.013 |
| SHU41 | SHU82 | -0.013 |
| SHU42 | SHU77 | -0.013 |
| SHU18 | SHU20 | -0.013 |
| SHU20 | SHU48 | -0.013 |
| SHU1 | SHU57 | -0.013 |
| SHU4 | SHU75 | -0.013 |
| SHU9 | SHU71 | -0.013 |
| SHU22 | SHU36 | -0.013 |
| SHU9 | SHU22 | -0.013 |
| SHU26 | SHU36 | -0.013 |
| SHU3 | SHU67 | -0.013 |
| SHU30 | SHU75 | -0.013 |
| SHU26 | SHU47 | -0.013 |
| SHU39 | SHU74 | -0.012 |
| SHU37 | SHU71 | -0.012 |
| SHU37 | SHU59 | -0.012 |
| SHU22 | SHU29 | -0.012 |
| SHU15 | SHU36 | -0.012 |
| SHU32 | SHU81 | -0.012 |
| SHU41 | SHU80 | -0.012 |
| SHU15 | SHU32 | -0.012 |
| SHU28 | SHU38 | -0.012 |
| SHU47 | SHU60 | -0.012 |
| SHU10 | SHU46 | -0.012 |
| SHU8 | SHU33 | -0.012 |
| SHU22 | SHU72 | -0.012 |
| SHU31 | SHU56 | -0.012 |
| SHU45 | SHU71 | -0.012 |
| SHU3 | SHU70 | -0.012 |
| SHU28 | SHU46 | -0.012 |
| SHU15 | SHU42 | -0.012 |
| SHU28 | SHU58 | -0.012 |
| SHU28 | SHU51 | -0.012 |
| SHU60 | SHU78 | -0.012 |
| SHU31 | SHU49 | -0.012 |
| SHU37 | SHU62 | -0.012 |
| SHU38 | SHU75 | -0.012 |
| SHU16 | SHU76 | -0.012 |
| SHU62 | SHU73 | -0.011 |
| SHU51 | SHU75 | -0.011 |
| SHU39 | SHU71 | -0.011 |
| SHU12 | SHU74 | -0.011 |
| SHU11 | SHU28 | -0.011 |
| SHU36 | SHU46 | -0.011 |
| SHU33 | SHU74 | -0.011 |
| SHU70 | SHU72 | -0.011 |
| SHU67 | SHU78 | -0.011 |
| SHU67 | SHU70 | -0.011 |
| SHU56 | SHU61 | -0.011 |
| SHU42 | SHU69 | -0.011 |
| SHU46 | SHU52 | -0.011 |
| SHU3 | SHU56 | -0.011 |
| SHU77 | SHU79 | -0.011 |
| SHU20 | SHU62 | -0.011 |
| SHU22 | SHU49 | -0.011 |
| SHU22 | SHU25 | -0.011 |
| SHU22 | SHU39 | -0.010 |
| SHU61 | SHU80 | -0.010 |
| SHU52 | SHU70 | -0.010 |
| SHU69 | SHU79 | -0.010 |
| SHU10 | SHU11 | -0.010 |
| SHU17 | SHU31 | -0.010 |
| SHU24 | SHU31 | -0.010 |
| SHU32 | SHU65 | -0.010 |
| SHU6 | SHU43 | -0.010 |
| SHU11 | SHU44 | -0.010 |
| SHU6 | SHU78 | -0.010 |
| SHU30 | SHU71 | -0.010 |
| SHU26 | SHU65 | -0.010 |
| SHU34 | SHU45 | -0.010 |
| SHU14 | SHU73 | -0.009 |
| SHU46 | SHU80 | -0.009 |
| SHU27 | SHU28 | -0.009 |
| SHU26 | SHU54 | -0.009 |
| SHU15 | SHU61 | -0.009 |
| SHU15 | SHU79 | -0.009 |
| SHU17 | SHU20 | -0.009 |
| SHU20 | SHU24 | -0.009 |
| SHU31 | SHU72 | -0.009 |
| SHU23 | SHU30 | -0.009 |
| SHU41 | SHU79 | -0.009 |
| SHU9 | SHU25 | -0.009 |
| SHU49 | SHU59 | -0.009 |
| SHU6 | SHU75 | -0.009 |
| SHU1 | SHU33 | -0.009 |
| SHU9 | SHU26 | -0.009 |
| SHU78 | SHU82 | -0.009 |
| SHU37 | SHU49 | -0.009 |
| SHU49 | SHU81 | -0.008 |
| SHU8 | SHU42 | -0.008 |
| SHU15 | SHU37 | -0.008 |
| SHU57 | SHU66 | -0.008 |
| SHU4 | SHU58 | -0.008 |
| SHU9 | SHU28 | -0.008 |
| SHU43 | SHU49 | -0.008 |
| SHU30 | SHU49 | -0.008 |
| SHU49 | SHU77 | -0.008 |
| SHU33 | SHU36 | -0.008 |
| SHU8 | SHU79 | -0.008 |
| SHU15 | SHU54 | -0.008 |
| SHU75 | SHU82 | -0.008 |
| SHU44 | SHU53 | -0.008 |
| SHU34 | SHU39 | -0.008 |
| SHU43 | SHU64 | -0.008 |
| SHU20 | SHU51 | -0.008 |
| SHU63 | SHU77 | -0.008 |
| SHU25 | SHU74 | -0.008 |
| SHU33 | SHU45 | -0.007 |
| SHU31 | SHU73 | -0.007 |
| SHU53 | SHU56 | -0.007 |
| SHU26 | SHU39 | -0.007 |
| SHU44 | SHU75 | -0.007 |
| SHU6 | SHU46 | -0.007 |
| SHU22 | SHU45 | -0.007 |
| SHU2 | SHU71 | -0.007 |
| SHU25 | SHU33 | -0.007 |
| SHU3 | SHU49 | -0.007 |
| SHU42 | SHU54 | -0.007 |
| SHU7 | SHU39 | -0.007 |
| SHU62 | SHU71 | -0.007 |
| SHU58 | SHU74 | -0.007 |
| SHU42 | SHU60 | -0.007 |
| SHU28 | SHU39 | -0.007 |
| SHU23 | SHU43 | -0.007 |
| SHU18 | SHU74 | -0.007 |
| SHU48 | SHU74 | -0.007 |
| SHU28 | SHU75 | -0.007 |
| SHU28 | SHU29 | -0.007 |
| SHU1 | SHU19 | -0.007 |
| SHU11 | SHU17 | -0.007 |
| SHU11 | SHU24 | -0.007 |
| SHU25 | SHU45 | -0.007 |
| SHU43 | SHU56 | -0.007 |
| SHU33 | SHU80 | -0.006 |
| SHU3 | SHU60 | -0.006 |
| SHU23 | SHU66 | -0.006 |
| SHU2 | SHU62 | -0.006 |
| SHU28 | SHU53 | -0.006 |
| SHU3 | SHU26 | -0.006 |
| SHU20 | SHU46 | -0.006 |
| SHU11 | SHU72 | -0.006 |
| SHU11 | SHU37 | -0.006 |
| SHU71 | SHU82 | -0.006 |
| SHU22 | SHU77 | -0.006 |
| SHU27 | SHU44 | -0.006 |
| SHU11 | SHU52 | -0.006 |
| SHU38 | SHU44 | -0.006 |
| SHU28 | SHU50 | -0.006 |
| SHU1 | SHU42 | -0.006 |
| SHU38 | SHU43 | -0.006 |
| SHU30 | SHU60 | -0.006 |
| SHU49 | SHU78 | -0.005 |
| SHU9 | SHU82 | -0.005 |
| SHU60 | SHU79 | -0.005 |
| SHU10 | SHU67 | -0.005 |
| SHU54 | SHU79 | -0.005 |
| SHU23 | SHU73 | -0.005 |
| SHU6 | SHU70 | -0.005 |
| SHU46 | SHU64 | -0.005 |
| SHU1 | SHU34 | -0.005 |
| SHU1 | SHU3 | -0.005 |
| SHU11 | SHU36 | -0.005 |
| SHU75 | SHU78 | -0.005 |
| SHU26 | SHU70 | -0.005 |
| SHU1 | SHU61 | -0.005 |
| SHU1 | SHU79 | -0.005 |
| SHU17 | SHU74 | -0.005 |
| SHU24 | SHU74 | -0.005 |
| SHU28 | SHU59 | -0.005 |
| SHU9 | SHU77 | -0.005 |
| SHU23 | SHU54 | -0.005 |
| SHU42 | SHU45 | -0.005 |
| SHU26 | SHU64 | -0.004 |
| SHU25 | SHU39 | -0.004 |
| SHU49 | SHU75 | -0.004 |
| SHU22 | SHU78 | -0.004 |
| SHU44 | SHU51 | -0.004 |
| SHU37 | SHU63 | -0.004 |
| SHU10 | SHU34 | -0.004 |
| SHU62 | SHU69 | -0.004 |
| SHU27 | SHU56 | -0.004 |
| SHU36 | SHU47 | -0.004 |
| SHU30 | SHU47 | -0.004 |
| SHU57 | SHU69 | -0.004 |
| SHU6 | SHU11 | -0.004 |
| SHU3 | SHU54 | -0.004 |
| SHU28 | SHU66 | -0.004 |
| SHU32 | SHU64 | -0.004 |
| SHU42 | SHU56 | -0.004 |
| SHU45 | SHU79 | -0.004 |
| SHU42 | SHU82 | -0.004 |
| SHU38 | SHU66 | -0.004 |
| SHU42 | SHU80 | -0.004 |
| SHU26 | SHU57 | -0.004 |
| SHU3 | SHU82 | -0.004 |
| SHU8 | SHU71 | -0.004 |
| SHU9 | SHU30 | -0.004 |
| SHU56 | SHU79 | -0.003 |
| SHU79 | SHU82 | -0.003 |
| SHU79 | SHU80 | -0.003 |
| SHU23 | SHU27 | -0.003 |
| SHU38 | SHU73 | -0.003 |
| SHU56 | SHU59 | -0.003 |
| SHU54 | SHU73 | -0.003 |
| SHU27 | SHU38 | -0.003 |
| SHU13 | SHU71 | -0.003 |
| SHU38 | SHU54 | -0.003 |
| SHU59 | SHU71 | -0.003 |
| SHU28 | SHU33 | -0.003 |
| SHU52 | SHU57 | -0.003 |
| SHU17 | SHU67 | -0.002 |
| SHU24 | SHU67 | -0.002 |
| SHU54 | SHU65 | -0.002 |
| SHU45 | SHU77 | -0.002 |
| SHU66 | SHU73 | -0.002 |
| SHU22 | SHU74 | -0.002 |
| SHU67 | SHU72 | -0.002 |
| SHU64 | SHU73 | -0.002 |
| SHU52 | SHU67 | -0.002 |
| SHU21 | SHU42 | -0.002 |
| SHU42 | SHU78 | -0.002 |
| SHU1 | SHU36 | -0.002 |
| SHU11 | SHU29 | -0.002 |
| SHU21 | SHU79 | -0.002 |
| SHU30 | SHU42 | -0.002 |
| SHU20 | SHU33 | -0.002 |
| SHU30 | SHU67 | -0.002 |
| SHU78 | SHU79 | -0.002 |
| SHU11 | SHU31 | -0.002 |
| SHU56 | SHU71 | -0.002 |
| SHU39 | SHU77 | -0.002 |
| SHU30 | SHU79 | -0.001 |
| SHU3 | SHU46 | -0.001 |
| SHU71 | SHU80 | -0.001 |
| SHU42 | SHU71 | -0.001 |
| SHU11 | SHU42 | -0.001 |
| SHU30 | SHU39 | -0.001 |
| SHU22 | SHU33 | -0.001 |
| SHU19 | SHU28 | -0.001 |
| SHU39 | SHU82 | -0.001 |
| SHU11 | SHU79 | -0.001 |
| SHU62 | SHU82 | -0.001 |
| SHU1 | SHU13 | -0.001 |
| SHU6 | SHU67 | -0.001 |
| SHU71 | SHU79 | -0.001 |
| SHU49 | SHU56 | -0.001 |
| SHU47 | SHU79 | -0.001 |
| SHU47 | SHU67 | -0.001 |
| SHU26 | SHU66 | -0.001 |
| SHU28 | SHU57 | -0.001 |
| SHU20 | SHU67 | 0.000 |
| SHU32 | SHU66 | 0.000 |
| SHU22 | SHU63 | 0.000 |
| SHU33 | SHU43 | 0.000 |
| SHU53 | SHU72 | 0.000 |
| SHU17 | SHU53 | 0.000 |
| SHU24 | SHU53 | 0.000 |
| SHU63 | SHU76 | 0.000 |
| SHU67 | SHU79 | 0.000 |
| SHU42 | SHU67 | 0.000 |
| SHU9 | SHU44 | 0.000 |
| SHU37 | SHU80 | 0.000 |
| SHU25 | SHU37 | 0.000 |
| SHU57 | SHU79 | 0.000 |
| SHU64 | SHU79 | 0.000 |
| SHU61 | SHU64 | 0.000 |
| SHU42 | SHU57 | 0.000 |
| SHU38 | SHU57 | 0.001 |
| SHU64 | SHU67 | 0.001 |
| SHU29 | SHU79 | 0.001 |
| SHU13 | SHU37 | 0.001 |
| SHU15 | SHU26 | 0.001 |
| SHU29 | SHU42 | 0.001 |
| SHU13 | SHU25 | 0.001 |
| SHU55 | SHU64 | 0.001 |
| SHU8 | SHU37 | 0.001 |
| SHU13 | SHU34 | 0.001 |
| SHU23 | SHU57 | 0.001 |
| SHU18 | SHU80 | 0.001 |
| SHU48 | SHU80 | 0.001 |
| SHU4 | SHU59 | 0.001 |
| SHU58 | SHU80 | 0.001 |
| SHU26 | SHU59 | 0.001 |
| SHU32 | SHU44 | 0.001 |
| SHU59 | SHU76 | 0.001 |
| SHU66 | SHU71 | 0.001 |
| SHU44 | SHU66 | 0.001 |
| SHU64 | SHU75 | 0.001 |
| SHU8 | SHU18 | 0.001 |
| SHU8 | SHU48 | 0.001 |
| SHU8 | SHU58 | 0.001 |
| SHU10 | SHU59 | 0.001 |
| SHU47 | SHU75 | 0.001 |
| SHU60 | SHU67 | 0.001 |
| SHU46 | SHU77 | 0.002 |
| SHU4 | SHU32 | 0.002 |
| SHU16 | SHU67 | 0.002 |
| SHU15 | SHU52 | 0.002 |
| SHU9 | SHU32 | 0.002 |
| SHU9 | SHU50 | 0.002 |
| SHU26 | SHU28 | 0.002 |
| SHU6 | SHU49 | 0.002 |
| SHU9 | SHU56 | 0.002 |
| SHU26 | SHU81 | 0.002 |
| SHU28 | SHU30 | 0.002 |
| SHU44 | SHU50 | 0.002 |
| SHU32 | SHU59 | 0.002 |
| SHU66 | SHU74 | 0.003 |
| SHU76 | SHU80 | 0.003 |
| SHU10 | SHU77 | 0.003 |
| SHU6 | SHU82 | 0.003 |
| SHU21 | SHU62 | 0.003 |
| SHU30 | SHU62 | 0.003 |
| SHU41 | SHU73 | 0.003 |
| SHU31 | SHU82 | 0.003 |
| SHU15 | SHU68 | 0.003 |
| SHU47 | SHU78 | 0.003 |
| SHU22 | SHU71 | 0.003 |
| SHU22 | SHU42 | 0.003 |
| SHU39 | SHU44 | 0.003 |
| SHU22 | SHU80 | 0.003 |
| SHU60 | SHU70 | 0.003 |
| SHU54 | SHU61 | 0.004 |
| SHU14 | SHU82 | 0.004 |
| SHU22 | SHU56 | 0.004 |
| SHU30 | SHU44 | 0.004 |
| SHU33 | SHU77 | 0.004 |
| SHU23 | SHU32 | 0.004 |
| SHU49 | SHU52 | 0.004 |
| SHU13 | SHU76 | 0.004 |
| SHU28 | SHU80 | 0.004 |
| SHU49 | SHU72 | 0.004 |
| SHU29 | SHU60 | 0.004 |
| SHU22 | SHU30 | 0.004 |
| SHU6 | SHU26 | 0.004 |
| SHU33 | SHU49 | 0.004 |
| SHU66 | SHU67 | 0.004 |
| SHU33 | SHU64 | 0.004 |
| SHU8 | SHU76 | 0.004 |
| SHU10 | SHU49 | 0.004 |
| SHU52 | SHU82 | 0.004 |
| SHU67 | SHU73 | 0.005 |
| SHU26 | SHU45 | 0.005 |
| SHU17 | SHU49 | 0.005 |
| SHU24 | SHU49 | 0.005 |
| SHU29 | SHU62 | 0.005 |
| SHU3 | SHU5 | 0.005 |
| SHU20 | SHU47 | 0.005 |
| SHU1 | SHU53 | 0.005 |
| SHU28 | SHU45 | 0.005 |
| SHU14 | SHU15 | 0.005 |
| SHU13 | SHU77 | 0.005 |
| SHU25 | SHU77 | 0.005 |
| SHU1 | SHU20 | 0.005 |
| SHU68 | SHU70 | 0.005 |
| SHU1 | SHU29 | 0.005 |
| SHU26 | SHU67 | 0.005 |
| SHU68 | SHU73 | 0.005 |
| SHU15 | SHU21 | 0.006 |
| SHU41 | SHU57 | 0.006 |
| SHU27 | SHU60 | 0.006 |
| SHU9 | SHU81 | 0.006 |
| SHU39 | SHU50 | 0.006 |
| SHU51 | SHU53 | 0.006 |
| SHU1 | SHU31 | 0.006 |
| SHU16 | SHU53 | 0.006 |
| SHU23 | SHU50 | 0.006 |
| SHU15 | SHU60 | 0.006 |
| SHU32 | SHU39 | 0.006 |
| SHU37 | SHU51 | 0.007 |
| SHU25 | SHU82 | 0.007 |
| SHU12 | SHU30 | 0.007 |
| SHU39 | SHU56 | 0.007 |
| SHU44 | SHU45 | 0.007 |
| SHU15 | SHU16 | 0.007 |
| SHU6 | SHU27 | 0.007 |
| SHU55 | SHU62 | 0.007 |
| SHU13 | SHU82 | 0.007 |
| SHU6 | SHU69 | 0.007 |
| SHU20 | SHU64 | 0.007 |
| SHU41 | SHU60 | 0.007 |
| SHU27 | SHU64 | 0.007 |
| SHU44 | SHU77 | 0.007 |
| SHU57 | SHU70 | 0.007 |
| SHU62 | SHU75 | 0.007 |
| SHU31 | SHU63 | 0.007 |
| SHU10 | SHU72 | 0.007 |
| SHU37 | SHU77 | 0.007 |
| SHU35 | SHU49 | 0.007 |
| SHU1 | SHU14 | 0.007 |
| SHU46 | SHU76 | 0.008 |
| SHU60 | SHU73 | 0.008 |
| SHU22 | SHU62 | 0.008 |
| SHU56 | SHU72 | 0.008 |
| SHU3 | SHU71 | 0.008 |
| SHU8 | SHU22 | 0.008 |
| SHU21 | SHU49 | 0.008 |
| SHU4 | SHU80 | 0.008 |
| SHU5 | SHU36 | 0.008 |
| SHU59 | SHU61 | 0.008 |
| SHU11 | SHU38 | 0.008 |
| SHU26 | SHU72 | 0.008 |
| SHU6 | SHU58 | 0.008 |
| SHU22 | SHU37 | 0.008 |
| SHU5 | SHU33 | 0.008 |
| SHU57 | SHU65 | 0.008 |
| SHU12 | SHU77 | 0.008 |
| SHU27 | SHU52 | 0.008 |
| SHU30 | SHU78 | 0.009 |
| SHU9 | SHU38 | 0.009 |
| SHU35 | SHU82 | 0.009 |
| SHU9 | SHU51 | 0.009 |
| SHU27 | SHU72 | 0.009 |
| SHU31 | SHU64 | 0.009 |
| SHU69 | SHU72 | 0.009 |
| SHU7 | SHU31 | 0.009 |
| SHU73 | SHU75 | 0.009 |
| SHU15 | SHU57 | 0.009 |
| SHU17 | SHU56 | 0.009 |
| SHU24 | SHU56 | 0.009 |
| SHU58 | SHU64 | 0.009 |
| SHU39 | SHU81 | 0.009 |
| SHU62 | SHU79 | 0.009 |
| SHU21 | SHU82 | 0.009 |
| SHU17 | SHU27 | 0.009 |
| SHU24 | SHU27 | 0.009 |
| SHU7 | SHU14 | 0.009 |
| SHU16 | SHU49 | 0.009 |
| SHU29 | SHU49 | 0.009 |
| SHU17 | SHU69 | 0.009 |
| SHU24 | SHU69 | 0.009 |
| SHU17 | SHU26 | 0.010 |
| SHU24 | SHU26 | 0.010 |
| SHU8 | SHU26 | 0.010 |
| SHU65 | SHU79 | 0.010 |
| SHU33 | SHU66 | 0.010 |
| SHU2 | SHU6 | 0.010 |
| SHU66 | SHU77 | 0.010 |
| SHU12 | SHU20 | 0.010 |
| SHU8 | SHU28 | 0.010 |
| SHU52 | SHU58 | 0.010 |
| SHU64 | SHU65 | 0.010 |
| SHU54 | SHU70 | 0.010 |
| SHU38 | SHU59 | 0.010 |
| SHU1 | SHU75 | 0.010 |
| SHU20 | SHU38 | 0.010 |
| SHU6 | SHU7 | 0.010 |
| SHU10 | SHU18 | 0.011 |
| SHU10 | SHU48 | 0.011 |
| SHU21 | SHU46 | 0.011 |
| SHU12 | SHU82 | 0.011 |
| SHU10 | SHU58 | 0.011 |
| SHU33 | SHU76 | 0.011 |
| SHU2 | SHU9 | 0.011 |
| SHU14 | SHU64 | 0.011 |
| SHU29 | SHU82 | 0.011 |
| SHU5 | SHU75 | 0.011 |
| SHU74 | SHU80 | 0.011 |
| SHU45 | SHU82 | 0.011 |
| SHU44 | SHU74 | 0.011 |
| SHU66 | SHU68 | 0.011 |
| SHU45 | SHU56 | 0.011 |
| SHU42 | SHU62 | 0.011 |
| SHU22 | SHU28 | 0.011 |
| SHU2 | SHU52 | 0.012 |
| SHU38 | SHU65 | 0.012 |
| SHU43 | SHU82 | 0.012 |
| SHU34 | SHU46 | 0.012 |
| SHU7 | SHU52 | 0.012 |
| SHU42 | SHU65 | 0.012 |
| SHU53 | SHU73 | 0.012 |
| SHU27 | SHU33 | 0.012 |
| SHU46 | SHU49 | 0.012 |
| SHU38 | SHU55 | 0.013 |
| SHU68 | SHU78 | 0.013 |
| SHU37 | SHU46 | 0.013 |
| SHU9 | SHU40 | 0.013 |
| SHU38 | SHU81 | 0.013 |
| SHU1 | SHU59 | 0.013 |
| SHU52 | SHU66 | 0.013 |
| SHU33 | SHU47 | 0.013 |
| SHU39 | SHU51 | 0.013 |
| SHU3 | SHU44 | 0.013 |
| SHU30 | SHU80 | 0.013 |
| SHU38 | SHU39 | 0.014 |
| SHU29 | SHU73 | 0.014 |
| SHU20 | SHU23 | 0.014 |
| SHU38 | SHU79 | 0.014 |
| SHU44 | SHU46 | 0.014 |
| SHU49 | SHU82 | 0.014 |
| SHU39 | SHU40 | 0.015 |
| SHU16 | SHU56 | 0.015 |
| SHU4 | SHU81 | 0.015 |
| SHU26 | SHU51 | 0.015 |
| SHU23 | SHU59 | 0.015 |
| SHU18 | SHU33 | 0.015 |
| SHU33 | SHU48 | 0.015 |
| SHU3 | SHU6 | 0.015 |
| SHU26 | SHU32 | 0.015 |
| SHU66 | SHU76 | 0.015 |
| SHU33 | SHU58 | 0.015 |
| SHU31 | SHU51 | 0.015 |
| SHU33 | SHU63 | 0.015 |
| SHU76 | SHU77 | 0.016 |
| SHU66 | SHU78 | 0.016 |
| SHU38 | SHU67 | 0.016 |
| SHU59 | SHU82 | 0.016 |
| SHU6 | SHU28 | 0.016 |
| SHU21 | SHU69 | 0.016 |
| SHU1 | SHU22 | 0.016 |
| SHU1 | SHU37 | 0.016 |
| SHU25 | SHU70 | 0.017 |
| SHU40 | SHU70 | 0.017 |
| SHU77 | SHU80 | 0.017 |
| SHU30 | SHU45 | 0.017 |
| SHU22 | SHU82 | 0.017 |
| SHU25 | SHU73 | 0.017 |
| SHU34 | SHU76 | 0.017 |
| SHU22 | SHU27 | 0.017 |
| SHU43 | SHU62 | 0.017 |
| SHU19 | SHU26 | 0.017 |
| SHU10 | SHU74 | 0.017 |
| SHU22 | SHU76 | 0.017 |
| SHU19 | SHU73 | 0.017 |
| SHU23 | SHU55 | 0.017 |
| SHU13 | SHU74 | 0.017 |
| SHU15 | SHU55 | 0.018 |
| SHU16 | SHU27 | 0.018 |
| SHU4 | SHU50 | 0.018 |
| SHU16 | SHU69 | 0.018 |
| SHU37 | SHU74 | 0.018 |
| SHU35 | SHU66 | 0.018 |
| SHU18 | SHU35 | 0.018 |
| SHU35 | SHU48 | 0.018 |
| SHU46 | SHU56 | 0.018 |
| SHU15 | SHU78 | 0.018 |
| SHU5 | SHU34 | 0.018 |
| SHU3 | SHU58 | 0.018 |
| SHU38 | SHU46 | 0.018 |
| SHU23 | SHU65 | 0.018 |
| SHU25 | SHU80 | 0.018 |
| SHU19 | SHU33 | 0.019 |
| SHU30 | SHU50 | 0.019 |
| SHU37 | SHU72 | 0.019 |
| SHU35 | SHU63 | 0.019 |
| SHU59 | SHU74 | 0.019 |
| SHU19 | SHU38 | 0.019 |
| SHU14 | SHU51 | 0.019 |
| SHU21 | SHU58 | 0.019 |
| SHU6 | SHU44 | 0.019 |
| SHU25 | SHU56 | 0.019 |
| SHU7 | SHU71 | 0.019 |
| SHU33 | SHU56 | 0.019 |
| SHU51 | SHU81 | 0.019 |
| SHU59 | SHU65 | 0.019 |
| SHU7 | SHU13 | 0.019 |
| SHU53 | SHU69 | 0.020 |
| SHU15 | SHU64 | 0.020 |
| SHU71 | SHU77 | 0.020 |
| SHU13 | SHU32 | 0.020 |
| SHU19 | SHU32 | 0.020 |
| SHU29 | SHU69 | 0.020 |
| SHU14 | SHU66 | 0.020 |
| SHU80 | SHU82 | 0.020 |
| SHU53 | SHU82 | 0.020 |
| SHU37 | SHU56 | 0.021 |
| SHU3 | SHU65 | 0.021 |
| SHU23 | SHU79 | 0.021 |
| SHU11 | SHU32 | 0.021 |
| SHU30 | SHU63 | 0.021 |
| SHU22 | SHU44 | 0.021 |
| SHU5 | SHU65 | 0.021 |
| SHU43 | SHU44 | 0.021 |
| SHU8 | SHU74 | 0.021 |
| SHU1 | SHU46 | 0.021 |
| SHU23 | SHU81 | 0.021 |
| SHU2 | SHU35 | 0.021 |
| SHU17 | SHU37 | 0.021 |
| SHU24 | SHU37 | 0.021 |
| SHU69 | SHU74 | 0.021 |
| SHU37 | SHU47 | 0.021 |
| SHU2 | SHU21 | 0.022 |
| SHU5 | SHU13 | 0.022 |
| SHU62 | SHU74 | 0.022 |
| SHU10 | SHU71 | 0.022 |
| SHU1 | SHU51 | 0.022 |
| SHU28 | SHU64 | 0.022 |
| SHU2 | SHU3 | 0.022 |
| SHU3 | SHU59 | 0.022 |
| SHU7 | SHU35 | 0.022 |
| SHU44 | SHU80 | 0.022 |
| SHU1 | SHU28 | 0.022 |
| SHU45 | SHU50 | 0.022 |
| SHU19 | SHU52 | 0.023 |
| SHU42 | SHU61 | 0.023 |
| SHU5 | SHU20 | 0.023 |
| SHU23 | SHU67 | 0.023 |
| SHU38 | SHU45 | 0.023 |
| SHU18 | SHU34 | 0.023 |
| SHU34 | SHU48 | 0.023 |
| SHU12 | SHU44 | 0.023 |
| SHU74 | SHU82 | 0.023 |
| SHU32 | SHU33 | 0.023 |
| SHU23 | SHU42 | 0.023 |
| SHU59 | SHU77 | 0.023 |
| SHU18 | SHU29 | 0.023 |
| SHU29 | SHU48 | 0.023 |
| SHU32 | SHU80 | 0.023 |
| SHU67 | SHU76 | 0.023 |
| SHU36 | SHU43 | 0.023 |
| SHU28 | SHU40 | 0.023 |
| SHU34 | SHU58 | 0.023 |
| SHU23 | SHU46 | 0.024 |
| SHU31 | SHU67 | 0.024 |
| SHU13 | SHU50 | 0.024 |
| SHU31 | SHU46 | 0.025 |
| SHU12 | SHU32 | 0.025 |
| SHU7 | SHU25 | 0.025 |
| SHU20 | SHU36 | 0.025 |
| SHU64 | SHU66 | 0.025 |
| SHU13 | SHU56 | 0.025 |
| SHU69 | SHU80 | 0.025 |
| SHU77 | SHU82 | 0.026 |
| SHU19 | SHU23 | 0.026 |
| SHU34 | SHU36 | 0.026 |
| SHU1 | SHU44 | 0.026 |
| SHU72 | SHU82 | 0.026 |
| SHU43 | SHU63 | 0.026 |
| SHU15 | SHU66 | 0.026 |
| SHU7 | SHU30 | 0.026 |
| SHU19 | SHU70 | 0.026 |
| SHU4 | SHU6 | 0.026 |
| SHU13 | SHU30 | 0.027 |
| SHU1 | SHU38 | 0.027 |
| SHU18 | SHU77 | 0.027 |
| SHU48 | SHU77 | 0.027 |
| SHU12 | SHU47 | 0.027 |
| SHU45 | SHU69 | 0.027 |
| SHU36 | SHU37 | 0.027 |
| SHU2 | SHU29 | 0.027 |
| SHU19 | SHU79 | 0.028 |
| SHU19 | SHU61 | 0.028 |
| SHU58 | SHU77 | 0.028 |
| SHU4 | SHU77 | 0.028 |
| SHU12 | SHU36 | 0.028 |
| SHU5 | SHU31 | 0.028 |
| SHU3 | SHU52 | 0.028 |
| SHU31 | SHU33 | 0.028 |
| SHU10 | SHU56 | 0.028 |
| SHU34 | SHU80 | 0.028 |
| SHU34 | SHU74 | 0.028 |
| SHU11 | SHU34 | 0.028 |
| SHU32 | SHU45 | 0.029 |
| SHU19 | SHU35 | 0.029 |
| SHU50 | SHU80 | 0.029 |
| SHU28 | SHU69 | 0.029 |
| SHU46 | SHU51 | 0.029 |
| SHU35 | SHU36 | 0.029 |
| SHU14 | SHU46 | 0.029 |
| SHU7 | SHU12 | 0.029 |
| SHU62 | SHU72 | 0.029 |
| SHU5 | SHU22 | 0.029 |
| SHU17 | SHU82 | 0.030 |
| SHU24 | SHU82 | 0.030 |
| SHU57 | SHU64 | 0.030 |
| SHU37 | SHU82 | 0.030 |
| SHU46 | SHU75 | 0.030 |
| SHU52 | SHU64 | 0.030 |
| SHU5 | SHU53 | 0.030 |
| SHU59 | SHU79 | 0.030 |
| SHU29 | SHU30 | 0.031 |
| SHU12 | SHU56 | 0.031 |
| SHU55 | SHU68 | 0.031 |
| SHU47 | SHU63 | 0.031 |
| SHU21 | SHU30 | 0.031 |
| SHU5 | SHU37 | 0.031 |
| SHU21 | SHU36 | 0.031 |
| SHU46 | SHU82 | 0.031 |
| SHU7 | SHU43 | 0.031 |
| SHU64 | SHU76 | 0.031 |
| SHU38 | SHU61 | 0.031 |
| SHU18 | SHU37 | 0.031 |
| SHU37 | SHU48 | 0.031 |
| SHU28 | SHU32 | 0.032 |
| SHU54 | SHU60 | 0.032 |
| SHU19 | SHU42 | 0.032 |
| SHU11 | SHU80 | 0.032 |
| SHU17 | SHU76 | 0.032 |
| SHU24 | SHU76 | 0.032 |
| SHU56 | SHU63 | 0.032 |
| SHU37 | SHU58 | 0.032 |
| SHU12 | SHU25 | 0.032 |
| SHU32 | SHU52 | 0.032 |
| SHU31 | SHU36 | 0.032 |
| SHU40 | SHU78 | 0.032 |
| SHU5 | SHU29 | 0.033 |
| SHU11 | SHU35 | 0.033 |
| SHU14 | SHU33 | 0.033 |
| SHU2 | SHU39 | 0.034 |
| SHU5 | SHU14 | 0.034 |
| SHU27 | SHU79 | 0.034 |
| SHU5 | SHU28 | 0.034 |
| SHU12 | SHU26 | 0.034 |
| SHU8 | SHU30 | 0.034 |
| SHU71 | SHU74 | 0.034 |
| SHU44 | SHU52 | 0.035 |
| SHU22 | SHU69 | 0.035 |
| SHU28 | SHU41 | 0.035 |
| SHU73 | SHU74 | 0.035 |
| SHU19 | SHU41 | 0.035 |
| SHU53 | SHU75 | 0.035 |
| SHU62 | SHU70 | 0.035 |
| SHU45 | SHU51 | 0.035 |
| SHU56 | SHU77 | 0.035 |
| SHU46 | SHU69 | 0.035 |
| SHU27 | SHU42 | 0.036 |
| SHU1 | SHU64 | 0.036 |
| SHU12 | SHU35 | 0.036 |
| SHU8 | SHU69 | 0.036 |
| SHU27 | SHU67 | 0.036 |
| SHU5 | SHU51 | 0.036 |
| SHU42 | SHU59 | 0.036 |
| SHU10 | SHU28 | 0.036 |
| SHU34 | SHU43 | 0.036 |
| SHU1 | SHU21 | 0.037 |
| SHU4 | SHU9 | 0.037 |
| SHU3 | SHU21 | 0.037 |
| SHU31 | SHU47 | 0.037 |
| SHU56 | SHU74 | 0.037 |
| SHU35 | SHU75 | 0.037 |
| SHU8 | SHU25 | 0.037 |
| SHU12 | SHU21 | 0.037 |
| SHU56 | SHU76 | 0.037 |
| SHU3 | SHU77 | 0.038 |
| SHU7 | SHU47 | 0.038 |
| SHU21 | SHU43 | 0.038 |
| SHU19 | SHU20 | 0.038 |
| SHU17 | SHU28 | 0.038 |
| SHU24 | SHU28 | 0.038 |
| SHU1 | SHU23 | 0.038 |
| SHU9 | SHU80 | 0.038 |
| SHU4 | SHU74 | 0.038 |
| SHU16 | SHU82 | 0.038 |
| SHU1 | SHU4 | 0.038 |
| SHU31 | SHU38 | 0.038 |
| SHU29 | SHU75 | 0.038 |
| SHU15 | SHU33 | 0.038 |
| SHU38 | SHU76 | 0.038 |
| SHU8 | SHU77 | 0.038 |
| SHU5 | SHU44 | 0.039 |
| SHU3 | SHU66 | 0.039 |
| SHU34 | SHU51 | 0.039 |
| SHU45 | SHU81 | 0.039 |
| SHU27 | SHU62 | 0.039 |
| SHU46 | SHU72 | 0.039 |
| SHU55 | SHU67 | 0.039 |
| SHU26 | SHU33 | 0.039 |
| SHU30 | SHU73 | 0.039 |
| SHU2 | SHU45 | 0.039 |
| SHU11 | SHU81 | 0.039 |
| SHU47 | SHU53 | 0.039 |
| SHU34 | SHU77 | 0.039 |
| SHU32 | SHU35 | 0.039 |
| SHU10 | SHU37 | 0.040 |
| SHU12 | SHU33 | 0.040 |
| SHU10 | SHU47 | 0.040 |
| SHU5 | SHU46 | 0.041 |
| SHU11 | SHU27 | 0.041 |
| SHU10 | SHU44 | 0.041 |
| SHU19 | SHU65 | 0.041 |
| SHU13 | SHU81 | 0.041 |
| SHU14 | SHU36 | 0.041 |
| SHU14 | SHU26 | 0.041 |
| SHU7 | SHU45 | 0.041 |
| SHU15 | SHU69 | 0.041 |
| SHU53 | SHU66 | 0.041 |
| SHU63 | SHU78 | 0.042 |
| SHU4 | SHU52 | 0.042 |
| SHU23 | SHU80 | 0.042 |
| SHU1 | SHU27 | 0.042 |
| SHU56 | SHU82 | 0.042 |
| SHU55 | SHU75 | 0.042 |
| SHU13 | SHU38 | 0.042 |
| SHU61 | SHU73 | 0.042 |
| SHU29 | SHU78 | 0.043 |
| SHU1 | SHU16 | 0.043 |
| SHU15 | SHU59 | 0.043 |
| SHU29 | SHU51 | 0.043 |
| SHU13 | SHU51 | 0.043 |
| SHU8 | SHU82 | 0.043 |
| SHU18 | SHU76 | 0.043 |
| SHU48 | SHU76 | 0.043 |
| SHU14 | SHU47 | 0.043 |
| SHU23 | SHU28 | 0.044 |
| SHU17 | SHU46 | 0.044 |
| SHU24 | SHU46 | 0.044 |
| SHU4 | SHU38 | 0.044 |
| SHU21 | SHU78 | 0.044 |
| SHU30 | SHU57 | 0.044 |
| SHU4 | SHU39 | 0.044 |
| SHU58 | SHU76 | 0.044 |
| SHU32 | SHU73 | 0.044 |
| SHU34 | SHU82 | 0.045 |
| SHU27 | SHU49 | 0.045 |
| SHU3 | SHU74 | 0.045 |
| SHU10 | SHU33 | 0.045 |
| SHU29 | SHU66 | 0.045 |
| SHU39 | SHU80 | 0.045 |
| SHU33 | SHU82 | 0.045 |
| SHU41 | SHU78 | 0.046 |
| SHU4 | SHU22 | 0.046 |
| SHU49 | SHU69 | 0.046 |
| SHU69 | SHU77 | 0.046 |
| SHU3 | SHU14 | 0.046 |
| SHU37 | SHU76 | 0.046 |
| SHU64 | SHU69 | 0.046 |
| SHU53 | SHU63 | 0.046 |
| SHU65 | SHU81 | 0.046 |
| SHU4 | SHU47 | 0.047 |
| SHU14 | SHU38 | 0.047 |
| SHU8 | SHU34 | 0.047 |
| SHU4 | SHU71 | 0.047 |
| SHU23 | SHU61 | 0.047 |
| SHU12 | SHU81 | 0.047 |
| SHU19 | SHU78 | 0.047 |
| SHU23 | SHU76 | 0.048 |
| SHU4 | SHU30 | 0.048 |
| SHU57 | SHU68 | 0.048 |
| SHU40 | SHU60 | 0.048 |
| SHU23 | SHU31 | 0.048 |
| SHU18 | SHU67 | 0.049 |
| SHU48 | SHU67 | 0.049 |
| SHU40 | SHU53 | 0.049 |
| SHU18 | SHU31 | 0.049 |
| SHU31 | SHU48 | 0.049 |
| SHU12 | SHU38 | 0.049 |
| SHU59 | SHU66 | 0.049 |
| SHU19 | SHU55 | 0.049 |
| SHU20 | SHU34 | 0.049 |
| SHU20 | SHU68 | 0.050 |
| SHU6 | SHU14 | 0.050 |
| SHU31 | SHU58 | 0.050 |
| SHU4 | SHU17 | 0.050 |
| SHU4 | SHU24 | 0.050 |
| SHU54 | SHU57 | 0.050 |
| SHU12 | SHU31 | 0.050 |
| SHU41 | SHU54 | 0.051 |
| SHU57 | SHU60 | 0.051 |
| SHU62 | SHU76 | 0.051 |
| SHU27 | SHU82 | 0.051 |
| SHU37 | SHU69 | 0.051 |
| SHU40 | SHU49 | 0.051 |
| SHU41 | SHU62 | 0.051 |
| SHU34 | SHU69 | 0.051 |
| SHU65 | SHU71 | 0.051 |
| SHU60 | SHU68 | 0.052 |
| SHU31 | SHU43 | 0.052 |
| SHU7 | SHU78 | 0.053 |
| SHU6 | SHU22 | 0.053 |
| SHU1 | SHU58 | 0.053 |
| SHU46 | SHU47 | 0.053 |
| SHU50 | SHU54 | 0.053 |
| SHU11 | SHU18 | 0.053 |
| SHU11 | SHU48 | 0.053 |
| SHU76 | SHU82 | 0.053 |
| SHU17 | SHU43 | 0.054 |
| SHU24 | SHU43 | 0.054 |
| SHU36 | SHU60 | 0.054 |
| SHU59 | SHU64 | 0.054 |
| SHU20 | SHU35 | 0.054 |
| SHU14 | SHU58 | 0.054 |
| SHU33 | SHU37 | 0.054 |
| SHU8 | SHU44 | 0.054 |
| SHU41 | SHU70 | 0.054 |
| SHU28 | SHU44 | 0.054 |
| SHU53 | SHU67 | 0.055 |
| SHU10 | SHU79 | 0.055 |
| SHU12 | SHU50 | 0.055 |
| SHU7 | SHU53 | 0.055 |
| SHU4 | SHU5 | 0.055 |
| SHU71 | SHU81 | 0.056 |
| SHU19 | SHU64 | 0.056 |
| SHU18 | SHU49 | 0.056 |
| SHU48 | SHU49 | 0.056 |
| SHU43 | SHU73 | 0.056 |
| SHU20 | SHU21 | 0.057 |
| SHU14 | SHU23 | 0.057 |
| SHU8 | SHU32 | 0.057 |
| SHU43 | SHU51 | 0.057 |
| SHU10 | SHU76 | 0.057 |
| SHU16 | SHU51 | 0.057 |
| SHU20 | SHU37 | 0.057 |
| SHU52 | SHU59 | 0.057 |
| SHU12 | SHU14 | 0.058 |
| SHU49 | SHU58 | 0.058 |
| SHU10 | SHU30 | 0.058 |
| SHU1 | SHU47 | 0.058 |
| SHU40 | SHU42 | 0.058 |
| SHU74 | SHU75 | 0.058 |
| SHU2 | SHU31 | 0.058 |
| SHU23 | SHU44 | 0.058 |
| SHU49 | SHU63 | 0.058 |
| SHU10 | SHU42 | 0.059 |
| SHU20 | SHU75 | 0.059 |
| SHU14 | SHU43 | 0.059 |
| SHU38 | SHU51 | 0.059 |
| SHU34 | SHU57 | 0.059 |
| SHU6 | SHU13 | 0.059 |
| SHU66 | SHU69 | 0.060 |
| SHU57 | SHU73 | 0.061 |
| SHU44 | SHU58 | 0.061 |
| SHU14 | SHU55 | 0.061 |
| SHU5 | SHU15 | 0.062 |
| SHU4 | SHU66 | 0.062 |
| SHU2 | SHU14 | 0.063 |
| SHU30 | SHU61 | 0.063 |
| SHU18 | SHU26 | 0.063 |
| SHU26 | SHU48 | 0.063 |
| SHU36 | SHU65 | 0.064 |
| SHU34 | SHU56 | 0.064 |
| SHU1 | SHU65 | 0.064 |
| SHU8 | SHU50 | 0.064 |
| SHU61 | SHU81 | 0.064 |
| SHU37 | SHU43 | 0.066 |
| SHU3 | SHU72 | 0.066 |
| SHU26 | SHU63 | 0.066 |
| SHU8 | SHU56 | 0.066 |
| SHU2 | SHU59 | 0.067 |
| SHU34 | SHU47 | 0.067 |
| SHU11 | SHU50 | 0.067 |
| SHU5 | SHU21 | 0.067 |
| SHU18 | SHU56 | 0.067 |
| SHU48 | SHU56 | 0.067 |
| SHU40 | SHU41 | 0.067 |
| SHU72 | SHU73 | 0.067 |
| SHU35 | SHU59 | 0.067 |
| SHU18 | SHU53 | 0.067 |
| SHU48 | SHU53 | 0.067 |
| SHU14 | SHU52 | 0.068 |
| SHU65 | SHU70 | 0.068 |
| SHU11 | SHU30 | 0.068 |
| SHU56 | SHU58 | 0.068 |
| SHU7 | SHU72 | 0.069 |
| SHU69 | SHU76 | 0.069 |
| SHU28 | SHU42 | 0.069 |
| SHU2 | SHU25 | 0.069 |
| SHU21 | SHU33 | 0.069 |
| SHU38 | SHU58 | 0.069 |
| SHU2 | SHU13 | 0.070 |
| SHU2 | SHU49 | 0.070 |
| SHU31 | SHU37 | 0.070 |
| SHU3 | SHU4 | 0.070 |
| SHU4 | SHU29 | 0.070 |
| SHU7 | SHU59 | 0.070 |
| SHU65 | SHU67 | 0.070 |
| SHU55 | SHU66 | 0.071 |
| SHU20 | SHU78 | 0.071 |
| SHU10 | SHU78 | 0.071 |
| SHU18 | SHU43 | 0.071 |
| SHU43 | SHU48 | 0.071 |
| SHU61 | SHU71 | 0.072 |
| SHU1 | SHU52 | 0.072 |
| SHU10 | SHU82 | 0.072 |
| SHU21 | SHU26 | 0.072 |
| SHU41 | SHU50 | 0.072 |
| SHU7 | SHU17 | 0.072 |
| SHU7 | SHU24 | 0.072 |
| SHU51 | SHU58 | 0.073 |
| SHU19 | SHU66 | 0.073 |
| SHU55 | SHU78 | 0.073 |
| SHU19 | SHU31 | 0.073 |
| SHU9 | SHU11 | 0.073 |
| SHU40 | SHU62 | 0.073 |
| SHU11 | SHU67 | 0.073 |
| SHU35 | SHU37 | 0.074 |
| SHU7 | SHU49 | 0.074 |
| SHU41 | SHU68 | 0.074 |
| SHU9 | SHU23 | 0.074 |
| SHU40 | SHU47 | 0.074 |
| SHU27 | SHU30 | 0.075 |
| SHU16 | SHU46 | 0.075 |
| SHU19 | SHU59 | 0.075 |
| SHU9 | SHU62 | 0.076 |
| SHU45 | SHU80 | 0.076 |
| SHU55 | SHU73 | 0.076 |
| SHU1 | SHU43 | 0.077 |
| SHU21 | SHU51 | 0.077 |
| SHU53 | SHU55 | 0.077 |
| SHU3 | SHU17 | 0.077 |
| SHU3 | SHU24 | 0.077 |
| SHU28 | SHU35 | 0.078 |
| SHU32 | SHU61 | 0.078 |
| SHU5 | SHU16 | 0.078 |
| SHU33 | SHU59 | 0.078 |
| SHU33 | SHU34 | 0.078 |
| SHU23 | SHU58 | 0.078 |
| SHU21 | SHU37 | 0.078 |
| SHU5 | SHU47 | 0.078 |
| SHU19 | SHU69 | 0.079 |
| SHU15 | SHU65 | 0.079 |
| SHU32 | SHU57 | 0.079 |
| SHU16 | SHU33 | 0.079 |
| SHU5 | SHU27 | 0.079 |
| SHU2 | SHU53 | 0.079 |
| SHU2 | SHU12 | 0.080 |
| SHU4 | SHU51 | 0.081 |
| SHU67 | SHU81 | 0.081 |
| SHU56 | SHU69 | 0.081 |
| SHU7 | SHU74 | 0.081 |
| SHU7 | SHU79 | 0.081 |
| SHU50 | SHU68 | 0.082 |
| SHU62 | SHU66 | 0.082 |
| SHU42 | SHU79 | 0.082 |
| SHU8 | SHU81 | 0.082 |
| SHU27 | SHU69 | 0.083 |
| SHU63 | SHU82 | 0.083 |
| SHU1 | SHU10 | 0.083 |
| SHU13 | SHU52 | 0.084 |
| SHU26 | SHU69 | 0.084 |
| SHU34 | SHU60 | 0.084 |
| SHU2 | SHU43 | 0.084 |
| SHU7 | SHU42 | 0.084 |
| SHU11 | SHU39 | 0.084 |
| SHU46 | SHU63 | 0.084 |
| SHU23 | SHU39 | 0.084 |
| SHU31 | SHU68 | 0.085 |
| SHU34 | SHU54 | 0.085 |
| SHU23 | SHU51 | 0.085 |
| SHU57 | SHU61 | 0.085 |
| SHU27 | SHU75 | 0.085 |
| SHU16 | SHU36 | 0.085 |
| SHU7 | SHU80 | 0.085 |
| SHU14 | SHU37 | 0.085 |
| SHU16 | SHU26 | 0.085 |
| SHU20 | SHU73 | 0.085 |
| SHU29 | SHU43 | 0.086 |
| SHU6 | SHU21 | 0.086 |
| SHU17 | SHU44 | 0.086 |
| SHU24 | SHU44 | 0.086 |
| SHU9 | SHU45 | 0.086 |
| SHU7 | SHU16 | 0.086 |
| SHU1 | SHU17 | 0.087 |
| SHU1 | SHU24 | 0.087 |
| SHU15 | SHU20 | 0.087 |
| SHU8 | SHU38 | 0.087 |
| SHU32 | SHU77 | 0.087 |
| SHU8 | SHU51 | 0.088 |
| SHU10 | SHU62 | 0.088 |
| SHU12 | SHU37 | 0.088 |
| SHU39 | SHU62 | 0.089 |
| SHU34 | SHU35 | 0.089 |
| SHU43 | SHU47 | 0.089 |
| SHU7 | SHU62 | 0.089 |
| SHU61 | SHU67 | 0.089 |
| SHU31 | SHU78 | 0.090 |
| SHU26 | SHU35 | 0.090 |
| SHU32 | SHU69 | 0.091 |
| SHU22 | SHU52 | 0.091 |
| SHU20 | SHU29 | 0.092 |
| SHU27 | SHU78 | 0.092 |
| SHU3 | SHU16 | 0.092 |
| SHU16 | SHU47 | 0.093 |
| SHU18 | SHU27 | 0.093 |
| SHU27 | SHU48 | 0.093 |
| SHU35 | SHU64 | 0.093 |
| SHU27 | SHU51 | 0.093 |
| SHU11 | SHU26 | 0.093 |
| SHU21 | SHU34 | 0.093 |
| SHU62 | SHU78 | 0.093 |
| SHU18 | SHU69 | 0.093 |
| SHU48 | SHU69 | 0.093 |
| SHU41 | SHU42 | 0.094 |
| SHU31 | SHU34 | 0.094 |
| SHU2 | SHU22 | 0.094 |
| SHU4 | SHU28 | 0.094 |
| SHU54 | SHU68 | 0.095 |
| SHU58 | SHU69 | 0.095 |
| SHU30 | SHU32 | 0.095 |
| SHU63 | SHU69 | 0.096 |
| SHU20 | SHU66 | 0.096 |
| SHU1 | SHU15 | 0.097 |
| SHU39 | SHU45 | 0.097 |
| SHU4 | SHU45 | 0.097 |
| SHU18 | SHU82 | 0.097 |
| SHU48 | SHU82 | 0.097 |
| SHU60 | SHU65 | 0.098 |
| SHU5 | SHU52 | 0.099 |
| SHU5 | SHU58 | 0.099 |
| SHU50 | SHU65 | 0.099 |
| SHU6 | SHU16 | 0.099 |
| SHU32 | SHU55 | 0.100 |
| SHU58 | SHU82 | 0.100 |
| SHU36 | SHU40 | 0.102 |
| SHU70 | SHU73 | 0.102 |
| SHU21 | SHU67 | 0.103 |
| SHU7 | SHU46 | 0.103 |
| SHU61 | SHU65 | 0.103 |
| SHU31 | SHU35 | 0.103 |
| SHU58 | SHU72 | 0.104 |
| SHU50 | SHU60 | 0.104 |
| SHU75 | SHU76 | 0.105 |
| SHU5 | SHU43 | 0.106 |
| SHU50 | SHU77 | 0.106 |
| SHU2 | SHU27 | 0.106 |
| SHU26 | SHU52 | 0.107 |
| SHU13 | SHU28 | 0.107 |
| SHU47 | SHU51 | 0.107 |
| SHU29 | SHU46 | 0.107 |
| SHU6 | SHU72 | 0.107 |
| SHU33 | SHU52 | 0.108 |
| SHU21 | SHU31 | 0.108 |
| SHU14 | SHU34 | 0.108 |
| SHU20 | SHU74 | 0.108 |
| SHU7 | SHU8 | 0.108 |
| SHU20 | SHU53 | 0.109 |
| SHU58 | SHU75 | 0.109 |
| SHU35 | SHU53 | 0.110 |
| SHU17 | SHU58 | 0.111 |
| SHU24 | SHU58 | 0.111 |
| SHU15 | SHU35 | 0.111 |
| SHU15 | SHU19 | 0.111 |
| SHU25 | SHU57 | 0.111 |
| SHU36 | SHU50 | 0.111 |
| SHU53 | SHU64 | 0.111 |
| SHU43 | SHU53 | 0.112 |
| SHU21 | SHU52 | 0.113 |
| SHU12 | SHU16 | 0.114 |
| SHU5 | SHU10 | 0.114 |
| SHU47 | SHU58 | 0.115 |
| SHU29 | SHU35 | 0.116 |
| SHU11 | SHU47 | 0.116 |
| SHU14 | SHU35 | 0.116 |
| SHU34 | SHU37 | 0.117 |
| SHU13 | SHU22 | 0.117 |
| SHU21 | SHU72 | 0.117 |
| SHU7 | SHU77 | 0.117 |
| SHU16 | SHU43 | 0.117 |
| SHU35 | SHU51 | 0.118 |
| SHU4 | SHU12 | 0.118 |
| SHU10 | SHU29 | 0.118 |
| SHU5 | SHU17 | 0.119 |
| SHU5 | SHU24 | 0.119 |
| SHU2 | SHU72 | 0.119 |
| SHU23 | SHU45 | 0.120 |
| SHU10 | SHU21 | 0.120 |
| SHU13 | SHU80 | 0.120 |
| SHU19 | SHU60 | 0.120 |
| SHU18 | SHU46 | 0.121 |
| SHU46 | SHU48 | 0.121 |
| SHU70 | SHU78 | 0.121 |
| SHU10 | SHU63 | 0.121 |
| SHU29 | SHU64 | 0.121 |
| SHU11 | SHU23 | 0.122 |
| SHU14 | SHU67 | 0.122 |
| SHU41 | SHU47 | 0.122 |
| SHU11 | SHU14 | 0.122 |
| SHU29 | SHU55 | 0.122 |
| SHU25 | SHU60 | 0.123 |
| SHU50 | SHU55 | 0.123 |
| SHU2 | SHU74 | 0.123 |
| SHU35 | SHU46 | 0.123 |
| SHU2 | SHU80 | 0.124 |
| SHU21 | SHU29 | 0.125 |
| SHU7 | SHU37 | 0.125 |
| SHU46 | SHU53 | 0.125 |
| SHU16 | SHU20 | 0.126 |
| SHU32 | SHU74 | 0.126 |
| SHU16 | SHU58 | 0.126 |
| SHU2 | SHU17 | 0.126 |
| SHU2 | SHU24 | 0.126 |
| SHU7 | SHU22 | 0.126 |
| SHU16 | SHU52 | 0.127 |
| SHU7 | SHU34 | 0.127 |
| SHU17 | SHU21 | 0.128 |
| SHU21 | SHU24 | 0.128 |
| SHU28 | SHU52 | 0.128 |
| SHU26 | SHU79 | 0.128 |
| SHU10 | SHU69 | 0.128 |
| SHU29 | SHU38 | 0.129 |
| SHU75 | SHU81 | 0.129 |
| SHU1 | SHU35 | 0.129 |
| SHU9 | SHU39 | 0.130 |
| SHU4 | SHU27 | 0.130 |
| SHU19 | SHU34 | 0.130 |
| SHU29 | SHU63 | 0.131 |
| SHU63 | SHU67 | 0.131 |
| SHU55 | SHU77 | 0.132 |
| SHU14 | SHU72 | 0.132 |
| SHU12 | SHU80 | 0.132 |
| SHU32 | SHU50 | 0.133 |
| SHU15 | SHU31 | 0.133 |
| SHU34 | SHU68 | 0.134 |
| SHU23 | SHU64 | 0.134 |
| SHU17 | SHU22 | 0.135 |
| SHU22 | SHU24 | 0.135 |
| SHU69 | SHU82 | 0.136 |
| SHU40 | SHU45 | 0.136 |
| SHU38 | SHU53 | 0.139 |
| SHU43 | SHU46 | 0.140 |
| SHU3 | SHU63 | 0.140 |
| SHU31 | SHU76 | 0.141 |
| SHU19 | SHU54 | 0.142 |
| SHU2 | SHU16 | 0.143 |
| SHU72 | SHU75 | 0.143 |
| SHU50 | SHU74 | 0.144 |
| SHU73 | SHU76 | 0.144 |
| SHU29 | SHU31 | 0.145 |
| SHU31 | SHU66 | 0.145 |
| SHU2 | SHU46 | 0.145 |
| SHU16 | SHU37 | 0.145 |
| SHU52 | SHU72 | 0.146 |
| SHU14 | SHU17 | 0.146 |
| SHU14 | SHU24 | 0.146 |
| SHU35 | SHU52 | 0.146 |
| SHU13 | SHU44 | 0.146 |
| SHU4 | SHU23 | 0.147 |
| SHU43 | SHU55 | 0.147 |
| SHU29 | SHU76 | 0.148 |
| SHU9 | SHU13 | 0.149 |
| SHU2 | SHU33 | 0.150 |
| SHU25 | SHU50 | 0.151 |
| SHU11 | SHU63 | 0.151 |
| SHU7 | SHU76 | 0.152 |
| SHU29 | SHU67 | 0.153 |
| SHU11 | SHU45 | 0.153 |
| SHU2 | SHU77 | 0.153 |
| SHU7 | SHU21 | 0.153 |
| SHU27 | SHU43 | 0.154 |
| SHU23 | SHU29 | 0.155 |
| SHU7 | SHU29 | 0.155 |
| SHU29 | SHU47 | 0.155 |
| SHU44 | SHU67 | 0.156 |
| SHU44 | SHU71 | 0.156 |
| SHU11 | SHU51 | 0.157 |
| SHU7 | SHU33 | 0.158 |
| SHU4 | SHU10 | 0.158 |
| SHU28 | SHU47 | 0.158 |
| SHU2 | SHU8 | 0.159 |
| SHU13 | SHU17 | 0.160 |
| SHU13 | SHU24 | 0.160 |
| SHU5 | SHU35 | 0.160 |
| SHU12 | SHU51 | 0.160 |
| SHU55 | SHU72 | 0.161 |
| SHU2 | SHU37 | 0.162 |
| SHU14 | SHU76 | 0.162 |
| SHU11 | SHU13 | 0.163 |
| SHU31 | SHU53 | 0.163 |
| SHU13 | SHU39 | 0.163 |
| SHU14 | SHU29 | 0.164 |
| SHU57 | SHU71 | 0.165 |
| SHU12 | SHU34 | 0.165 |
| SHU6 | SHU10 | 0.165 |
| SHU9 | SHU12 | 0.166 |
| SHU3 | SHU18 | 0.167 |
| SHU3 | SHU48 | 0.167 |
| SHU53 | SHU76 | 0.168 |
| SHU4 | SHU8 | 0.168 |
| SHU21 | SHU35 | 0.168 |
| SHU23 | SHU53 | 0.170 |
| SHU58 | SHU63 | 0.170 |
| SHU7 | SHU56 | 0.171 |
| SHU2 | SHU10 | 0.171 |
| SHU13 | SHU23 | 0.171 |
| SHU2 | SHU34 | 0.173 |
| SHU16 | SHU34 | 0.175 |
| SHU45 | SHU53 | 0.175 |
| SHU3 | SHU15 | 0.175 |
| SHU6 | SHU18 | 0.177 |
| SHU6 | SHU48 | 0.177 |
| SHU35 | SHU43 | 0.177 |
| SHU27 | SHU46 | 0.179 |
| SHU10 | SHU13 | 0.181 |
| SHU12 | SHU39 | 0.181 |
| SHU8 | SHU80 | 0.184 |
| SHU6 | SHU63 | 0.184 |
| SHU4 | SHU44 | 0.184 |
| SHU4 | SHU11 | 0.185 |
| SHU12 | SHU23 | 0.186 |
| SHU14 | SHU53 | 0.186 |
| SHU27 | SHU53 | 0.186 |
| SHU16 | SHU35 | 0.187 |
| SHU16 | SHU31 | 0.189 |
| SHU35 | SHU47 | 0.189 |
| SHU2 | SHU63 | 0.190 |
| SHU27 | SHU35 | 0.191 |
| SHU11 | SHU16 | 0.192 |
| SHU7 | SHU18 | 0.192 |
| SHU7 | SHU48 | 0.192 |
| SHU7 | SHU58 | 0.195 |
| SHU10 | SHU27 | 0.195 |
| SHU22 | SHU79 | 0.195 |
| SHU25 | SHU41 | 0.195 |
| SHU45 | SHU49 | 0.197 |
| SHU2 | SHU76 | 0.197 |
| SHU43 | SHU74 | 0.199 |
| SHU49 | SHU53 | 0.201 |
| SHU21 | SHU47 | 0.202 |
| SHU21 | SHU27 | 0.202 |
| SHU19 | SHU25 | 0.203 |
| SHU42 | SHU64 | 0.205 |
| SHU16 | SHU72 | 0.205 |
| SHU2 | SHU56 | 0.206 |
| SHU19 | SHU68 | 0.207 |
| SHU43 | SHU75 | 0.209 |
| SHU59 | SHU69 | 0.211 |
| SHU33 | SHU35 | 0.211 |
| SHU72 | SHU74 | 0.214 |
| SHU20 | SHU72 | 0.214 |
| SHU18 | SHU52 | 0.215 |
| SHU48 | SHU52 | 0.215 |
| SHU42 | SHU47 | 0.217 |
| SHU13 | SHU45 | 0.218 |
| SHU10 | SHU22 | 0.218 |
| SHU10 | SHU52 | 0.218 |
| SHU27 | SHU63 | 0.219 |
| SHU19 | SHU57 | 0.222 |
| SHU52 | SHU63 | 0.222 |
| SHU16 | SHU17 | 0.223 |
| SHU16 | SHU24 | 0.223 |
| SHU14 | SHU63 | 0.223 |
| SHU8 | SHU9 | 0.224 |
| SHU35 | SHU58 | 0.225 |
| SHU74 | SHU76 | 0.226 |
| SHU31 | SHU55 | 0.227 |
| SHU18 | SHU58 | 0.227 |
| SHU48 | SHU58 | 0.227 |
| SHU29 | SHU58 | 0.229 |
| SHU52 | SHU69 | 0.233 |
| SHU7 | SHU82 | 0.234 |
| SHU17 | SHU72 | 0.234 |
| SHU24 | SHU72 | 0.234 |
| SHU8 | SHU11 | 0.236 |
| SHU25 | SHU54 | 0.237 |
| SHU12 | SHU45 | 0.237 |
| SHU18 | SHU21 | 0.239 |
| SHU21 | SHU48 | 0.239 |
| SHU7 | SHU63 | 0.240 |
| SHU27 | SHU47 | 0.241 |
| SHU6 | SHU52 | 0.241 |
| SHU8 | SHU39 | 0.242 |
| SHU14 | SHU20 | 0.243 |
| SHU2 | SHU82 | 0.243 |
| SHU10 | SHU17 | 0.246 |
| SHU10 | SHU24 | 0.246 |
| SHU8 | SHU23 | 0.246 |
| SHU35 | SHU69 | 0.252 |
| SHU25 | SHU34 | 0.253 |
| SHU2 | SHU18 | 0.254 |
| SHU2 | SHU48 | 0.254 |
| SHU7 | SHU27 | 0.254 |
| SHU2 | SHU58 | 0.257 |
| SHU74 | SHU77 | 0.259 |
| SHU55 | SHU76 | 0.260 |
| SHU14 | SHU18 | 0.262 |
| SHU14 | SHU48 | 0.262 |
| SHU4 | SHU13 | 0.265 |
| SHU53 | SHU58 | 0.267 |
| SHU20 | SHU43 | 0.270 |
| SHU63 | SHU72 | 0.274 |
| SHU14 | SHU21 | 0.274 |
| SHU43 | SHU76 | 0.275 |
| SHU43 | SHU58 | 0.276 |
| SHU61 | SHU79 | 0.278 |
| SHU37 | SHU64 | 0.279 |
| SHU38 | SHU42 | 0.283 |
| SHU2 | SHU69 | 0.284 |
| SHU36 | SHU51 | 0.288 |
| SHU6 | SHU17 | 0.288 |
| SHU6 | SHU24 | 0.288 |
| SHU25 | SHU68 | 0.292 |
| SHU57 | SHU59 | 0.292 |
| SHU17 | SHU63 | 0.292 |
| SHU24 | SHU63 | 0.292 |
| SHU72 | SHU76 | 0.293 |
| SHU33 | SHU69 | 0.294 |
| SHU7 | SHU69 | 0.295 |
| SHU67 | SHU71 | 0.295 |
| SHU46 | SHU58 | 0.299 |
| SHU11 | SHU21 | 0.301 |
| SHU8 | SHU45 | 0.304 |
| SHU38 | SHU64 | 0.307 |
| SHU23 | SHU38 | 0.308 |
| SHU7 | SHU10 | 0.308 |
| SHU18 | SHU72 | 0.311 |
| SHU48 | SHU72 | 0.311 |
| SHU12 | SHU13 | 0.313 |
| SHU14 | SHU31 | 0.315 |
| SHU16 | SHU63 | 0.319 |
| SHU20 | SHU76 | 0.323 |
| SHU55 | SHU74 | 0.325 |
| SHU20 | SHU55 | 0.325 |
| SHU11 | SHU12 | 0.326 |
| SHU27 | SHU58 | 0.332 |
| SHU17 | SHU18 | 0.333 |
| SHU18 | SHU24 | 0.333 |
| SHU17 | SHU48 | 0.333 |
| SHU24 | SHU48 | 0.333 |
| SHU43 | SHU72 | 0.337 |
| SHU29 | SHU53 | 0.337 |
| SHU1 | SHU6 | 0.350 |
| SHU33 | SHU46 | 0.355 |
| SHU21 | SHU63 | 0.357 |
| SHU16 | SHU18 | 0.362 |
| SHU16 | SHU48 | 0.362 |
| SHU27 | SHU29 | 0.373 |
| SHU16 | SHU21 | 0.376 |
| SHU17 | SHU52 | 0.378 |
| SHU24 | SHU52 | 0.378 |
| SHU14 | SHU16 | 0.381 |
| SHU5 | SHU6 | 0.381 |
| SHU8 | SHU13 | 0.390 |
| SHU20 | SHU31 | 0.403 |
| SHU8 | SHU12 | 0.413 |
| SHU2 | SHU7 | 0.437 |
| SHU18 | SHU63 | 0.451 |
| SHU48 | SHU63 | 0.451 |
| SHU56 | SHU80 | 0.462 |
| SHU1 | SHU5 | 0.462 |
| SHU17 | SHU24 | 0.500 |
| SHU18 | SHU48 | 0.500 |
