# Supplementary material for: Reduced genetic variability in a captive-bred population of the endangered Hume’s pheasant (Syrmaticus humiae, Hume 1881) revealed by microsatellite genotyping and D-loop sequencing
Source: PLoS One. 2021 Aug 27;16(8):e0256573. doi: 10.1371/journal.pone.0256573 (PMC8396778; doi:10.1371/journal.pone.0256573)
Supplement: S9 Table — Detailed information for all individuals is presented in S1 Table. (DOCX) [file pone.0256573.s009.docx]

**S9 Table Pairwise inbreeding coefficients (*F*_IS_) for 82** **Hume’s pheasant (*Syrmaticus humiae,* Hume 1881) individuals.** Detailed information for all individuals is presented in S1 Table.

| **Individual** | ***F*_IS_** | **Individual** | ***F*_IS_** |
| --- | --- | --- | --- |
| SHU1 | 0.1311 | SHU42 | 0.1253 |
| SHU2 | 0.0629 | SHU43 | 0.0266 |
| SHU3 | 0.2393 | SHU44 | 0.0690 |
| SHU4 | 0.2487 | SHU45 | 0.1174 |
| SHU5 | 0.1164 | SHU46 | 0.0089 |
| SHU6 | 0.1045 | SHU47 | 0.1021 |
| SHU7 | -0.0029 | SHU48 | 0.0578 |
| SHU8 | 0.0668 | SHU49 | 0.0543 |
| SHU9 | 0.0994 | SHU50 | 0.0970 |
| SHU10 | 0.0565 | SHU51 | 0.0701 |
| SHU11 | 0.1514 | SHU52 | 0.0810 |
| SHU12 | 0.0819 | SHU53 | 0.0481 |
| SHU13 | 0.1031 | SHU54 | 0.4049 |
| SHU14 | 0.0693 | SHU55 | 0.1800 |
| SHU15 | 0.4874 | SHU56 | 0.0651 |
| SHU16 | 0.0506 | SHU57 | 0.5811 |
| SHU17 | 0.0606 | SHU58 | 0.0519 |
| SHU18 | 0.0547 | SHU59 | 0.1047 |
| SHU19 | 0.2920 | SHU60 | 0.3141 |
| SHU20 | 0.1180 | SHU61 | 0.2382 |
| SHU21 | 0.0549 | SHU62 | 0.0268 |
| SHU22 | 0.0513 | SHU63 | 0.0131 |
| SHU23 | 0.1807 | SHU64 | 0.2888 |
| SHU24 | 0.0570 | SHU65 | 0.4632 |
| SHU25 | 0.0900 | SHU66 | 0.3233 |
| SHU26 | 0.0027 | SHU67 | 0.2386 |
| SHU27 | 0.0676 | SHU68 | 0.3861 |
| SHU28 | 0.2462 | SHU69 | 0.0556 |
| SHU29 | 0.0596 | SHU70 | 0.1128 |
| SHU30 | 0.1034 | SHU71 | 0.1482 |
| SHU31 | 0.0592 | SHU72 | 0.0747 |
| SHU32 | 0.1095 | SHU73 | 0.6687 |
| SHU33 | 0.0136 | SHU74 | 0.0552 |
| SHU34 | 0.0683 | SHU75 | 0.1076 |
| SHU35 | 0.0643 | SHU76 | 0.0772 |
| SHU36 | 0.5225 | SHU77 | 0.0097 |
| SHU37 | 0.0831 | SHU78 | 0.0405 |
| SHU38 | 0.2082 | SHU79 | 0.1132 |
| SHU39 | 0.1658 | SHU80 | 0.0668 |
| SHU40 | 1.1490 | SHU81 | 0.1200 |
| SHU41 | 0.4298 | SHU82 | 0.0075 |
