# Supplementary material for: Reduced genetic variability in a captive-bred population of the endangered Hume’s pheasant (Syrmaticus humiae, Hume 1881) revealed by microsatellite genotyping and D-loop sequencing
Source: PLoS One. 2021 Aug 27;16(8):e0256573. doi: 10.1371/journal.pone.0256573 (PMC8396778; doi:10.1371/journal.pone.0256573)
Supplement: S10 Table — Detailed information for all individuals is presented in S2 Table. (DOCX) [file pone.0256573.s010.docx]

**S10 Table** **Parentage analysis of 82 Hume’s pheasant (*Syrmaticus humiae,* Hume 1881) individuals.** Detailed information for all individuals is presented in S2 Table.

| FullSibship  Index | Prob  (Inc.) | Prob  (Exc.) | Member1 | Member2 | Member3 | Member4 | Member5 | Member6 |
| --- | --- | --- | --- | --- | --- | --- | --- | --- |
| 1 | 0.8289 | 0.1009 | SHU2 | SHU7 | SHU82 | SHU18 | SHU48 | SHU63 |
| 2 | 1.0000 | 0.2774 | SHU4 | SHU44 |  |  |  |  |
| 3 | 0.9264 | 0.4972 | SHU8 | SHU13 | SHU45 | SHU80 | SHU56 |  |
| 4 | 0.9832 | 0.0467 | SHU11 | SHU12 |  |  |  |  |
| 5 | 0.6335 | 0.0773 | SHU14 | SHU16 | SHU16 | SHU31 | SHU72 |  |
| 6 | 0.9603 | 0.4331 | SHU15 | SHU3 |  |  |  |  |
| 7 | 1.0000 | 0.2067 | SHU19 | SHU57 |  |  |  |  |
| 8 | 0.9161 | 0.0809 | SHU22 | SHU10 |  |  |  |  |
| 9 | 1.0000 | 0.1214 | SHU27 | SHU29 | SHU53 |  |  |  |
| 10 | 1.0000 | 0.0591 | SHU30 |  |  |  |  |  |
| 11 | 0.5485 | 0.4845 | SHU32 | SHU50 |  |  |  |  |
| 12 | 0.8816 | 0.0932 | SHU33 | SHU46 | SHU58 | SHU69 |  |  |
| 13 | 0.4892 | 0.1352 | SHU34 | SHU68 | SHU25 |  |  |  |
| 14 | 0.9918 | 0.3451 | SHU36 | SHU51 |  |  |  |  |
| 15 | 1.0000 | 0.8203 | SHU38 | SHU64 | SHU23 |  |  |  |
| 16 | 1.0000 | 0.1286 | SHU39 | SHU9 |  |  |  |  |
| 17 | 1.0000 | 0.0620 | SHU42 |  |  |  |  |  |
| 18 | 0.9346 | 0.0908 | SHU43 | SHU75 |  |  |  |  |
| 19 | 0.5383 | 0.1092 | SHU47 | SHU21 | SHU35 |  |  |  |
| 20 | 1.0000 | 0.0840 | SHU49 |  |  |  |  |  |
| 21 | 1.0000 | 0.0910 | SHU59 |  |  |  |  |  |
| 22 | 1.0000 | 0.1928 | SHU66 |  |  |  |  |  |
| 23 | 0.9980 | 0.5326 | SHU71 | SHU67 |  |  |  |  |
| 24 | 0.9857 | 0.1370 | SHU74 | SHU76 | SHU55 | SHU77 |  |  |
| 25 | 0.7584 | 0.7471 | SHU78 | SHU62 |  |  |  |  |
| 26 | 1.0000 | 0.0730 | SHU1 | SHU5 | SHU6 |  |  |  |
| 27 | 0.8062 | 0.0502 | SHU17 | SHU24 | SHU52 |  |  |  |
| 28 | 1.0000 | 0.0925 | SHU26 |  |  |  |  |  |
| 29 | 1.0000 | 0.0969 | SHU28 |  |  |  |  |  |
| 30 | 1.0000 | 0.0554 | SHU37 |  |  |  |  |  |
| 31 | 1.0000 | 0.3030 | SHU40 |  |  |  |  |  |
| 32 | 1.0000 | 0.0768 | SHU41 |  |  |  |  |  |
| 33 | 1.0000 | 0.1418 | SHU54 |  |  |  |  |  |
| 34 | 1.0000 | 0.1236 | SHU60 |  |  |  |  |  |
| 35 | 0.6572 | 0.3443 | SHU61 | SHU79 |  |  |  |  |
| 36 | 1.0000 | 0.0951 | SHU65 |  |  |  |  |  |
| 37 | 1.0000 | 0.1713 | SHU70 |  |  |  |  |  |
| 38 | 1.0000 | 0.1133 | SHU73 |  |  |  |  |  |
| 39 | 1.0000 | 0.0957 | SHU81 |  |  |  |  |  |
