# Supplementary material for: Reduced genetic variability in a captive-bred population of the endangered Hume’s pheasant (Syrmaticus humiae, Hume 1881) revealed by microsatellite genotyping and D-loop sequencing
Source: PLoS One. 2021 Aug 27;16(8):e0256573. doi: 10.1371/journal.pone.0256573 (PMC8396778; doi:10.1371/journal.pone.0256573)
Supplement: S11 Table — Detailed information for all individuals is presented in S2 Table. (DOCX) [file pone.0256573.s011.docx]

**S11 Table Probability of identity estimated using Gimlet version 1.3.3 (Valière, 2002) of Hume’s pheasant (*Syrmaticus humiae,* Hume 1881) individuals based on 12 microsatellite loci.** Detailed information for all individuals is presented in S2 Table.

| Locus | Unbias/loc. | Prod (unbias) |
| --- | --- | --- |
| shul35 | 6.84E-01 | 6.84E-01 |
| shul52 | 9.27E-01 | 6.35E-01 |
| shul36 | 1.05E-01 | 6.64E-02 |
| shul54 | 4.07E-01 | 2.70E-02 |
| shull67 | 7.32E-02 | 1.98E-03 |
| shull108 | 4.24E-01 | 8.39E-04 |
| shul50 | 1.23E-01 | 1.03E-04 |
| shul51 | 6.63E-01 | 6.84E-05 |
| shul62 | 2.07E-01 | 1.41E-05 |
| Shoul15 | 1.22E-01 | 1.72E-06 |
| ShuI16 | 6.17E-01 | 1.06E-06 |
| ShuI22 | 3.52E-01 | 3.74E-07 |
| Mean | 0.392 | 0.118 |
| S.D. | 0.2801 | 0.254 |

Valière N, 2002. GIMLET: a computer program for analysing genetic individual identification data. *Mol Ecol Notes* 2:377–379.
