# Supplementary material for: Reduced genetic variability in a captive-bred population of the endangered Hume’s pheasant (Syrmaticus humiae, Hume 1881) revealed by microsatellite genotyping and D-loop sequencing
Source: PLoS One. 2021 Aug 27;16(8):e0256573. doi: 10.1371/journal.pone.0256573 (PMC8396778; doi:10.1371/journal.pone.0256573)
Supplement: S12 Table — (DOCX) [file pone.0256573.s012.docx]

**S12 Table** **Mitochondrial D-loop sequence diversity for the** **Hume’s pheasant (*Syrmaticus humiae,* Hume 1881) population.**

| Population | Sample size | Number of haplotypes (*H*) | Haplotype diversity (*h*) | Nucleotide diversity (*π*) | Theta (per site) from S | Average number of nucleotide  differences (*k*) |
| --- | --- | --- | --- | --- | --- | --- |
| All population | 82 | 4 | 0.365 ± 0.004 | 0.006 ± 0.004 | 0.004 | 0.966 |
