# Supplementary material for: Reduced genetic variability in a captive-bred population of the endangered Hume’s pheasant (Syrmaticus humiae, Hume 1881) revealed by microsatellite genotyping and D-loop sequencing
Source: PLoS One. 2021 Aug 27;16(8):e0256573. doi: 10.1371/journal.pone.0256573 (PMC8396778; doi:10.1371/journal.pone.0256573)
Supplement: S13 Table — (DOCX) [file pone.0256573.s013.docx]

**S13 Table** **Neutrality tests of mitochondrial D-loop sequence for the Hume’s pheasant (*Syrmaticus humiae,* Hume 1881) population.**

| Population | | Tajima’s *D** | Fu’s *D** | Fu’s *F** | Fu's *F*_s_ | Ewens-Watterson test | Chakraborty’s test | Ramos-Onsins and Rozas’s *R*_2_ | Raggedness index |
| --- | --- | --- | --- | --- | --- | --- | --- | --- | --- |
| DTP | 1.123^ns^ | | 0.844^ns^ | 1.090^ns^ | 1.198^ns^ | 0.686^ns^ | 0.293^ns^ | 0.161^ns^ | 0.480^ns^ |

ns = not significant
